# Electrochemical Nitrate and Nitrite Reduction Reaction to Ammonia: Catalytic Aging and Stability of Co_3_O_4_ Hexagonal Nanoplates

**DOI:** 10.1021/acsami.6c03616

**Published:** 2026-05-20

**Authors:** Matheus P. Sales, Maykon L. Souza, Marionir C. Branco, Samuel C. Silva, João P. B. Da Silva, Rafael G. Yoshimura, Rafael L. Romano, João J. Mauricio, Manuel E. G. Winkler, Kauan L. Gomes, João B. Souza, Santiago J. A. Figueroa, Fabio H. B. Lima, Cláudio F. Tormena, Juarez L. F. Da Silva, Italo O. Mazali, Raphael Nagao

**Affiliations:** † Institute of Chemistry, 28132 University of Campinas, Campinas, São Paulo 13083-862, Brazil; ‡ Center for Innovation on New Energies, University of Campinas, Campinas, São Paulo 13083-084, Brazil; § Department of Chemistry, Federal University of São Carlos, São Carlos, São Paulo 13565-905, Brazil; ∥ São Carlos Institute of Chemistry, University of São Paulo, São Carlos, São Paulo 13566-590, Brazil; ⊥ Brazilian Synchrotron Light Laboratory, Brazilian Center for Research in Energy and Materials, Campinas, São Paulo 13083-100, Brazil; # Brazilian Nanotechnology National Laboratory, Brazilian Center for Research in Energy and Materials, Campinas, São Paulo 13083-100, Brazil

**Keywords:** electrocatalysis, nitrate/nitrite reduction, ammonia, cobalt nanoplates

## Abstract

The nitrate reduction reaction (NO_3_RR) offers a sustainable route to ammonia under ambient conditions, yet its practical implementation is hindered by sluggish kinetics and poor understanding of catalyst evolution under bias. Here, we used Co_3_O_4_ nanoplates as model electrocatalysts for NO_3_RR and NO_2_RR, and systematically evaluated their morphological and structural evolution after an accelerated electrochemical aging protocol (1000 CV cycles). By comparing pristine and aged electrodes, we probe changes in charge-transfer dynamics, interfacial resistance, surface defect density, nanoparticle aggregation, and the Co^2+^/Co^3+^ ratio, using in situ and ex situ electrochemical and spectroscopic techniques. Density functional theory calculations provide insights into the evolution of surface energetics and the adsorption behavior of key intermediates. The aged catalyst exhibits faster charge-transfer kinetics and lower resistance, associated with surface reconstruction and modified adsorption properties. Specifically, weakened *NO_2_ adsorption and a more thermoneutral *H binding promote hydrogen evolution (HER), shifting product selectivity. Although the Faradaic efficiency toward NH_3_ of the aged surface decreases, higher yield rates are achieved due to enhanced kinetics. These findings underscore that dynamic changes in Co_3_O_4_ morphology and surface chemistry govern the stabilization of intermediates and performance over time. Understanding such dynamic behavior provides a foundation for the rational design of catalysts and efficient NO_3_^–^ usage.

## Graphical abstract

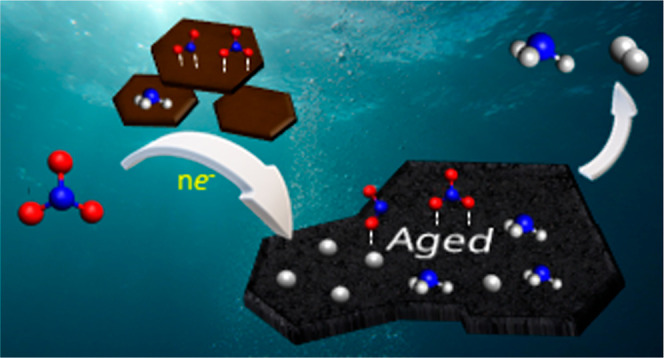

## 1 Introduction

In pursuit of a sustainable environment, society’s economy has begun transitioning into a new era of decarbonized technologies.^1,2^ In this context, ammonia (NH_3_) emerges as a promising candidate for a carbon-free energy carrier due to its high energy density (4.32 kWh L^–1^), hydrogen content (17.65 wt %), low boiling point (−33.3 °C at 1 atm), and mature transport infrastructure.^3–6^ The latter is attributed to the well-established Haber-Bosch (HB) process, which synthesizes NH_3_ on an industrial scale from H_2_ and N_2_, involving harsh operating conditions such as elevated temperatures (400–500 °C) and pressures (150–300 atm).^7–9^ Furthermore, the process uses H_2_ produced from steam-methane reforming route, requiring even higher temperatures (700–1000 °C) but lower pressures (3–25 atm) to convert CH_4_ into H_2_ and CO.^10^ As a result, the large-scale use of the HB process contributes to nearly 2% of global greenhouse gas emissions, 1–2% of the world’s energy supply, and 1% of total energy-related CO_2_ releases.^2,11–13^ To tackle this issue, many research groups are investigating the electrochemical synthesis of NH_3_ since it can employ renewable electricity sources, such as wind,^14^ solar,^15^ and hydroelectric power.^16^

Most of the atmosphere is composed of N_2_ (78%), prompting researchers to work on the nitrogen reduction reaction by using water as a H atom donor, thus avoiding the need for H_2_ direct usage.^17^ However, the N≡N bond energy (941 kJ mol^–1^) and the N_2_ low solubility in water (0.66 mmol L^–1^) hinder the reaction’s efficiency.^13,18,19^ Alternatively, the nitrate reduction reaction (NO_3_RR) circumvents both issues given its lower N=O bond energy (204 kJ mol^–1^) and higher solubility (>2 mol L^–1^), promoting the reaction’s performance.^20^ NO_3_^–^ is classified as a pollutant in aqueous environments by the World Health Organization due to anthropogenic activities, leading to health complications such as methemoglobinemia, increased heart rate, nausea, blue baby syndrome, liver damage, and cancer.^21^ NO_3_RR, in turn, is a complex 8-electron process. The rate-determining step is considered to be the reduction of adsorbed NO_3_^–^ to NO_2_^–^.^22,23^ The latter then reduces to an intermediate NO which, for low selectivity catalysts, lead to other byproducts such as N_2_O and N_2_ depending on the NO adsorption site.^24,25^ Two proposed pathways for NH_3_ have been repeatedly mentioned in the literature, the first being the Eley–Rideal proton-coupled electron transfer, forming NH_2_OH as an intermediate,^26–30^ and the Langmuir–Hinshelwood hydrogenation of adsorbed N into NH_3_.^31–33^ Overall, it is necessary to develop electrocatalysts with high activity and selectivity toward NH_3_ in order to commercially enable the NO_3_RR.

Various materials have been proposed as electrocatalysts for NO_3_RR, such as transition metal oxides,^34,35^ single atom catalysts,^36^ metal carbides (MXenes),^37^ high entropy alloys^38^ and phthalocyanine-based systems.^39^ Particularly, transition metals show great potential to be used as cost-effective catalysts, e.g., nitrogen-doped iron,^40^ FeNi/graphitized carbon,^41^ Cu_2_O nanocubes,^27^ and many others. Carvalho et al. investigated the role of electronic structure on NO_3_RR for polycrystalline transition metal foils in neutral pH.^42^ Their selectivity was mapped according to both NO_2_^–^ reduction and NO dissociation energies, where density functional theory (DFT) analysis suggested that Co has an optimal selectivity for NH_3_ production among other transition metals.

The catalyst crystal structure plays an important role for the reaction efficiency. Spinel oxides have been used in various catalytic reactions, such as oxygen reduction reaction and oxygen evolution reaction (OER).^43^ This class of oxides has a general chemical formula AB_2_O_4_, which belongs to the *Fd*3̅*m* space group, where A and B occupy the tetrahedral and octahedral sites, respectively, and the anion is located in the vertices.^43,44^ Oxides generally have higher stability for operando conditions compared to other reduced species, but they also exhibit limited active site exposure and poor electrical conductivity, making bulk spinel applications less explored.^45^ For that, one strategy is to increase the surface-area-to-volume ratio, which can be done by turning the oxides into nanostructures, which have been extensively investigated in the literature, some examples being nanoflakes,^46^ nanorods,^47^ nanoflowers,^48^ nanocubes,^49^ and nanowires.^50^ The increase in surface area to volume ratio exposes more active sites to the reaction compared to the metal foil. Defect engineering is also another strategy for modulating the performance of electrocatalysts. Recently great progress has been made in the synthesis of catalysts with point defects such as heteroatoms,^51^ vacancies,^52^ and etchings,^53^ as well as line, planar and volume defects.^44^ These modifications change the electron configuration of the catalyst, promoting reactions with intermediates, often leading to defects being treated as active sites for electrochemical processes.

Herein, we report a simple hydrothermal treatment followed by calcination to prepare the Co_3_O_4_ spinel nanoplate catalyst. After the calcination treatment, some Co^2+^ tetrahedral sites oxidize to Co^3+^ octahedral interstices, being Co^2+^ able to form weak π interactions with the oxygen p orbital, while the Co^3+^ forms a strong σ interaction, allowing NO_3_^–^ to bind to the catalyst surface and undergo further hydrogenation.^54,55^ The catalyst efficiency for NO_3_RR and NO_2_RR were investigated by successive chronoamperometric experiments. The study of NO_2_^–^ reduction is also important since this species is the main reaction intermediate to ammonia formation in the NO_3_RR. A maximum Faradaic efficiency (FE) for ammonia formation of 92.5 ± 7.5% was observed at –0.2 V vs reversible hydrogen electrode (RHE) in 1 h of electrolysis and yield rate (YR) of 45.6 ± 4.1 μmol h^–1^ cm^–2^ at −0.5 V vs RHE in the presence of NO_2_^–^. NO_3_RR, on the other hand, achieved FE of 49.4 ± 0.5% and YR of 8.5 ± 0.6 μmol h^–1^ cm^–2^ at −0.3 V vs RHE. In order to verify how voltammetric cycling could affect the electrochemical performance, i.e., simulating an accelerated catalyst aging, the Co_3_O_4_ nanoplates (Co_3_O_4_-np) were aged by 1000 cycles at 20 mV s^–1^ between 0.1 and −0.6 V vs RHE. The selectivity for NH_3_ formation decreased to 23.8% on average, but YR was enhanced by up to 5-fold at −0.4 V vs RHE. This could be attributed to the formation of surface defects, sintering, and amorphization on the catalyst’s borders as observed by high-resolution electronic micrographs, while still preserving the Co_3_O_4_ spinel phase. The distribution of relaxation times (DRT) derived from impedance data demonstrated smaller resistances associated with the charge-transfer and increased kinetics, which agrees with larger YR. DFT calculations evaluated both NO_3_RR and hydrogen evolution reaction (HER) thermodynamic landscapes for pristine and aged Co_3_O_4_, suggesting the oxidized surface excels in stabilizing the *H intermediate. This is further corroborated by electrochemical mass spectroscopy (EC-MS) and gas chromatography (GC) experiments, which show higher activity and selectivity for H_2_ after the 1000 cycles treatment. These findings highlight that changes in the morphology and surface chemistry of Co_3_O_4_ control the stabilization of intermediates and influence performance over time, thereby enhancing the understanding of the factors that affect catalyst activity.

## 2 Experimental Section

### 2.1 Physical Characterization

X-ray diffraction (XRD) analysis was carried out in a Bruker, D8 Advanced Eco diffractometer from 2θ = 10° to 140°. A Cu source (*k*_α_ = 8 keV, *Q*_MAX_ = 8 Å^–1^) was used as an X-ray source and the data were collected in Bragg–Brentano geometry. To remove cobalt fluorescence radiation from the Cu-excitation, a LYNXEYE XE detector was used. X-ray photoelectron spectroscopy (XPS) spectra were collected in a Thermo Scientific K-Alpha spectrometer (Al Kα gun source, 300 μm spot size, 50.0 eV pass energy, and 0.100 eV step size). XPS and XRD data were collected from drop-casted Co_3_O_4_ nanoplates onto an Au-foil. In situ X-ray absorption near edge spectroscopy (XANES) analysis was performed at Carnaúba/Sirius beamline, at the Tarumã station, using a nanoprobe (beam size 200 nm × 500 nm, estimated flux ∼10^9^ photons/second on the sample) and 4-bounce Si(111) monochromator (10^–4^ energy resolution at 0.5 eV step) in fluorescence mode. X-ray fluorescence (XRF) maps were acquired in continuous scanning (flyscan) mode over 20 × 20 μm^2^ area, with a lateral step of 400 nm by raster-scanning the sample relative to the stationary beam.^56^ Spectra were collected in chronoamperometry (CA) in 1.0 mol L^–1^ + 20 mmol L^–1^ NaNO_3,_ initially at the open circuit voltage (OCP), and from 0.0 to −0.5 V vs RHE at 100 mV step controlled by a EC301 Stanford Research System workstation. Principal component analysis (PCA) was applied to reconstruct pixel-resolved spectra from the stack of XRF images collected across the Co K-edge XANES data set.^57^ Data alignment and XAS spectra extraction were performed using the ATHENA software.^58^ Scanning electron microscopy (SEM) and high-resolution transmission microscopy (HRTEM) images were acquired in a FEI Quanta FEG 250 (at 20 kV accelerating voltage) and a JEOL JEM-2010 (at 200 kV accelerating voltage) microscopes. All micrographs were treated using ImageJ software.

### 2.2 Co_3_O_4_ Nanoplates Synthesis

All chemicals used are commercially available and were used without further purification. In a typical synthesis, 0.285 g of CoCl_2_·6H_2_O (Sigma-Aldrich, 98–99%) was dissolved in 10 mL of Milli-Q water (18.2 MΩ cm), and then 3 mL of triethylamine (TEA, Sigma-Aldrich, 99%) was added under vigorous stirring. After 5 min, the mixture was transferred to a Teflon vessel (occupying 80% of the volume), which was then placed inside a stainless-steel autoclave and heated in an oven at 160 °C for 24 h. After achieving room temperature, the resulting products were collected by centrifugation and washed several times with ultrapure water (until negative for chloride test with silver nitrate). Then, the precipitate was washed and centrifuged with absolute ethanol (Ciclo Farma, 99.5%), followed by vacuum drying at 60 °C for 6 h. The obtained Co­(OH)_2_ was converted to Co_3_O_4_ nanoplates (Co_3_O_4_-np) by thermal topotactic conversion (1 h at 350 °C).

### 2.3 Preparing the Working Electrode

The electrode was prepared as follows: 5 mg of Co_3_O_4_-np was dispersed in 480 μL of isopropanol and 20 μL of Nafion 117 (Sigma-Aldrich, 5% m/V in aliphatic alcohols and water). Then, the mixture was suspended in an ultrasonic bath for 10 min and stored in a fridge for later use. Before running the experiment, the catalyst ink was bath-ultrasonicated for 1 h and two 5 μL aliquots drop-casted onto a glassy carbon substrate (0.25 cm radius, geometric area of 0.196 cm^2^, estimated mass/area ratio of 0.51 mg cm^–2^) successfully making the Co_3_O_4_-np/GC modified electrode. The first drop was dried before the second addition. For each electrochemical experiment, the substrate was cleansed with isopropanol and polished with silica suspension.

### 2.4 Electrochemical Workstation

All electrolysis experiments were carried out at room temperature in a H-type electrochemical cell consisting of three electrodes. A carbon rod served as the counter electrode, a RHE as the reference electrode, and Co_3_O_4_-np drop-casted on glassy carbon as the working electrode (WE). Both the anode and cathode compartments were separated by a Fumasep FAB-PK-130 anionic membrane. The catholyte was made of NaOH (Sigma-Aldrich, 99.6%) 1.0 mol L^–1^ and NaNO_2_ (Sigma-Aldrich, 99.5%) or NaNO_3_ (Sigma-Aldrich, 99.5%) 20 mmol L^–1^ whereas the anolyte only contained NaOH 1.0 mol L^–1^. The solutions were prepared with ultrapure water Milli-Q (18.2 MΩ cm). Before any analysis, the working compartment’s solution was purged with high-purity argon (Ar, 99.999%, PraxAir) for at least 30 min and kept the headspace Ar-saturated during the experiments. The electrochemical cell was controlled by a PGSTAT302N equipped with FRA32M and SCAN250 modules. Prior to each electrochemical experiment, electrochemical impedance spectroscopy (EIS) was performed to determine the solution resistance (*R*_S_) from 100 kHz to 100 mHz at OCP and 10 mV_RMS_ as bias voltage. Additional EIS measurements were conducted at the steady state under chronoamperometric conditions from 0.0 to −0.5 V vs RHE. Cyclic voltammograms were obtained at 20 mV s^–1^ from 0.1 to −0.6 V vs RHE and normalized by the electrochemical surface area (ECSA), determined by the double-layer capacitance. 1 h electrolysis (from 0.0 to −0.5 V vs RHE) were conducted in nitrate and nitrite. The applied potential was corrected during the measurements with *iR* compensation (85%).

### 2.5 DRT Analysis

The final EIS spectra acquired from 0.0 to −0.5 V vs RHE were further analyzed by deconvolving them into their corresponding DRT. Real (*Z*′) and imaginary (*Z*″) components of the impedance (Z) were discretized using radial basis functions and regularized via the Tikhonov regression method.^59^ The regularization hyperparameter λ was optimized individually for each *Z* data set via Re-Im-cross-validation,^60^ with λ sampled from the geometric series 10^2^, 10^1^, ..., 10^–5^, and 10^–6^. The contribution of each electrochemical process to *Z* was quantified by integrating the area under its respective DRT band. To visualize the evolution of dynamic processes across faradaic potentials, a two-dimensional DRT heatmap was constructed using the Julia package KernelInterpolation.jl.^61^ The analysis covered a potential range from 0.0 to −0.5 V vs RHE. A two-dimensional Gaussian kernel with shape parameter 1.0 was applied over a regular 150 × 150-point grid to perform the interpolation.

### 2.6 Ammonia and Nitrite Quantification

NH_3_ was quantified using the standard addition protocol based on the indophenol method. Initially, 10 ppm of NH_3_ standard solution was prepared from ammonium chloride (NH_4_Cl, Sigma-Aldrich, 99.5%) dissolved in ultrapure Milli-Q water. Then, the oxidant solution was prepared by dissolving 0.41 g of sodium citrate dibasic trihydrate (Sigma-Aldrich, 99.5%) and 1.0 mol L^–1^ sodium hydroxide (NaOH) (Sigma-Aldrich, 99.6%) in 20 mL of water, followed by the addition of 5 mL of a 5% (w/v) aqueous sodium hypochlorite (NaOCl) (Sigma-Aldrich, 99.5%) solution. Additionally, a solution of 1.0 mol L^–1^ salicylic acid (Sigma-Aldrich, 99.5%) and NaOH was prepared in 100 mL. For the analytical procedure, 1 mL of postelectrolysis catholyte was added to five separate 10 mL volumetric flasks. Subsequently, varying volumes (from 0.0 to 1.0 mL) of the 10 ppm of NH_3_ standard, 400 μL of the oxidant solution, 160 μL of 0.5% (w/v) aqueous sodium nitroprusside solution, and 160 μL of the salicylate solution were added to each flask. The flasks were then filled up to 10 mL of water. After mixing, the solutions were allowed to rest for 1 h. Measurements were then performed using a BEL Engineering UV-M51 UV–vis spectrophotometer, scanning from 500 to 800 nm with a step size of 1 nm. The NH_3_ concentration for each sample was determined from the analytical curves measured at 652 nm, exhibiting excellent correlation coefficients (Figure S1). The analyte concentration was calculated by extrapolating the curve intersection with the *x*-axis. Additionally, quantitative nuclear magnetic resonance was also employed to validate the quantification (Figure S2). No additional sources of NH_3_ were detected.

Nitrite, on the other hand, was quantified by the Griess method.^62^ A variable amount of the catholyte aliquot was diluted in 1 mL of water. Later, 1 mL of the chromogenic solution, which consisted of 0.1 g of *N*-(1-naphthyl)­ethylenediamine (C_12_H_16_Cl_2_N_2_, Sigma-Aldrich, 99%), 1.0 g of sulfanilamide (H_2_NC_6_H_4_SO_2_NH_2_, Sigma-Aldrich, 99.6%), and 10 mL of phosphoric acid (H_3_PO_4_, Sigma-Aldrich, ≥85%) in 100 mL of water) was added to the solution, and the final volume completed to 4 mL with water. The UV–vis spectra were acquired from 400 to 650 nm, and the maximum at 543 nm was used for quantification based on the analytical curve (Figure S3).

### 2.7 Electrochemical Mass Spectrometry

Electrochemical mass spectrometry (EC-MS) experiments consisted of running both chronoamperometric and voltammetric measurements coupled to a mass spectrometer, where the following volatile species were tracked by their ionic currents, with the following mass/charges (*m*/*z*): 2 (H_2_^+^), 17 (NH_3_^+^), 28 (N_2_^+^), 30 (NO^+^), 33 (NH_2_OH^+^), and 46 (NO_2_^+^). The analysis proceeded using an OmniStar GSD (Pfeiffer Vacuum) gas analyzer, equipped with a heated stainless-steel capillary probe (0.125 mm inner diameter). The WE consisted of 30 μL of the catalytic ink drop-casted onto a disk-shaped carbon paper (E-TEK, 50% wet-proof, 110 μm thickness and 10 mm diameter), pressed previously against a PTFE membrane (Gore-Tex, 0.02 μm pore size and 50 μm thickness) by applying 1.0 ton/cm^2^ for 1 min. An Ag|AgCl (sat. KCl) and a graphite rod were used as reference and counter electrodes, respectively. The area of the WE exposed to the electrolyte was 0.38 cm^2^. The experiments were conducted in Ar-saturated 1.0 mol L^–1^ NaOH and 20 mmol L^–1^ NaNO_3_.

### 2.8 Gas Chromatography

Hydrogen gas (H_2_) generated during constant-potential electrolysis was quantified by using an Agilent 8860 gas chromatograph equipped with a thermal conductivity detector (TCD). A two-compartment gastight H-type cell separated by an anionic membrane was used in the experiments. Except for the gastight configuration of the H-type cell, all electrodes, electrolytes, and preparation methods were the same as those previously described for the regular H-type cell. A digital mass flow controller (AALBORG, GFC17) was used to deliver argon gas at a flow rate of 10 mL min^–1^ through the electrochemical cell during electrolysis, and the outlet gas was collected in a 1.0 L commercial sampling bag (Tedlar PLV gas sampling bags). Using a gastight syringe (Hamilton, 25 mL), samples of the collected gas were injected into the GC. The quantification was performed by using a calibration curve constructed from known dilutions of a standard gas mixture (3% H_2_ in argon).

## 3 Results and Discussion

### 3.1 Synthesis and Characterization of Co_3_O_4_ Nanoplates

Co_3_O_4_-np were obtained by hydrothermal treatment of CoCl_2_·6H_2_O using triethylamine (TEA) as the ligand, followed by calcination. We confirmed the hexagonal morphology (Figure 1A–C) with an average length of 189 ± 38 nm and width of 37 ± 12 nm (Figure S4) as measured by SEM (from the counting of 100 particles). TEM images show the hexagonal morphology with uneven mass thickness and diffraction contrast, indicating a polycrystalline structure, Figure 1B. An HRTEM micrograph corresponding to the top view (Figure 1C) of Co_3_O_4_-np is presented, showing that nanoparticles are polycrystalline, as seen by the different crystallographic orientation and the grain boundaries observed on a single nanoparticle. Several crystallographic orientations are observed in the Fast Fourier Transform image in the inset of Figure 1C and the (113) plane was indexed to the Co_3_O_4_ phase. Also, an HRTEM image of the side-view is presented showing the platelet morphology with crystallographic distance of 4.6 Å, indicating the [111] direction of Co_3_O_4_, Figure 1D. Compared to Co­(OH)_2_-np micrographs (Figure S5), the variations in contrast correspond to superficial defects originated from the calcination step and the polycrystalline structure, leading to a different diffraction contrast. It is also possible to see hexagons stacked on top of each other (Figure 1D) due to the nanoplates ultrathin character. Figure 1E highlights the Co_3_O_4_-np XPS profile. The Co^3+^ component is assigned to a lower binding energy (779.4 eV) than Co^2+^ components (780.9 and 782.4 eV) due to several factors such as final state relaxation, covalent interactions and screening effects, variations in local bonding and lattice effects.^63,64^ Additionally, the Co^2+^ component presents higher broadness than Co^3+^ due to j–j coupling multiplet splitting,^64^ being further deconvoluted into two additional bands, as proposed by Biesinger et al.^63^ The relative area contributions of the deconvoluted bands were determined as Co^3+^ (41.7%), Co^2+^ (37.4%), satellite 1 (13.9%), and satellite 2 (7.0%), revealing a superficial cation ratio of approximately 1:1.

**Figure 1:**
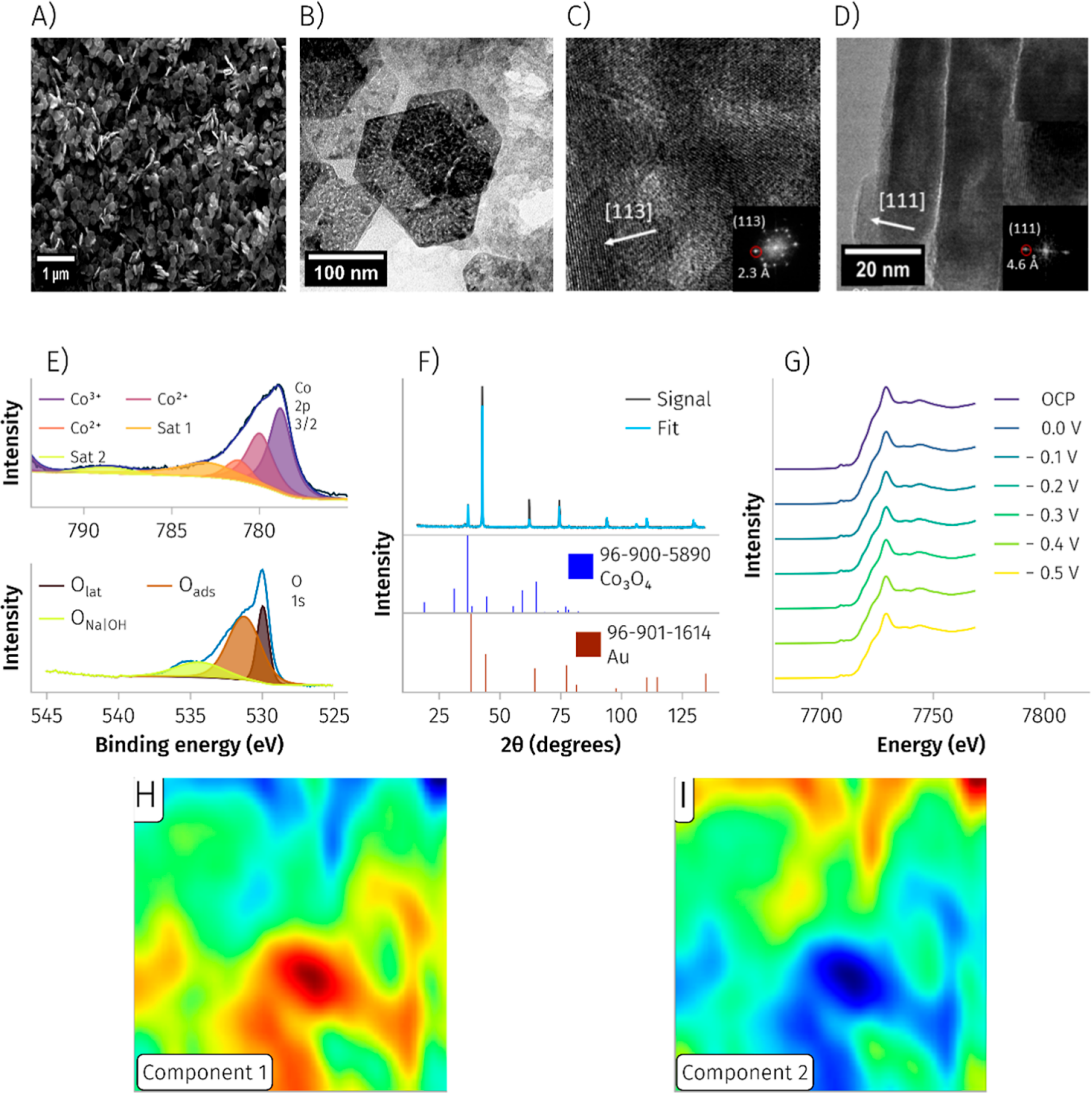
Physical characterization of pristine Co_3_O_4_-np. (A) SEM image of Co_3_O_4_-np on an Au plate substrate. (B) TEM images of Co_3_O_4_-np along with their HRTEM images of Co_3_O_4_-np from the (C) top-view and (D) side-view, respectively. (E) Co 2p and O 1s XPS spectra of Co_3_O_4_-np/Au. (F) XRD diffractogram of Au and Co_3_O_4_ patterns and Co_3_O_4_-np deposited on an Au substrate. (G) In situ XANES spectra of Co_3_O_4_-np under different applied potentials. (H,I) In situ spatially resolved distribution of Co_3_O_4_-np/GC components through a stack 20 × 20 μm^2^ of synchrotron X-ray fluorescence (SXRF) images acquired over Co_3_O_4_ K-edge. Measurements taken at OCP.

Both Co 2p_3/2_ and 2p_1/2_ bands are deconvoluted in Figure S6. Co^2+^ sites are primarily responsible for the generation of strong satellite peaks, whereas Co^3+^ sites contribute less significantly, which corroborates that the surface of Co_3_O_4_-np samples are Co_3_O_4_.^64^ Regarding the oxygen 1s window, the bands have been assigned as O_lat_ to lattice oxygen (530.0 eV), O_ads_ (531.3 eV) to O adsorbed species and defect-related oxygen from the oxide structure, and O_Na/OH_ (534.4 eV) for both the Na Auger emission associated with the Nafion 117 binder used in the catalyst ink and chemisorbed OH-like species.^65,66^ Ex situ XPS measurements were conducted on different electrolysis conditions and the correlations between bands were evaluated by PCA algorithm, as proposed by Mc Evoy et al. and others (Figure S7).^67,68^ A positive correlation was found between Co 2p, O_lat_, and O_ads_ bands, whereas no correlation was observed with the Na band, supporting the proposed band assignments (Figure S8). The relative areas of the oxygen bands were O_lat_ (24.1%), O_ads_ (56.4%), and O_Na/OH_ (19.5%). The XRD diffractogram (Figure 1F) was refined according to Rietveld’s method,^69^ reaching agreement indexes of *R*_W_ = 9.87% and GOF = 3.27 for the Co_3_O_4_ JCPDS card no. 96-900-5890. The main observed peaks are the ones from Au substrate and the Co_3_O_4_ information was obtained by the refinement. It displays a cubic *Fd*3̅*m* phase with a zone axis [001], and the refined lattice parameters are *a* = *b* = *c* = 8.127 Å and α = β = γ = 90°, a cell volume of 536.78 Å^3^, and a cell density of 5.96 g cm^–3^, values consistent with spinel Co_3_O_4_ phase.^70^ The diffraction planes (111) and (113) found from HRTEM are consistent with XRD data, confirming that the Co_3_O_4_-np sample is the cubic phase of cobalt oxide.

Figure 1G shows in situ XANES measurements at different applied potentials, starting at OCP and then under NO_3_RR condition, from 0.0 to −0.5 V vs RHE in 1.0 mol L^–1^ NaOH and 20 mmol L^–1^ NaNO_3_. Two characteristic features of the Co_3_O_4_ spinel phase were identified, the pre-edge at 7709.0 eV and the white line at 7720.5 eV, corresponding to the 1s to 3d transition and the dipole allowed 1s to 4p transition to empty final states, respectively.^71^ In order to spatially map other compositional phases to the WE (Co_3_O_4_-np/GC), in situ spatially resolved Co K-edge XANES acquired at OCP, Figure 1H,I, was employed over a 20 × 20 μm^2^ area under NO_3_RR conditions. The region of interest was selected based on in situ XRF distributions of Co signals across the WE surface. Finally, PCA was applied to reconstruct the XANES spectra at each pixel from the XRF stack measured around the Co–K edge, identifying two distinct spectral components using the ITFA algorithm, denoted Component 1 and Component 2, which were identified and closely match the Co_3_O_4_ and CoOOH standard samples, respectively (Figure S9). Spatial mapping reveals that Component 1 is the dominant component across the area analyzed. Although bulk CoOOH formation is thermodynamically expected only at potentials preceding the OER, according to the Pourbaix diagram,^72^ its occurrence has been reported outside this stability region.^73–75^ Here, Component 2 is attributed to a surface CoOOH-like layer formed on Co_3_O_4_ particles under alkaline conditions. This interpretation is further supported by regions of intermediate intensity (green), where concurrent contributions from both phases indicate the spatial coexistence of mixed-phase domains. Notably, Component 2 exhibits a slightly lower oxidation state than bulk CoOOH, as evidenced by the inset of Figure S9, suggesting an average Co-oxidation state below +3.

### 3.2 NO_3_RR Performance

Figure 2A shows the 10th scan of 3 sets of cyclic voltammetry (CV) experiments in different supporting electrolytes. In alkaline medium (nitrate and nitrite-free electrolyte), a negligible current density was observed (green line). In the presence of NO_3_^–^ (orange line), however, it is clearly seen an onset potential at around −0.3 V vs RHE, followed by an exponential-like increase of current due to concomitant nitrate reduction with HER. The blue line shows an onset potential around 0.0 V vs RHE, with the formation of a sigmoid-like current profile, up to around −0.3 V vs RHE, where a hysteresis occurs. From this point onward, the current density increases abruptly. All cases developed bubbles at more negative potentials, further indicating the occurrence of HER as a side reaction. Electrochemically active surface area (0.154 cm^2^) was determined by measuring the double-layer capacitance. Figure 2B depicts the capacitive CV in a nonfaradaic region (±50 mV around the OCP) at different scan rates (from 10 to 100 mV s^–1^). Figure 2C shows the potential-dependent Faradaic efficiencies (FE) to NH_3_ from NO_2_RR and NO_3_RR with [NO_*x*_^–^] = 20 mmol L^–1^. The NH_3_ was generated during 1 h CA experiments with potentials ranging from 0.0 to −0.5 V vs RHE at a 100 mV step. The highest efficiency achieved was 92.5 ± 7.5% at –0.2 V vs RHE in the presence of NO_2_^–^, corresponding to the sigmoidal profile peak position in the cyclic voltammogram, suggesting that this region relates mostly to NO_2_RR.

**Figure 2:**
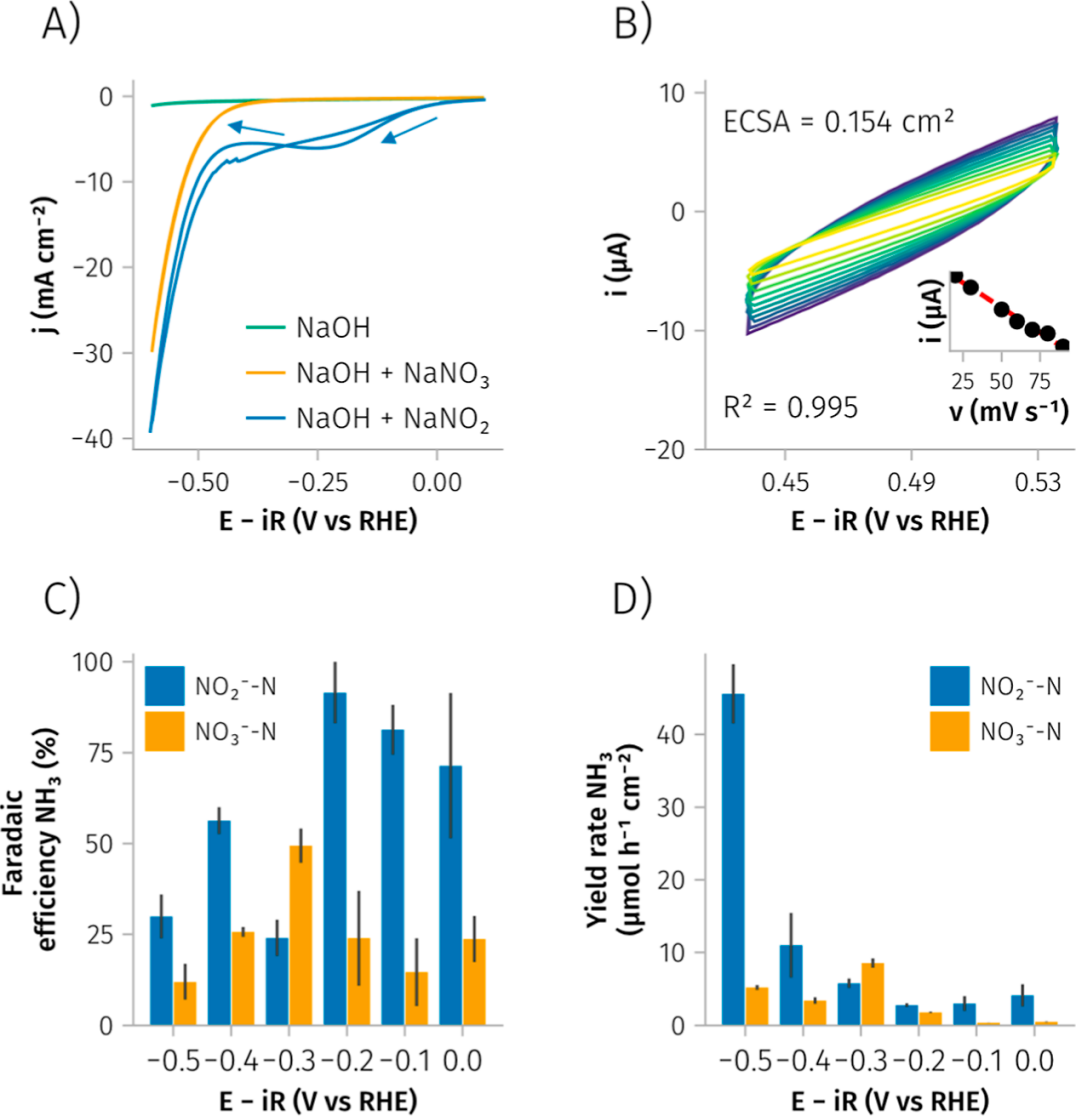
Electrochemical characterization. (A) Cyclic voltammogram of Co_3_O_4_-np/GC under different supporting electrolytes (20 mmol L^–1^ NO_2_^–^ or NO_3_^–^) in 1.0 mol L^–1^ NaOH from 0.1 to −0.6 V vs RHE at a 20 mV s^–1^ scan rate, with 85% *iR*-drop compensated. (B) Capacitance measurements taken at ±50 mV around OCP with different scan rates (10 to 100 mV s^–1^) and linear regression *y* = −1.29 × 10^–6^ – 3.30 × 10^–8^·*x*. (C,D) FE and YR for NH_3_ from NO_2_RR (blue) and NO_3_RR (orange), respectively, obtained by 1 h CA from 0.0 to −0.5 V vs RHE (85% *iR* compensated) in 1.0 mol L^–1^ NaOH and 20 mmol L^–1^ NO_*x*_^–^.

The obtained NH_3_ YRs (Figure 2D) were calculated in the μmol h^–1^ cm^–2^ (normalized by ECSA) magnitude for all tested conditions. The best performance was achieved for the reduction of NO_2_^–^ at −0.5 V vs RHE with a yield value of 45.6 ± 4.1 μmol h^–1^ cm^–2^. For NO_2_RR, the YR increased as long as the potential got more negative, whereas for NO_3_RR the yield remained approximately similar (∼3.31 μmol h^–1^ cm^–2^ on average). These findings indicate that Co_3_O_4_ exceeds in converting NO_2_^–^, but still lacks activity for NO_3_^–^ reduction, which is consistent with the literature proposal that NO_3_^–^ to NO_2_^–^ reduction is the rate-determining step for NO_3_RR.^76–78^ This value is highly dependent on system parameters such as pH, electrolyte concentration, type of cell, etc., but it is still lower than the current state-of-the-art for both NO_3_RR and NO_2_RR (∼mmol h^–1^ cm^–2^).^24,79^ Liu et al. anchored Cu single atoms on N-doped carbon with Co_3_O_4_ nanosheets, attaining FE of 97.7% at −0.8 V vs RHE and YR of 6.7 mmol h^–1^ cm^–2^ at −1.0 V vs RHE in 1.0 mol L^–1^ KOH and KNO_3_.^78^ Fan et al., on the other hand, synthesized a self-supported carbon nanosheet-based CoP electrocatalyst for NO_3_RR, reaching FE of 93.3% and YR of 8.47 mmol h^–1^ cm^–2^ at −0.33 and −1.03 V vs RHE, respectively, under chronoamperometric conditions in 1.0 mol L^–1^ NaOH and NaNO_3_.^80^ To the best of our knowledge, these represent the highest efficiencies reported to date for NO_3_RR, with most other catalysts achieving YRs in the μmol h^–1^cm^–2^ range.^81^ A performance comparison with literature-reported catalysts is summarized in Table S1. These results emphasize the need for further modifications to Co_3_O_4_-np to enhance its reaction kinetics.

### 3.3 Accelerated Postaging Performance

It is a common practice to subject the electrocatalyst to chronoamperometric polarization prior to NH_3_ production. Fan et al. polarized a CoP electrocatalyst under a constant potential before running the NO_3_RR in order to reach the steady state.^80^ Similarly, Costa et al. subjected a Cu/Cu_2_O composite under a prereduction step before evaluating the kinetics for NO_3_RR, which was done to map possible active sites, as this treatment improved the catalyst performance for the reaction, which the authors attributed to the increase of oxygen-related defects.^82^ However, our previous characterization revealed no significant structural and electronic changes to Co_3_O_4_-np/GC following chronoamperometric polarization (Figure 1G). Kani et al. reported the performance of Co bulk for NO_3_RR, where the catalyst was submitted to CV in order to form oxide-derived Co, leading to a 4-fold increase in activity for the reaction.^83^ Similarly, He et al., aiming for the reconstruction of Co–Cr spinel oxides for OER, applied 1000 cycles of CV under OER conditions, in order to correlate surface states with both catalyst’s activity and stability.^84^ Following this line of thought, a more rigorous potential treatment was done to pristine Co_3_O_4_-np/GC, where the sample was modified also across 1000 voltammetric cycles, under continuous stirring at 800 rpm, using the same potential window and scan rate as before (0.1 to −0.6 V vs RHE, 20 mV s^–1^), in the presence of NaOH 1 mol L^–1^ and NaNO_3_ 20 mmol L^–1^. This procedure mimics an electrochemical accelerated aging process, which could happen under common operating conditions for NH_3_ electrosynthesis.^85,86^ From now on, we will classify our postcycled catalyst as an aged one. Under this regime, the onset potential shifted positively to −0.1 V vs RHE (Figure 3A) with the current density increasing steadily up to the 750th cycle. Post-treatment performance (aged Co_3_O_4_-np/GC) is shown in Figure 3B,C, exhibiting an average FE decrease of 18.8%, and an average YR increase of 9.10 μmol h^–1^ cm^–2^ compared to the pristine catalyst. Both average values are computed across all quantified potentials. No detectable NH_3_ production was observed at 0.0 V vs RHE, suggesting a shift in the overpotential required for NO_3_RR. Additionally, for NO_2_RR, FE declined after cycling as well. However, at −0.4 V vs RHE and above, the YR values increased by an average of 11.44 μmol h^–1^ cm^–2^ across all quantified potentials compared to untreated Co_3_O_4_-np/GC.

**Figure 3:**
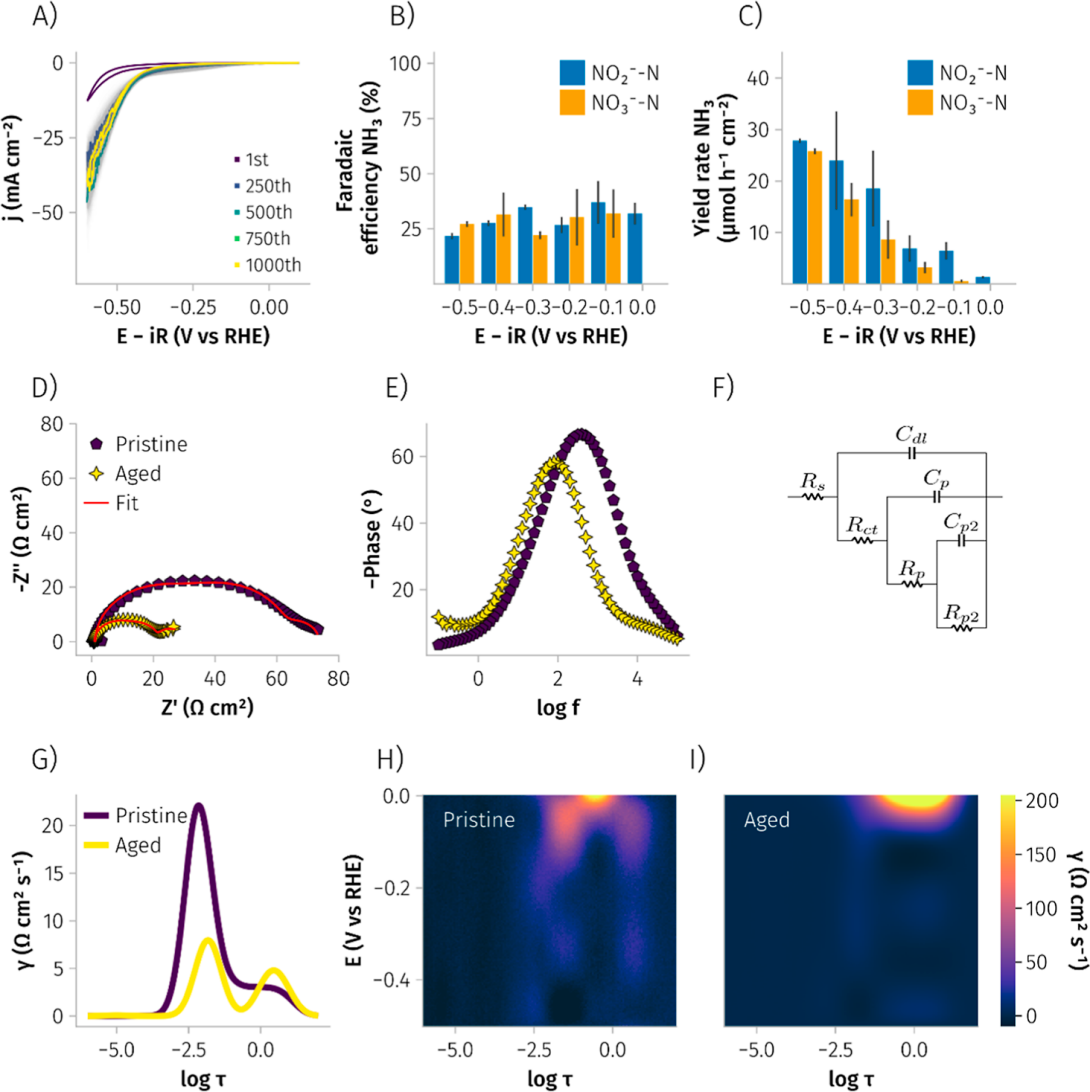
Aging evaluation of Co_3_O_4_-np/GC. (A) 1000 cycles of CV in the presence of 1.0 mol L^–1^ NaOH and 20 mmol L^–1^ NaNO_3_ from 0.1 to −0.6 V vs RHE at a 20 mV s^–1^ scan rate with 85% *iR* compensation. (B,C) FE and NH_3_ YR, respectively, under different supporting electrolytes measured after 1 h CAs from 0.0 to −0.5 V vs RHE (85% *iR* compensated). (D,E) EIS spectra collected from 100 kHz to 100 mHz with *V*_RMS_ of 10 mV at –0.2 V vs RHE (Nyquist and Bode, respectively). (F) Equivalent electric circuit used to fit the EIS data. *R*_S_ refers to the electrolyte resistance, *R*_CT_ is the charge-transfer resistance, and *R*_P_ accounts for polarization resistances due to intermediates adsorptions. *C*_DL_ is the electric double layer capacitance and *C*_P_ are polarization capacitances. (G) DRT obtained at −0.2 V vs RHE. (H,I) heatmaps of DRT before and after aging, respectively, with EIS measurements taken from 0.0 to −0.5 V vs RHE as well.

Both EIS spectra of pristine and aged Co_3_O_4_-np/GC taken at −0.2 V vs RHE are displayed in Figure 3D (Nyquist) and 3E (Bode diagram). In the former, three semicircles are assigned for both catalysts, with their radius decreasing, suggesting a lower charge-transfer resistance (*R*_CT_). The first semicircle can be attributed to one process being shifted below the *Z*′ axis due to surface inhomogeneities or due to the convolution of more processes. The Bode phase supports the latter interpretation, as the asymmetrical shape of the purple bell-shaped curve indicates overlapping processes. In particular, aged Co_3_O_4_-np/GC also shows suppression of the band at 10^4^ Hz, leaving the first band resolved at 10^2^ Hz. Figure 3F shows an equivalent circuit derived from previously proposed NO_3_RR mechanisms, where both NO_3_^–^ and H-species are treated as adsorbates, with the H adsorption proceeding via a Volmer step, and the NO_3_^–^ to NO_2_^–^ conversion being the rate-determining step (more details in the Supporting Information).^8,22,52,87,88^ In this model, the semicircles on Nyquist have contributions from intermediate adsorption steps. The charge-transfer resistance is related to all steps involving electron transfer (Faradaic currents).^89^ The polarization resistances (*R*_P_) are associated with steady-state current, relating to the activation energy required for an adsorbate to bind to the WE surface.^89,90^ The polarization capacitors (*C*_P_) store energy in the form of chemical bonds/adsorptions while delaying the phase of the AC signal.^90^ Following activation, *R*_CT_ decreased with the aging from 4.01 × 10^1^ to 1.50 × 10^1^ Ω cm^2^ at −0.2 V vs RHE, as well as the polarization resistances Rp_1_ and Rp_2_ from 2.29 × 10^1^ Ω cm^2^ and 9.38 Ω cm^2^ to 6.13 Ω cm^2^ and 8.18 Ω cm^2^ at −0.2 V vs RHE, respectively. These values suggest that the aging improved both the adsorption of species toward the WE surface and their electron transfer kinetics as well (the circuit fitting parameters across the potentials are shown in Figure S10, Table S2 and S3), although selectivity for ammonia was most likely affected by HER and/or N-containing species production. Still, a view of the electrode polarization processes across all measured Faradaic potentials is required.

Figure 3G presents the DRT spectrum recorded at −0.2 V vs RHE for both pristine and aged Co_3_O_4_-np/GC. Unlike model-based EIS, which requires prior knowledge to diagnose an electrochemical system,^91^ time scale-based DRT directly identifies the characteristic relaxation times of electrochemical polarizations through feature analysis, including position, area, and height.^92^ Two distinct bands are observed for each sample. The bands at shorter relaxation times (τ) (7.08 × 10^–3^ s for pristine and 1.51 × 10^–2^ s for aged) are assigned to charge-transfer (CT) processes, while longer τ′s (2.04 and 2.82 s) correspond to mass transport (MT) polarizations.^93^ In both cases, the CT bands exhibit the largest area, implying that the NO_3_RR is primarily limited by electron transfers at this potential, whereas the aged sample presents both bands with lowest intensities. For pristine Co_3_O_4_-np/GC, CT accounts for 73% of the total impedance, compared to 59% in the aged sample. Notably, aging leads to 65% decrease in CT resistance, supporting enhanced reaction kinetics. Figure 3H,I shows the DRT profiles from 0.0 to −0.5 V vs RHE for both catalysts. The resulting heatmap displays the DRT intensity γ­(τ) as a function of both the logarithm of the relaxation time log_10_(τ) and the applied potential. This representation enables a clear visualization of how relaxation processes shift, emerge, or decay as the system progresses toward more negative potentials. The CT processes shifted toward lower τ′s, implying the active species reached the active site faster (i.e., the process relaxes faster), with a steady decline in area, suggesting a lowering in the process activation barrier as the potential becomes more negative.^93^ The MT case mainly shows a higher dispersion of time constants for the pristine catalyst (Figure 3G), with the aging treatment regularizing them into a narrower band.

Specifically, pristine Co_3_O_4_-np/GC CT relaxation time decreased from 1.53 × 10^–2^ (0.0 V vs RHE) to 5.01 × 10^–4^ s (−0.5 V vs RHE), whereas in the postcycled catalyst it drops from 1.0 × 10^–2^ to 3.38 × 10^–4^ s, further confirming that the aging increased the CT kinetics. Additionally, aged Co_3_O_4_-np/GC DRT taken at 0.0 V vs RHE reveals an additional band within the MT regime, resulting in a total MT-related impedance up to 5-fold higher than that of the pristine catalyst (Figure S11). This increased MT resistance aligns with the absence of NH_3_ yield at 0.0 V vs RHE for aged Co_3_O_4_-np/GC, as the hindered transport limits the delivery of reactive species to the WE surface, contributing to the observed positive shift in the overpotential. Therefore, the aging effect on the YR is attributed to improved electron-transfer kinetics postcycling. However, the concurrent decrease in FE implies lower selectivity, which cannot be explained by electrochemical data alone. Instead, this behavior likely stems from structural/compositional changes that arise from the voltammetric treatment, highlighting the need for further physical characterization of the aged Co_3_O_4_-np.

### 3.4 Postaging Electrocatalyst Characterization

Other works evaluated the stability of Co_3_O_4_ for NO_3_RR during long CA experiments at the highest FE potential,^94^ and by replenishing the electrolyte during multiple chronoamperometric cycles.^94,95^ However, the catalyst physical structure has yet to be characterized after long-duration treatments. Here, we performed the same set of ex situ and in situ characterizations of pristine Co_3_O_4_-np to the electrocatalyst after cycling. Figure 4A presents the SEM micrography of the postaged sample. While the overall morphology of the nanoplates remained unchanged, the size distribution reveals a small increase in length to 199 ± 43 nm (∼9% increase), with no significant change in width (35 ± 6 nm, Figure S12). Figure 4B shows a top-view HRTEM image of the aged sample, revealing the presence of an amorphous phase among the hexagonal nanostructures and on their surface. The lateral view (Figure 4C) further emphasizes edge degradation, with signs of amorphization following voltammetric cycling. Additionally, the absence of well-defined interplanar spacing at the edges supports the notion that NO_3_RR predominantly occur at these regions, owing to their higher reactivity arising from undercoordinated atoms and lattice strains.^96^

**Figure 4:**
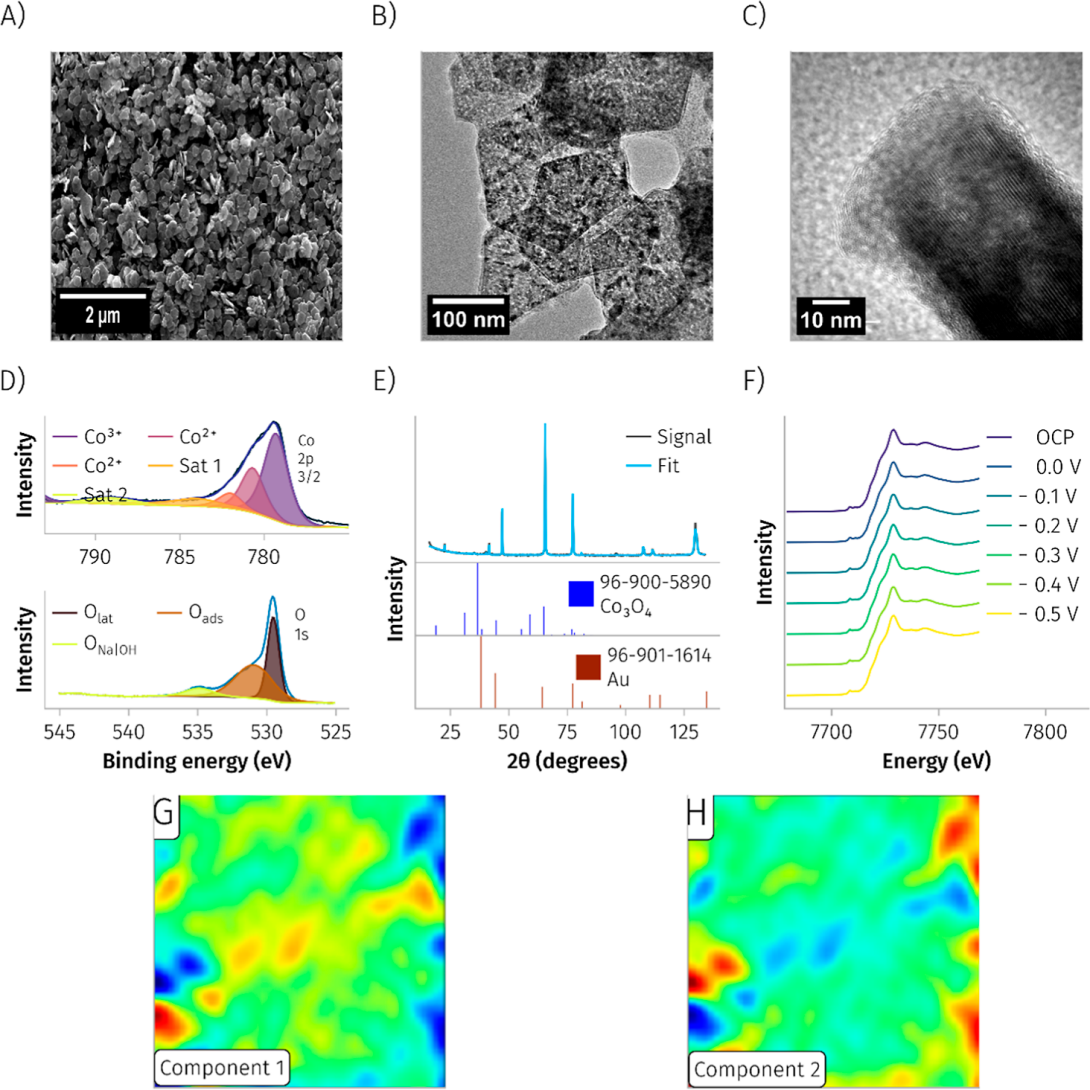
Aged Co_3_O_4_-np physical characterizations. (A) SEM image of the Co_3_O_4_-np on Au plate substrate. (B,C) HRTEM images of Co_3_O_4_-np looked from above and sideways, respectively. (D) XPS spectra of Co_3_O_4_-np drop-casted on an Au plate. (E) XRD diffractogram of Au and Co_3_O_4_ patterns and Co_3_O_4_-np deposited on an Au substrate. (F) In situ XANES spectra of Co_3_O_4_-np under different applied potentials Measurements taken at OCP. (G,H) In situ spatially resolved distribution of Co_3_O_4_-np/GC components through a stack 20 × 20 μm^2^ of SXRF images acquired over Co_3_O_4_ K-edge. Measurements taken at OCP.

The rising of defects and amorphization accompanied an increased NH_3_ production (Figure 3C), which we previously attributed to the lowering of charge-transfer resistances (Figure 3D), implying better CT kinetics as confirmed by the DRT maps (Figure 3H,I), and the lowering of the MT energy barrier. However, the selectivity for NH_3_ diminished postcycling (Figure 3B), coinciding with the shift in overpotential at which NO_3_RR proceeds (Figure 3A). A comparable effect is observed for Cu-based NO_3_RR, where Messias et al. evaluated the morphological changes via ex situ microscopies and structural modifications of well-defined Cu_2_O nanocubes during NO_3_RR by in situ XANES, reporting the catalyst reduction to metallic copper during electrolysis, which was associated with the increase of FE to NH_3_ compared to the pristine catalyst.^27^ Similarly, Yoon et al. tracked this sample transformation via operando electrochemical liquid cell transmission electron microscopy (EC-TEM), linking time-resolved morphological evolution to the exposure of different active sites.^97^ The authors concluded that the Cu_2_O restructuring significantly alters both the reaction pathway and product distribution. A key distinction, however, is that for Cu-based NO_3_RR, chronoamperometric treatments at potentials with high FE are sufficient to induce both structural and morphological changes in the sample. In contrast, a consecutive potential cycle treatment was employed for Co_3_O_4_-np in order to achieve morphological restructuration. This new condition improved the NH_3_ formation route by increasing YR up to 5-fold at −0.4 V vs RHE. But it also enhanced other byproducts pathways as well, as supported by the FE for NH_3_ decreasing to 23.8% on average.

To gain a better understanding of the mechanism of NO_3_RR mediated by the Co_3_O_4_-np catalyst, electrochemical mass spectrometry (EC-MS) analysis was conducted for both pristine and aged Co_3_O_4_-np, with the produced fragments monitored across CV and CA protocols (Figures S13 and S14, respectively). No additional fragments were detected after the aging treatment, but the ionic currents reached different intensities, suggesting an alteration of the product distribution. In particular, aging appears to slightly increase the N_2_^+^ signal, whereas H_2_^+^ shows a more pronounced increase across the explored potential windows. Assays to quantify both H_2_ and N_2_ were performed using GC; however, the N_2_ produced could not be distinguished from the small amount introduced from ambient air within the needle dead volume. This contamination happens during gastight syringe sampling and injection. The chromatograms of the TCD signal are displayed in Figure S15.

Figure 4D displays the aged Co_3_O_4_-np XPS spectra. The Co 2p window reveals the same Co_3_O_4_ spinel profile as shown for the pristine sample (Figure 1E), but with an increase in the Co^3+^ component’s relative area (from 41.7% to 59.0%) and corresponding decrease in the Co^2+^ contribution (from a total of 37.44% to 31.6%). Satellites features 1 and 2 also diminished substantially, with decreases of 13.9% to 7.0% and 5.4% to 4.0%, respectively. This compositional shift suggests partial surface oxidation of Co^2+^ to Co^3+^ upon aging, but it could also be due to Co^2+^ dissolution along the aging process.^71^ Nevertheless, both scenarios are consistent with a loss of satellite intensity, which was linked previously to the presence of Co^2+^ sites at the catalyst surface. This behavior is also corroborated by the deconvoluted XANES components by PCA (Figure S9), particularly Component 2 that showed a clear shift to lower oxidation states. As the latter ions are primarily responsible for final state transitions due to their unpaired d electrons, their decrease leads to a concomitant reduction in satellite intensity, indicating suppressed inelastic scattering and shakeup phenomena. Additionally, the cobalt components showed small shifts toward lower binding energies (∼0.1–0.7 eV). Regarding the O 1s window, all bands showed negligible binding energy shifts (∼0.4 eV) and a considerable change in relative area for O_lat_ (from 24.1% to 40.5%). The significant increase in the O_lat_ component, typically assigned to lattice oxygen, alongside a relative decrease in surface O adsorbed species (O_ads_), can be assigned to the higher Co^3+^ content, as these species are coordinated to more oxygen atoms than Co^2+^, consistent with surface restructuring. Fitting results of O 1s show that the O_ads_/O_lat_ ratio decreases 46.8% after cycling, which means a lower ratio of adsorbed oxygen species on aged Co_3_O_4_-np. A similar analysis was conducted for Co_3_O_4_ nanoplates, in which Lu et al. evaluated the activity of the facets (111) and (112) toward NO_3_RR.^98^ They reported an increase in Co^3+^ exposure and adsorbed oxygen on the former plane, assigning it as a possible reason for its superior selectivity for NH_3_ than the latter plane.

The XRD-refined lattice parameters of the Co_3_O_4_-np spinel phase (*a* = *b* = *c* = 9.96 Å, α = β = γ = 90°, volume = 986.95 Å^3^, density = 4.63 g cm^–3^) reveal a slight expansion, suggesting lattice swelling (Figure 4E). The refinement achieved agreement indexes of *R*_W_ = 9.71% and GOF = 1.95 using the same Co_3_O_4_ JCPDS card as the pristine catalyst. The low angle region also reveals a decay in intensity associated with the sample amorphism. The same does not occur on pristine Co_3_O_4_-np/Au diffractogram (Figure 1F). Figure 4F displays in situ XANES spectra of aged Co_3_O_4_-np/GC collected under various applied potentials. As observed in the pristine sample (Figure 1G), no discernible changes in crystal phase or cobalt oxidation states are detected across the Faradaic potential range, indicating the catalyst’s bulk structure remains intact. Additionally, Figure 4G,H shows the same spectral components as pristine Co_3_O_4_/GC, Figure 1H,I. Their distribution after the aging protocol became more homogeneous and evenly dispersed, reflecting an increased contribution from the more oxidized component, Component 2. This evolution could be attributed to partial surface oxidation of Co^2+^ to Co^3+^ and/or Co^2+^ dissolution along the aging process, in agreement with the XPS results. These effects are attributed to defect formation, as evidenced by high-resolution electron micrographs, which also correlate with an increase in O_ads_ species and a higher Co^3+^ surface population. The surface changes are likely responsible for the observed enhancement in NO_3_RR activity and the simultaneous loss of NH_3_ selectivity (Figure 3), however, it is still necessary to unravel how the aging protocol affects the NO_3_RR mechanism. EC-MS data did not identify changes in the mechanism pathways, only in the relative distribution of its products (Figure S13). The question now becomes why do these surface reconstructions induce different amounts of products in the first place?

### 3.5 Theoretical Modeling and Molecular Characterization

Our experimental analysis of the Co_3_O_4_-np catalyst in both pristine and aged states reveals pronounced morphological and surface reconstruction upon aging, most notably manifested as a decrease in the Co^2+^/Co^3+^ ratio. EIS shows a systematic enhancement of the CT process with catalyst aging, indicative of accelerated interfacial electron-transfer kinetics involving adsorbed species. Product analysis further demonstrates that this improved charge-transfer capability is primarily manifested as enhanced HER activity and, to a lesser extent, as an increased rate of ammonia production from NO_3_RR. Nevertheless, this shift in reaction kinetics occurs concomitantly with an overall decrease in the average FE toward NH_3_, reflecting the preferential acceleration of the competing HER pathway. To elucidate the molecular-level origin of the observed structure–activity relationships and to assess how variations in the Co^2+^/Co^3+^ ratio modulate catalytic performance and reaction pathways, DFT calculations were performed using the Vienna Ab initio Simulation Package (VASP). The electron–ion interactions were described by the projector augmented-wave method, and the exchange–correlation energy was treated with the generalized gradient approximation parametrized by Perdew, Burke, and Ernzerhof (PBE). To accurately describe the strongly localized d-electron states of Co, the rotationally invariant PBE + U scheme was applied with an effective Hubbard parameter of *U*_eff_ = 3.50 eV. Long-range van der Waals interactions were included using the empirical Grimme D3 dispersion correction. For the pristine slabs and adsorbate–slab systems, the plane-wave kinetic energy cutoff was set to 488.734 eV. A vacuum spacing of 15 Å was employed to suppress spurious interactions between periodic images. Geometry optimizations were initially conducted at the Γ-point, followed by single-point energy evaluations on a (3 × 3 × 1) *k*-point mesh. The convergence criteria were set to 10^–6^ eV for the electronic loop and 0.01 eV/Å for the maximum residual force on each atom. A full description of the structural relaxation, magnetic configuration, and gas-phase reference calculations is provided in the Supporting Information. Three Co_3_O_4_(111) surface terminations with distinct oxidation states were constructed: Co_3_O_4_ (111)-O (oxidized), Co_3_O_4_(111)-S (stoichiometric), and Co_3_O_4_(111)-R (reduced), with the oxidized and reduced models representing the aged and pristine catalysts, respectively. Key reaction intermediates for nitrate reduction (*NO_2_ and *ONOH), along with the sole HER intermediate (*H), were systematically evaluated to identify their most thermodynamically stable adsorption configurations (Figure 5A). For hydrogen adsorption, lattice oxygen atoms act as the preferred binding sites on both the oxidized and stoichiometric Co_3_O_4_(111) surfaces. In contrast, on the reduced Co_3_O_4_(111)-R termination, the octahedral Co^3+^ center emerges as the most favorable adsorption site. This site’s preference arises from the lower coordination environment and increased electronic flexibility of metal cations on the reduced surface, structural features absent on the oxidized facet. Furthermore, electronic structure analysis reveals a Bader charge shift, (Δ*Q* ^*i*^_eff_), of magnitude 0.23e between analogous atoms on these reduced and stoichiometric surfaces, suggesting that local charge redistribution plays a pivotal role in modulating the adsorption tendency (Figure 5C).

**Figure 5:**
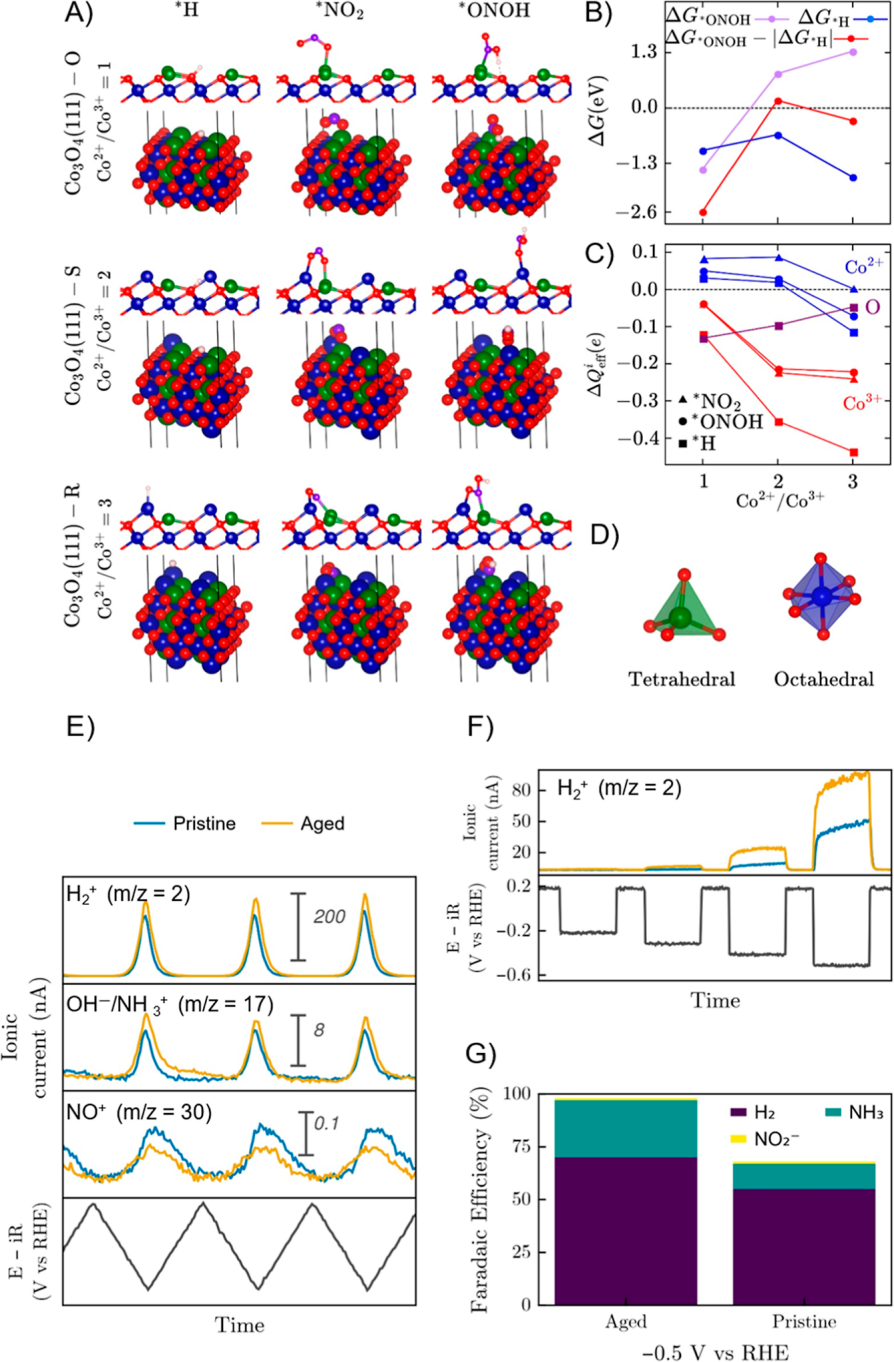
Investigation of NO_3_RR mechanism. (A) Side and perspective views, rendered in ball-and-stick and space-filling models respectively, illustrating the most energetically favorable reaction intermediate configurations (*H) identified via computational screening on Co_3_O_4_(111) surfaces exhibiting distinct oxidation states. Green and blue spheres distinguish the tetrahedral and octahedral cobalt sites, respectively. Light pink and red spheres denote hydrogen and oxygen atoms, respectively. These systems are categorized by rows and annotated with their corresponding Co^2+^/Co^3+^ ratios. (B) Comparative energetic analysis of the HER pathway, elucidating the thermodynamic competition and the resultant thermodynamic selectivity. (C) Differential Bader charge analysis quantifying the net electronic charge-transfer for surface atoms *i* proximal to the adsorbate, calculated as the difference between the pristine and adsorbed counterparts. (D) Tetrahedral and octahedral Co coordinations. (E) H_2_^+^, OH^–^/NH_3_^+^, and NO^+^ ionic currents registered on a CV protocol at a scan rate of 10 mV s^–1^ for both pristine and aged Co_3_O_4_-np samples. (F) H_2_^+^ ionic current recorded on CA protocol from −0.2 to −0.5 V vs RHE for both pristine and aged catalysts. (G) Faradaic efficiencies for both catalysts at −0.5 V vs RHE.

For nitrogen-containing intermediates, adsorption on the Co_3_O_4_(111)-O surface preferentially occurs at coordinatively unsaturated cobalt sites rather than lattice oxygen atoms. In particular, the *trans*-HONO (*ONOH) intermediate binds through a Co–N interaction, which is thermodynamically stabilized by a molecular reorientation that enables hydrogen bonding between the adsorbate and a surface oxygen anion. Conversely, adsorbed *NO_2_ preferentially adopts an O-bound coordination mode. This behavior is consistent with Frontier molecular orbital considerations, whereby an N-bound configuration of *ONOH maximizes orbital hybridization between nitrogen p-orbitals and cobalt 3d-states, while O link NO_2_ adsorption is dominated by electrostatic interactions, in agreement with binding mechanisms reported for related spinel oxide catalysts.^99^ These adsorption trends are largely preserved across surface terminations, with a notable exception observed for the stoichiometric Co_3_O_4_(111)-S surface. In this case, *trans*-HONO preferentially coordinates through an oxygen atom to an octahedral Co^3+^ site, representing the sole configuration in which this cation geometry constitutes the thermodynamically preferred adsorption center. In contrast, for both oxidized and reduced terminations, Co^2+^ species consistently act as the dominant adsorption sites. For the stoichiometric and reduced surfaces, the geometric protrusion of cobalt atoms into the vacuum region, combined with pronounced local charge heterogeneity, promotes multidentate binding motifs and facilitates the formation of bridging adsorption configurations for polyatomic species, a phenomenon that is particularly pronounced on the reduced surface.

The thermodynamic landscape governing NO_3_RR was further analyzed by focusing on the reaction bottleneck, employing the Gibbs free energy of *ONOH adsorption (ΔG_*ONOH_) as a descriptor for ammonia selectivity, while ΔG_*H_ serves as the primary descriptor for HER within the computational hydrogen electrode formalism. As shown by the blue trace in Figure 5B, the oxidized and stoichiometric surfaces, representative of the experimentally aged catalyst and characterized by low Co^2+^/Co^3+^ ratios, exhibit ΔG_*H_ values approaching thermoneutrality. According to the Sabatier principle,^100^ this near-zero free energy reflects an optimal balance between proton adsorption and desorption, thereby favoring efficient hydrogen evolution. This theoretical prediction is fully consistent with experimental EC-MS and GC measurements, which reveal enhanced HER activity for the aged catalyst (Figure 5E–G). In contrast to the H_2_^+^ and NH_3_^+^/OH^–^ fragments, aged Co_3_O_4_-np exhibit a lower ionic current intensity for the NO^+^ fragment. This behavior can be attributed to the higher catalytic activity toward NH_3_ formation, reflected by the increased NH_3_ YR values. In electrochemical experiments coupled with mass spectrometry, the magnitude of the recorded ion current is directly proportional to the incoming flux of the corresponding species (d*n*/d*t*; mol s^–1^).^101^ Therefore, the higher NH_3_ YR values observed for aged Co_3_O_4_-np are likely a consequence of the rapid conversion of *NO_2_ to *NO, followed by fast consumption, as *NO is more rapidly hydrogenated to NH_3_. As a result, the residence time of NO-containing intermediates is reduced, leading to a lower probability of NO^+^ species reaching the detector. The opposite behavior is therefore expected for pristine, which is indeed observed and agrees with theoretical predictions for the reduced Co_3_O_4_(111)-R surface, which is representative of pristine.

The reduced Co_3_O_4_(111)-R surface exhibits a pronounced deviation of ΔG_*H_ from thermoneutrality, indicating a nonoptimal hydrogen binding strength and rendering HER thermodynamically less favorable. Analysis of the lilac trace, which captures the thermodynamic propensity for *NO_2_ trapping, reveals a clear structure–property relationship: increasingly negative free energies correspond to enhanced stabilization and deep trapping of *NO_2_. In contrast, the analysis delineates a fundamental structure–property relationship in which the tendency to retention of *NO_2_ scales directly with the surface cation stoichiometry; specifically, an increase in the Co^2+^/Co^3+^ ratio stabilizes the adsorbed species *NO_2_, thus shifting the thermodynamic landscape to favor its capture. Such a theoretical prediction appears to support the more pronounced ionic current for the NO^+^ fragment observed for the pristine catalyst (Figures 5E, S13 and S14), which may indicate a lower consumption rate of *NO toward NH_3_ formation, together with enhanced thermodynamic stabilization of *NO_2_ on this surface. In combination with the predicted thermodynamically less favorable HER, the pristine catalyst is therefore expected to be relatively disfavored for the NO_3_RR. This interpretation is consistent with the calculated NH_3_ YR, despite the comparatively higher Faradaic efficiency (49.4% ± 0.5%) observed at –0.3 V vs RHE. Experimentally, this behavior is further suggested by the incomplete screening of the reaction products at −0.5 V vs RHE, where the total detected products account for less than 70% of the charged passed, Figure 5G. A minor contribution from uncounted byproducts, such as NH_2_OH and N_2_, cannot be excluded. However, no evidence for their formation at appreciable concentrations was found, using complementary techniques, including ^1^H NMR, GC, and EC-MS. An additional explanation for this discrepancy may lie in the dynamic structural and electronic evolution of the pristine Co_3_O_4_-np during electrolysis. In this context, the delayed establishment of a steady state could be associated with surface reconstruction processes, which may be accompanied by partial dissolution and redeposition phenomena.^75^

The thermodynamic viability of the competing reaction channels is systematically assessed using two primary descriptors. First, the absolute value of the Gibbs free energy of hydrogen adsorption, |ΔG_*H_|, is used as a key metric that defines the potential-determining step for HER, inherently incorporating both the energetics of proton adsorption and subsequent H_2_ desorption. Second, the differential descriptor ΔG_*ONOH_ – |ΔG_*H_| provides a quantitative measure of the selectivity balance between ammonia formation and the parasitic HER. More negative values of these descriptors identify surfaces with pronounced bifunctional character, exhibiting enhanced activity toward both hydrogen evolution and nitrogen-containing intermediates. This optimal catalytic regime is exemplified by the surface model that best represents the experimentally aged catalyst. Experimentally, this manifests as sustained H_2_ evolution over a broad potential window (Figure 5E) and, at sufficiently negative potentials (−0.5 V vs RHE), a higher FE toward NH_3_ compared to the pristine catalyst, Figure 5G. Conversely, further deviation of the surface cation ratio from unity, as in the pristine catalyst representative, shifts the free-energy profile away from the optimal binding-energy window prescribed by the Sabatier principle, and classifies these surfaces as intrinsically poor catalysts for both reaction networks. Experimentally, this behavior was not observed for the pristine catalyst at −0.3 V vs RHE. However, at sufficiently negative potentials (−0.5 V vs RHE), the pristine catalyst exhibited lower Faradaic efficiencies for both H_2_ and NH_3_ compared with the aged catalyst.

To develop a fundamental electronic rationale for the observed reactivity trends, we evaluated the effective Bader charge differential, which quantifies the net change in the partial charge of a given surface atom *i* upon adsorption, relative to its charge in the pristine, adsorbate-free surface. For the nitrogen-containing intermediates *NO_2_ and *ONOH, the lattice oxygen anions do not act as primary adsorption centers; accordingly, their charge perturbation, *Q* ^O^_eff_, remains consistently negligible and is therefore omitted from the analysis to focus on the electronically active metal sites. In contrast, hydrogen adsorption exhibits a distinctly different electronic signature, as illustrated by the purple trace in Figure 5C. The data indicate that the most strongly bound configurations correspond to geometries in which the hydrogen adatom is spatially decoupled from the lattice oxygen, yielding *Q* ^O^_eff_ values that approach zero. This behavior suggests that Co^3+^ sites on the electronically balanced surface display a high degree of electronic rigidity, undergoing minimal charge modulation irrespective of the adsorbate. Consequently, the thermodynamics of HER are governed predominantly by the capacity of the oxygen sublattice to support moderate charge-transfer. In contrast, abrupt and large-amplitude charge depletion at Co^3+^ centers correspond to excessively strong thermodynamic stabilization of adsorbates, which is detrimental to catalytic turnover. Moreover, on surfaces characterized by elevated Co^2+^/Co^3+^ ratios, the short interatomic separations and low coordination numbers of the cobalt sites render them highly effective electron acceptors, as reflected in the pronounced decrease of the metal charge upon adsorption. This enhanced electron-accepting character promotes strong binding. This contrast provides a mechanistic explanation for why the aged surface more effectively suppresses *NO_2_ poisoning: its restricted charge-transfer capacity disfavors the formation of the overly stable adsorption complexes that dominate on the more strongly reduced substrate.

## 4 Conclusions

This study evaluates the performance of Co_3_O_4_-np/GC for NO_3_RR. Through both in situ and ex situ characterizations, we demonstrate that the spinel phase remains stable throughout chronoamperometric measurements. The material’s activity was assessed for both NO_3_RR and NO_2_RR, with the best results observed for NO_2_^–^, achieving FE of 92.5 ± 7.5% and YR of 45.6 ± 4.1 μmol h^–1^ cm^–2^. To investigate long-term stability, we subjected the catalyst to 1000 cycles of CV, revealing that aging leads to loss of crystallinity at the nanoplates edges, as shown by HRTEM micrographs, also revealing aggregation among the hexagon nanoplates. SEM micrographics reveal a slight expansion in the hexagon’s length, but no real alteration on their width. XPS reveals an increase in the Co^2+^/Co^3+^ ratio followed by a reduction in O adsorbed species. These changes correlate to a simultaneous decrease in the NH_3_ selectivity and a significant increase in its production rates. Furthermore, DRT profiles of the sample before and after aging reveal improvement in the CT kinetics and lowering of the energy barrier of MT polarization, which explains the increase in YR after cycling. The combination of theoretical and experimental data provides a well-structured framework for describing the evolution of Co_3_O_4_-np and the corresponding variations in the reaction pathways during NO_3_RR and NO_2_RR. Our results indicate that pristine catalysts exhibit a Co^2+^/Co^3+^ ratio and surface organization that presumably promote stronger interaction with *NO_2_, together with a relatively disfavored *H stabilization. This surface chemistry is primarily reflected in a lower NH_3_ YR. Aging is an effective strategy to enhance the surface activity of the pristine catalyst by tuning the Co^2+^/Co^3+^ ratio, resulting in more balanced *H and *NO_2_ adsorption–desorption dynamics. This modification promotes HER to some extent but also substantially enhances the NH_3_ YR, indicating improved overall NO_3_RR kinetics. These observations offer promising opportunities to further optimize NO_3_RR activity on an aged-like Co_3_O_4_-np catalyst by systematically tuning pH, electrolyte composition, and NO_3_^–^ concentration.

## Supplementary Material



## Notes

Standard addition NH_3_ quantification curve postelectrolysis, Co­(OH)_2_-np micrographs, length and width histograms of pristine Co_3_O_4_-np, complete Co window XPS spectrum of Co_3_O_4_-np/Au, PCA analysis/correlation circle of different Co_3_O_4_-np/Au XPS spectra postelectrolysis and CV, XANES pre-edge features and first derivatives of both pristine and aged Co_3_O_4_-np/GC, deconvoluted DRT spectrum and EIS tables of circuit fitting and DRT maps, and length and width histograms of the postaged sample (PDF)
