# Supplementary material for: Electrochemical Nitrate and Nitrite Reduction Reaction to Ammonia: Catalytic Aging and Stability of Co3O4 Hexagonal Nanoplates
Source: ACS Appl Mater Interfaces. 2026 May 20;18(21):29982–97. doi: 10.1021/acsami.6c03616 (PMC13249119; doi:10.1021/acsami.6c03616)
Supplement: Supplementary file 1 [file am6c03616_si_001.pdf]

Supporting Information

Electrochemical Nitrate and Nitrite Reduction Reaction to Ammonia: Catalytic Aging and Stability of Co_3O_4 Hexagonal Nanoplates

Matheus P. Sales[a], Maykon L. Souza[a,b,c], Marionir C. Branco[d], Samuel C. Silva[a], João P. B. Da Silva[a], Rafael G. Yoshimura[a,e], Rafael L. Romano[d], João J. Mauricio[a] , Manuel E. G. Winkler[a,b], Kauan L. Gomes[a], João B. Souza Jr.[a,f], Santiago J. A. Figueroa[a,e], Fabio H. B. Lima[d], Cláudio F. Tormena[a], Juarez L. F. Da Silva[d], Italo O. Mazali[a], Raphael Nagao[a,b,]*

[a]*Institute of Chemistry, University of Campinas, Campinas, SP 13083-862, Brazil;*

[b]*Center for Innovation on New Energies, University of Campinas, Campinas, SP 13083-084, Brazil;*

[c]*Department of Chemistry, Federal University of São Carlos, São Carlos, SP 13565-905, Brazil;*

[d]*University of São Paulo, São Carlos Institute of Chemistry, São Carlos, SP 13566-590, Brazil;*

[e]*Brazilian Synchrotron Light Laboratory, Brazilian Center for Research in Energy and Materials, Campinas, SP 13083-100, Brazil;*

[f]*Brazilian Nanotechnology National Laboratory, Brazilian Center for Research in Energy and Materials, Campinas, SP 13083-100, Brazil;*

*Corresponding author: nagao@unicamp.br

List of Figures

Figure S1. UV-vis spectra and standard addition curve for NH_3 after NO_3RR at –0.3 V_{RHE} in NaOH 1 mol L^{-1} and $NaNO_3$ 20 mmol L^{-1}. 6

Figure S2. ^{1}H NMR spectrum of $^{15}NH_4^+$ (in blue) and $^{14}NH_4^+$ (in blue), in the left side, for the electrolysis of nitrate. The signal on right corresponds to the DMSO-d6, used to lock the sample in NMR and as to reference. A) qualitative H-NMR of NH_3 sample produced after 1-h chronoamperometry at –0.5 V_{RHE} in 1 mol L^{-1} NaOH and 0.1 mol L^{-1} $NaNO_3$. B) comparison between qNMR and UV-Vis quantification methods after 1-h chronoamperometry in 1 mol L^{-1} NaOH and 0.5 mol L^{-1} $NaNO_3$..8

Figure S3. UV vis spectra and NO_2^- external calibration curve, obtained from Griess method. 9

Figure S4. Length (189 ± 38 nm) and width (37 ± 12 nm) histograms of pristine Co_3O_4–np/Au measured from SEM micrographs. 11

Figure S5. $Co(OH)_2$-np top-view HRTEM image. 12

Figure S6. Deconvoluted pristine Co_3O_4–np/Au Co 2p XPS spectrum displaying both $2p_{3/2}$ and $2p_{1/2}$ bands. 13

Figure S7. O 1s XPS spectra of Co_3O_4–np/Au samples after different electrochemical treatments (pristine, –0.2 V_{RHE} chrono in $NaNO_2$ 20 mmol L^{-1} + NaOH 1 mol L^{-1}, 10 cycles of CV from +0.1 to –0.6 V_{RHE} at 20 mVs^{-1} in the same electrolyte and 10 cycles of CV in the same previous conditions, but with $NaNO_3$ 20 mmol L^{-1} instead). 15

Figure S8. PCA of XPS spectra from Co_3O_4–np/Au samples. Both loading plots of O 1s and Co 2p are displayed along the corresponding correlation circle. 16

Figure S9. XANES spectra around Co_3O_4 K-edge of Co, CoO, Co_3O_4 and CoOOH samples, as well as Components 1 and 2 obtained from PCA analysis by ITFA algorithm. This procedure allows to align theoretical components obtained from PCA to physical meaningfully spectra.[11,12] The insert was taken in between the K-edge and pre-edge excitations. As can be seen Component 1 are close to Co_3O_4 and Component 2 can be identified with CoOOH, it can be established that all the peaks and valleys of both spectra in the deconvoluted ITFA Slight differences in oxidation state are visible in Component 2 related to CoOOH, probably due to a remanent Co^{2+} in the surfaces, as can be shown in the XPS analysis. 17

Figure S10. Circuit fit results of EIS data taken across Faradaic potentials for both pristine and aged Co_3O_4–np/GC...23

Figure S11. Deconvoluted DRT spectrum of aged Co_3O_4–np/GC at 0 V vs RHE. Deconvolution performed with the Julia package Spectra.[17] 24

Figure S12. Length (199 ± 43 nm) and width (35 ± 6 nm) histograms of aged Co_3O_4–np/Au measured from SEM micrographs. 25

Figure S13. Pristine and aged Co_3O_4-np ionic fragments (H_2^+, NH_2OH^+, NO^+, N_2^+, NH_3^+) measured by EC-MS following CV protocol. 26

Figure S14. Pristine and aged Co_3O_4-np ionic fragments (H_2^+, NH_2OH, NO, N_2, NH_3, H_2) measured by EC-MS following CA protocol. 27

Figure S15. H_2, N_2 and O_2 chromatogram signals of Co_3O_4-np post-electrolysis headspace, before and after aging protocol, registered by a TCD detector. The N_2 and O_2 signals observed in the TCD are attributed to trace amounts of ambient air introduced into the needle dead volume during gas sampling and injection. The syringe's full gas-tightness is preserved. 28

Figure S16. Geometric parameters of the molecular species involved in the NO_3RR process. Light pink, purple, and red spheres denote hydrogen, nitrogen, and oxygen atoms, respectively, thereby highlighting the atomic composition and bonding environment...30

Figure S17. Geometric parameters of molecules adsorbed on surfaces, illustrated by: a) interatomic distances between molecular atoms and surface sites, and b) molecular orientation angles relative to the

plane parallel to the z-axis of the supercell (highlighted by the horizontal black line). Green, blue, red, and light-pink spheres represent tetrahedral Co, octahedral Co, oxygen, and hydrogen atoms, respectively. ..31

Figure S18. a) Simplified mechanism illustrating the pathway from the nitrate to the possible products, with red arrows indicating the specific routes considered in the simulation of nitrite formation. b) Snapshots of the theoretical components in a closed system, used for the DFT calculations within the CHE framework. c) The feasible substitution of components that are actually included in the computational evaluation. Dark grey spheres represent surface atoms...37

Figure S19. a) Simplified mechanism illustrating the HER pathway. b) Representative snapshots of the theoretical components in a closed system, employed in the DFT calculations within the CHE framework. c) Feasible substitutions of the components actually incorporated into the computational evaluation. Dark grey spheres represent surface atoms, and light pink spheres correspond to hydrogen. d) Generic process used to exemplify the Nernstian correction applied to the HER process. 40

Figure S20. Crystal structure of the Co_3O_4 spinel: a) conventional unit cell, b) primitive cell, and c) distinct cobalt coordination environments. Cobalt atoms in green occupy tetrahedral sites, whereas cobalt atoms in blue occupy octahedral sites. Oxygen atoms are shown in red, and dashed lines indicate the unit cell boundaries...43

Figure S21. Total/partial Density of State (Total/P-DOS) for spinel Co_3O_4. Black dashed vertical lines represent the energy reference, which is the valence band maximum (VBM) for semiconductors. 44

Figure S22. Top and side views of the $Co_3O_4(111)$ (1×1) surface, emphasizing the exposed terminations that determine the relative proportions of cobalt species. Green, blue, and red spheres represent Co^T, Co^O, and O atoms, respectively. Dashed lines indicate the boundaries of the supercell....................................45

Figure S23. (a) Adsorption sites on the three $Co_3O_4(111)$ surfaces S, R and O, considered for adsorption, together with the corresponding initial configurations of (b) NO2 and (c) trans-HONO on the surface. Green, blue, red, purple and light pink spheres represent Co^T, Co^O, O, N and H atoms, respectively. 47

Figure S24. Pourbaix diagram determined by the theoretical potential required for the formation of the *ONOH intermediate. Contrasting the $Co_3O_4(111)$-S, $Co_3O_4(111)$-R, and $Co_3O_4(111)$-O surfaces, delineating the thermodynamic stability regions associated with the potential-determining steps. 55

List of Tables

Table S1. Comparison metrics between different catalysts for NO_3RR and NO_2RR. 10

Table S2. Pristine Co_3O_4-np/GC EIS circuit fitting parameters across Faradaic potentials. 23

Table S3. Aged Co_3O_4-np/GC EIS circuit fitting parameters across Faradaic potentials. 23

Table S4. Specific technical parameters of the PAW-PBE projectors employed in this work, including the recommended plane-wave cutoff energy, ENMAX (eV), the number of valence electrons ($Zval$), and the corresponding valence electronic configuration. 29

Table S5.. Bond lengths $dH-H$, $dO-H$ and $dO-O$, Molecule angle αmol, binding energy Eb of the Molecule in the gas phase, and its percentage deviation ΔEb from the reference values $Ebref$ expressed in parenthesis. 41

Table S6. Calculation results of vibrational frequencies, showing energy values for ZPE, $CpdT$, $-TS$, and Gibbs energy G. All thermodynamic conditions are considered under standard settings of T =298.15 K and P =1 bar...42

Table S7. Convergence of the lattice parameter ($a0$) and relative energy ($\Delta Etot$) for the primitive bulk spinel Co_3O_4 unit cell, obtained from stress tensor relaxation calculations as a function of the k-point mesh ($n \times n \times n$) and the total number of k-points in the Brillouin zone (Nk). 43

Table S8. Surface properties, including the surface energy (σs), and magnetic moment expressed in units of the Bohr magneton ($\mu/\mu\beta$), were evaluated for the atoms located at the outermost layer, i.e., those directly exposed to vacuum. The calculated values for these surface atoms are compared with reference data available in the literature.[40] 46

Table S9. Geometric characteristics and adsorption energy, ΔEad, for H on Co_3O_4(111)-S, considering the initial adsorption sites (site i) and their corresponding final positions (site f) after full geometric optimization. 47

Table S10. Geometric parameters and adsorption energies, ΔEad, for $*NO_2$ on the Co_3O_4(111)-S surface are presented, taking into account the initial adsorption sites and their corresponding optimized configurations (site i-f). The structural arrangements after relaxation are indicated by labels 1–5 in parentheses, in accordance with Figure S23-b, following full geometrical optimization. 48

Table S11. Geometric parameters and adsorption energies, ΔEad, for trans-HONO on the Co_3O_4(111)-S surface are presented, taking into account the initial adsorption sites and their corresponding optimized configurations (site i-f). The structural arrangements after relaxation are indicated by labels 1–6 in parentheses, in accordance with Figure S23-c, following full geometrical optimization....................48

Table S12. The energetic contributions ZPE, $\int Cp\ dT$, and $-TS$ within the scope of the CHE model for the determination of G in systems comprising adsorbed intermediates in their most thermodynamically stable configurations, taking Co_3O_4(111)-S as the reference surface. 49

Table S13. Geometric characteristics and adsorption energy, ΔEad, for *H on Co_3O_4(111)-R, considering the initial adsorption sites (site i) and their corresponding final positions (site f) after full geometric optimization. 49

Table S14. Geometric parameters and adsorption energies, ΔEad, for $*NO_2$ on the Co_3O_4(111)-R surface are presented, taking into account the initial adsorption sites and their corresponding optimized configurations (site i-f). The structural arrangements after relaxation are indicated by labels 1–5 in parentheses, in accordance with Figure S23-b, following full geometrical optimization. 49

Table S15. Geometric parameters and adsorption energies, ΔEad, for trans-HONO on the Co_3O_4(111)-R surface are presented, taking into account the initial adsorption sites and their corresponding optimized configurations (site i-f). The structural arrangements after relaxation are indicated by labels 1–6 in parentheses, in accordance with Figure S23-c, following full geometrical optimization....................51

Table S16. The energetic contributions ZPE, $\int Cp\ dT$, and $-TS$ within the scope of the CHE model for the determination of G in systems comprising adsorbed intermediates in their most thermodynamically stable configurations, taking Co_3O_4(111)-R as the reference surface....................51

Table S17. Geometric characteristics and adsorption energy, ΔEad, for H on Co_3O_4(111)-O, considering the initial adsorption sites (site i) and their corresponding final positions (site f) after full geometric optimization. 51

Table S18. Geometric parameters and adsorption energies, ΔEad, for $*NO_2$ on the Co_3O_4(111)-O surface are presented, taking into account the initial adsorption sites and their corresponding optimized configurations (site i-f). The structural arrangements after relaxation are indicated by labels 1–5 in parentheses, in accordance with Figure S23-b, following full geometrical optimization. 52

Table S19. Geometric parameters and adsorption energies, ΔEad, for trans-HONO on the Co_3O_4(111)-O surface are presented, taking into account the initial adsorption sites and their corresponding optimized configurations (site i-f). The structural arrangements after relaxation are indicated by labels 1–6 in parentheses, in accordance with Figure S23-c, following full geometrical optimization....................52

Table S20. The energetic contributions ZPE, $\int Cp\ dT$, and $-TS$ within the scope of the CHE model for the determination of G in systems comprising adsorbed intermediates in their most thermodynamically stable configurations, taking Co_3O_4(111)-O as the reference surface....................53

Table S21. Performance of HER, and NO_3RR on the slabs used in the description, expressed by ΔG (with an applied potential of U = 0.0 V and pH=0), relative to the initial reaction state preceding the PCET step in both reactions...53

Supporting Figures

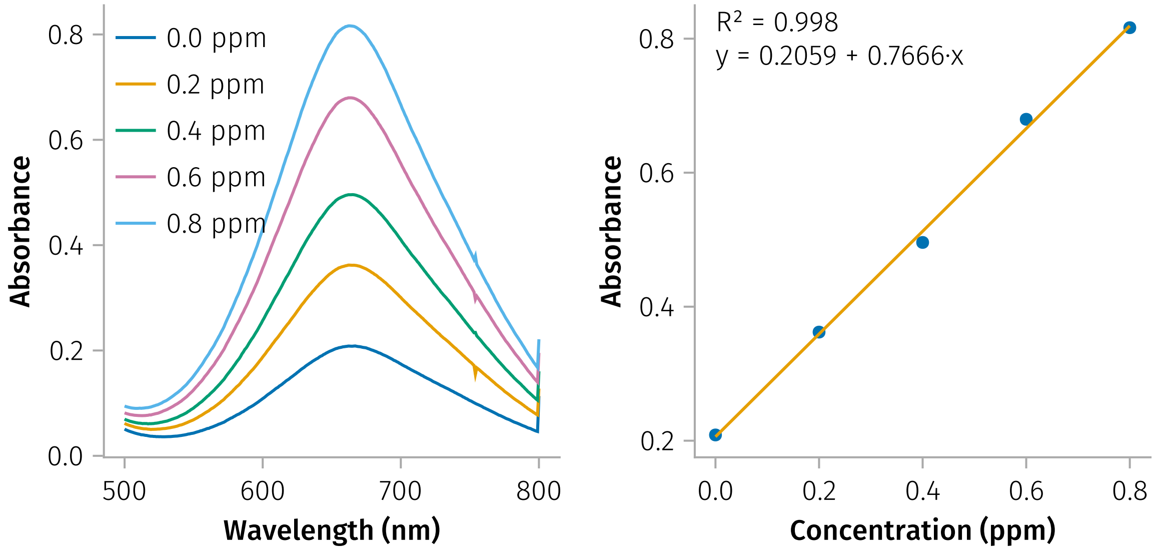

Figure S1. UV-vis spectra and standard addition curve for NH_3 after NO_3RR at –0.3 V_{RHE} in NaOH 1 mol L^{-1} and $NaNO_3$ 20 mmol L^{-1}.

Supplementary note 1. Hydrogen nuclear magnetic resonance (^{1}H-NMR).

Nuclear Magnetic Resonance was used to confirm the ammonium conversion and as a comparative quantification method. The samples are prepared with 500 µL of the solution from the electrochemical cell after the acidification for pH=3, with the addition of $DMSO_2$ as internal standard. 50 µL DMSO-d6 are also add to the sample to ensure the lock in equipment. The NMR experiments were conducted in Bruker Advance Neo spectrometer, operating at 16.1 T, equipped with Prodigy probe. Quantitative experiments are performed using a 1D pulse sequence with an excitation sculpting block for water suppression. The relaxation delay used was 10 times the higher T1 value, obtained in an inversion recovery experiment, also with an excitation sculpting suppression. To achieve an ideal signal-to-noise ratio, 128 scans are collected. The absolute method for quantification was used to calculate the $^{14}NH_4^+$ or $^{15}NH_4^+$ mass in the sample, assuming 100% of purity for the analyte.

$$m_x = \frac{I_x}{I_{cal}} \frac{N_{cal}}{N_x} \frac{M.M_x}{M.M_{cal}} \frac{P_{cal}}{P_x} m_{cal} \quad (1)$$

where the m is the mass, I is the Integral, N is the number of nuclei for the signal, $M.M$ is the molar mass and P is the purity for the analyte (x) and internal standard (cal).

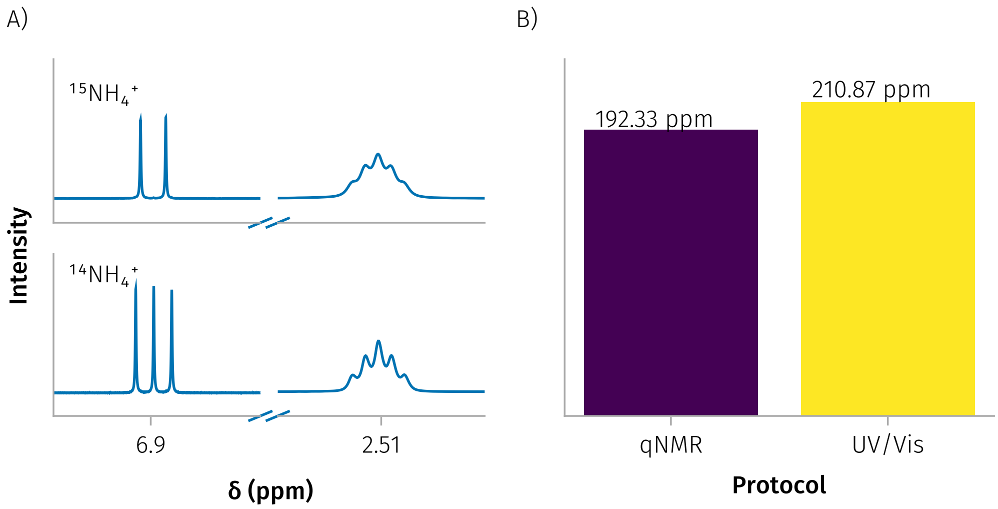

Figure S2. ^1H NMR spectrum of $^{15}NH_4^+$ (in blue) and $^{14}NH_4^+$ (in blue), in the left side, for the electrolysis of nitrate. The signal on right corresponds to the DMSO-d6, used to lock the sample in NMR and as to reference. A) qualitative H-NMR of NH_3 sample produced after 1-h chronoamperometry at –0.5 V_{RHE} in 1 mol L^{-1} NaOH and 0.1 mol L^{-1} $NaNO_3$. B) comparison between qNMR and UV-Vis quantification methods after 1-h chronoamperometry in 1 mol L^{-1} NaOH and 0.5 mol L^{-1} $NaNO_3$.

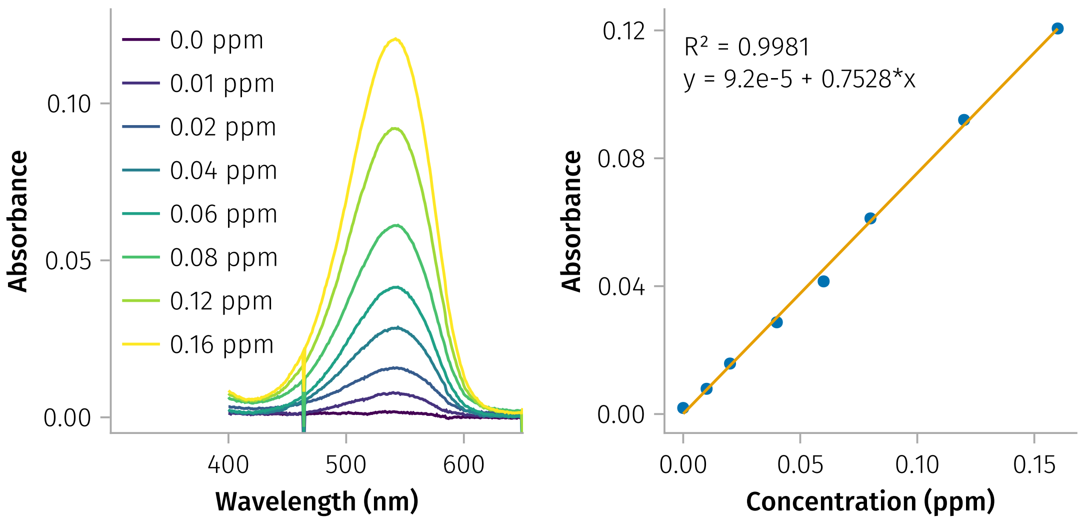

Figure S3. UV vis spectra and NO_2^- external calibration curve, obtained from Griess method.

Table S1. Comparison metrics between different catalysts for NO_3RR and NO_2RR.

Catalyst	Electrolyte	Performance	E_{RHE} (V)	Ref
Co_3O_4-np	1 mol L^{-1} NaOH + 20 mmol L^{-1} $NaNO_3$	FE = 49.4 % YR = 8.5 µmol h^{-1} cm^{-2}	-0.3	This work
MnPc/RGO	0.1 mol L^{-1} K_2SO_4 + 0.2 mol L^1 KNO_3	FE = 98.3 % YR = 20316 µg h^{-1} mg_{cat}^{-1}	-1.5	[1]
3D nanorod array of $Fe(TCNQ)_2$	0.1 mol L^{-1} Na_2SO_4 + 0.2 mol L^{-1} $NaNO_3$	FE = 85.2% YR = 11351.6 µg h^{-1} mg_{cat}^{-1}	-1.1	[2]
MnPc	0.2 mol L^{-1} Na_2SO_4 + 0.1 mol L^{-1} $NaNO_2$	FE = 92.9% YR = 16603.4 µg h^{-1} mg_{cat}^{-1}	-0.9	[3]
FePc nanotube	0.1 mol L^{-1} K_2SO_4 + 0.5 mol L^{-1} KNO_3	FE = 100% YR = 35067.09 µg h^{-1} mg_{cat}^{-1}	-1.5	[4]
**β**-CuPc	0.1 mol L^{-1} Na_2SO_4 + 0.25 mol L^{-1} $NaNO_3$	FE = 96% YR = 703 µg h^{-1} mg_{cat}^{-1}	-1.1	[5]
Co/Cu mixed oxide-derived	1 mol L^{-1} NaOH + 20 mmol L^{-1} $NaNO_3$	FE = 94.4% YR = 44 µmol h^{-1} cm^{-2}	-0.2	[6]
$Co_3O_4/TiO_2/Ti$	1 mol L^{-1} NaOH + 20 mmol L^{-1} $NaNO_3$	FE = 80% YR = 93.3 µmol h^{-1} cm^{-2}	-0.3	[7]
Co_3O_4 (111) nanosheets	0.1 mol L^{-1} K_2SO_4 + 500 ppm KNO_3	FE = 99% YR = 1.17 mmol h^{-1} cm^{-2}	-0.7	[8]

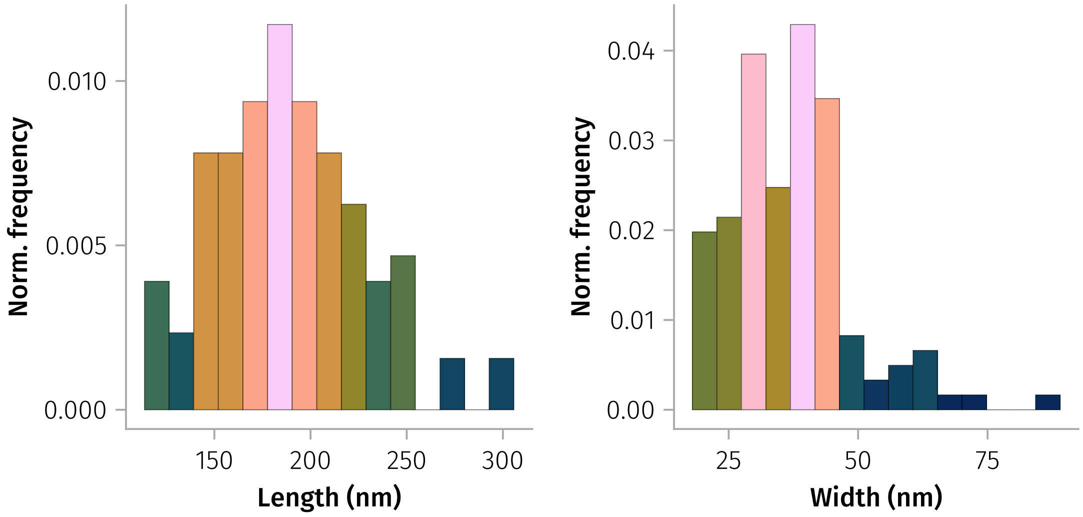

Figure S4. Length (189 ± 38 nm) and width (37 ± 12 nm) histograms of pristine Co_3O_4–np/Au measured from SEM micrographs.

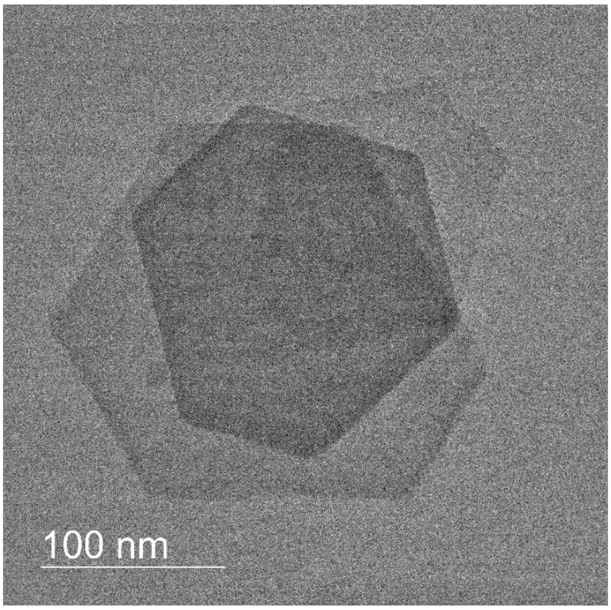

Figure S5. $Co(OH)_2$-np top-view HRTEM image.

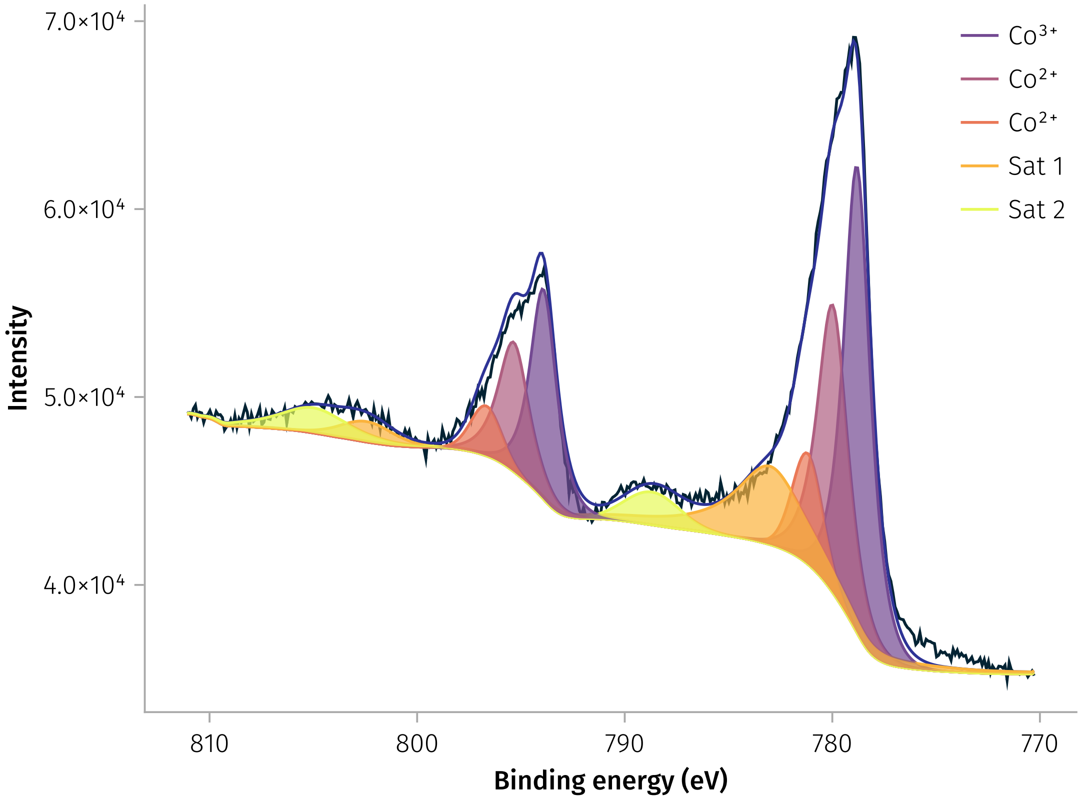

Figure S6. Deconvoluted pristine Co_3O_4–np/Au Co 2p XPS spectrum displaying both $2p_{3/2}$ and $2p_{1/2}$ bands.

Supplementary note 2. Principal component analysis and XPS peak correlation.

Many strategies are employed to better extract information from data sets of XPS spectra including peak decomposition, quantification, background analysis and statistical approaches. Principal Component Analysis (PCA) has been extensively used in the processing of data and finding the number of meaningful chemical components. A basic introduction to the technique can be found in Ref.[9]

For this work we followed the protocol developed by Evoy *et al* to examine XPS spectra using PCA algorithm.[10] A manual binding energy scale correction was done manually with CasasXPS (no significant alteration). Following, a Julia script was developed upon which the data was first normalized by area, the background (Shirley) removed and then the treated data was submitted to the multivariate procedure itself. A set of 4 XPS spectra of Co_3O_4–np/Au was taken in the following conditions; i) before any electrolysis; ii) after CA in presence of $NaNO_2$; iii) CV in the presence of $NaNO_2$ and iv) CV in the presence of $NaNO_3$. The CA and CV protocols can be found in the main text.

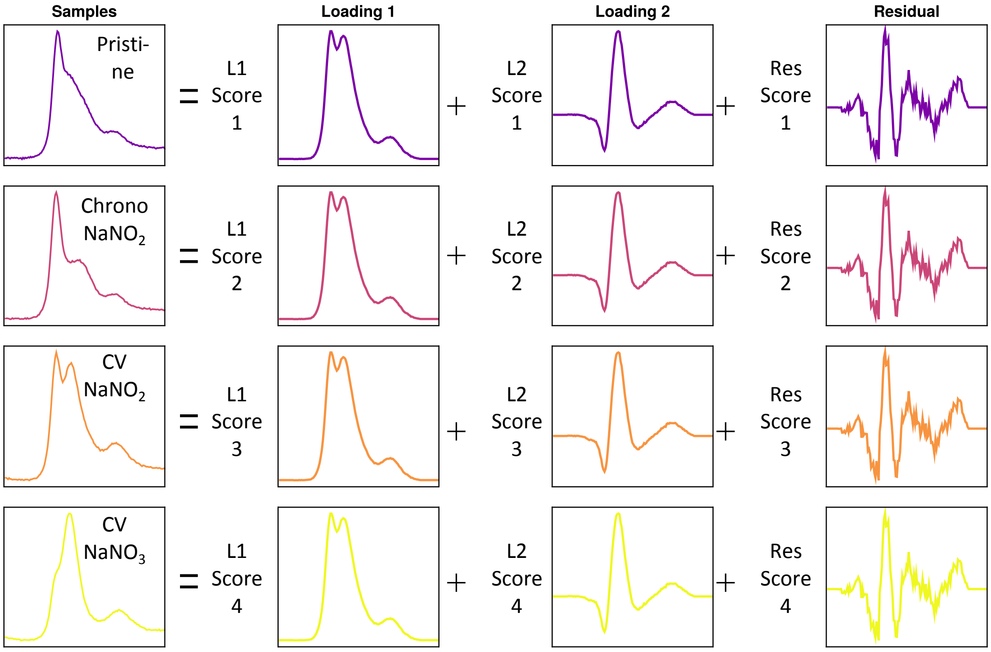

Figure S7. O 1s XPS spectra of Co_3O_4–np/Au samples after different electrochemical treatments (pristine, –0.2 V_{RHE} chrono in $NaNO_2$ 20 mmol L^{-1} + NaOH 1 mol L^{-1}, 10 cycles of CV from +0.1 to –0.6 V_{RHE} at 20 mVs^{-1} in the same electrolyte and 10 cycles of CV in the same previous conditions, but with $NaNO_3$ 20 mmol L^{-1} instead).

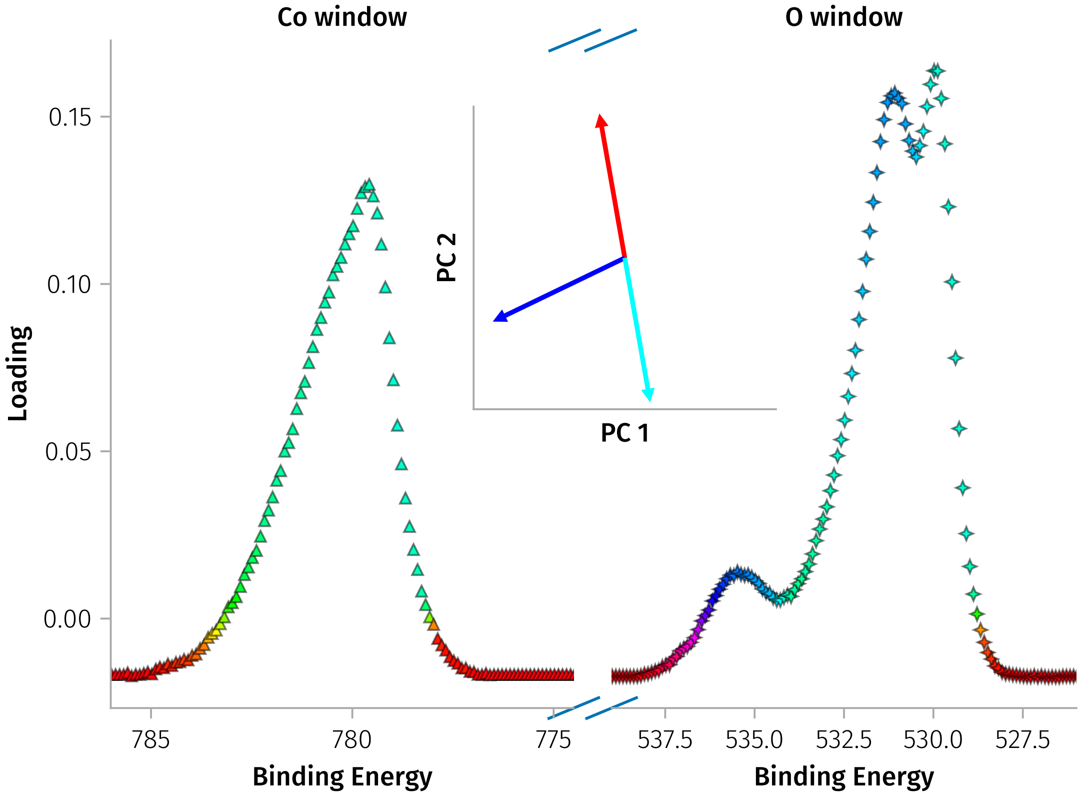

Figure S8. PCA of XPS spectra from Co_3O_4–np/Au samples. Both loading plots of O 1s and Co 2p are displayed along the corresponding correlation circle.

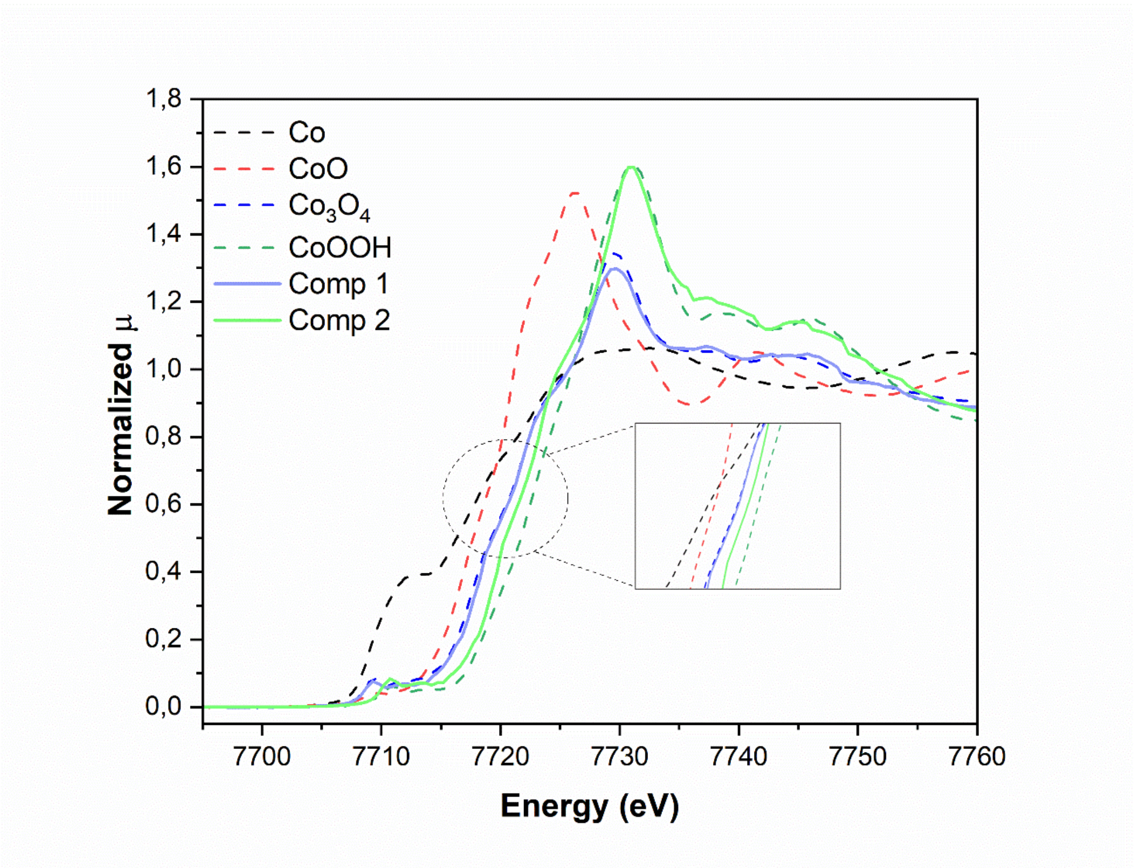

Figure S9. XANES spectra around Co_3O_4 K-edge of Co, CoO, Co_3O_4 and CoOOH samples, as well as Components 1 and 2 obtained from PCA analysis by ITFA algorithm. This procedure allows to align theoretical components obtained from PCA to physical meaningfully spectra.[11,12] The insert was taken in between the K-edge and pre-edge excitations. As can be seen Component 1 are close to Co_3O_4 and Component 2 can be identified with CoOOH, it can be established that all the peaks and valleys of both spectra in the deconvoluted ITFA Slight differences in oxidation state are visible in Component 2 related to CoOOH, probably due to a remanent Co^{2+} in the surfaces, as can be shown in the XPS analysis.

Supplementary note 3. Equivalent electrical circuit and NO_3RR mechanism.

We followed the protocol presented by Harrington to derive an equivalent circuit based on a mechanism proposal,[13] using Lasia's formalism for writing the equations.[14] For the NO_3RR, the sites where the NO intermediate remains adsorbed defines what path the reaction will follow. Either the N-, O- or both sites are simultaneously adsorbed on the electrode's interface, upon which the remaining intermediates are formed. Without loss of generality, we take the direct N site hydrogenation pathway for NO_3^- reduction to NH_3 in order to build an equivalent circuit. Ultimately, the choice of where the hydrogenation occurs won't impact in the circuit's final topology. Both NO_3^- and H- will be treated as adsorbates. The elementary reactions for said mechanism are described below, following a direct electrocatalytic reduction pathway.[15] Initially, the MNO_3^- specie is reduced to MN accordingly to electron transfer reduction steps, whereas the hydrogenation towards MNH_3 happens due to adsorbed hydrogen reduction steps, finally desorbing the product.

$$M + NO_3^- \rightleftharpoons MNO_3^- (1)$$

$$M + H_2O + e^- \rightleftharpoons MH + OH^- (2)$$

$$MNO_3^- + H_2O \ + \ 2e^- \rightleftharpoons MNO_2^- + 2OH^- (3)$$

$$MNO_2^- + H_2O \ + \ e^- \rightleftharpoons MNO + OH^- (4)$$

$$MNO + H_2O \ + \ 2e^- \rightleftharpoons MN + 2OH^- (5)$$

$$MN + MH \rightleftharpoons (6)$$

$$MNH + MH \rightleftharpoons MNH_2 (7)$$

$$MNH_2 + MH \rightleftharpoons MNH_3 (8)$$

$$MNH_3 \rightleftharpoons M + NH_3 (9)$$

In this formulation, M represents the electrode interface and all steps are reversible. The NO_3^- adsorbate is modeled within a simple adsorption reaction, whereas the H adsorption happens accordingly to a Volmer step. As stated previously in the main text, equation 3 is the rate determining step for NO_3RR. Later on, this will be important for final simplifications. From these elementary steps, it derives the following flux equations:

$$v_1 = k_1 \cdot (1 - x) - k_{-1} \cdot \theta_{NO3} (10)$$

$$v_2 = k_2 \cdot (1 - x) - k_{-2} \cdot \theta_H (11)$$

$$v_3 = k_3 \cdot \theta_{NO3} - k_{-3} \cdot \theta_{NO2}$$

$$v_4 = k_4 \cdot \theta_{NO2} - k_{-4} \cdot \theta_{NO} (13)$$

$$v_5 = k_5 \cdot \theta_{NO} - k_{-5} \cdot \theta_N (14)$$

$$v_6 = k_6 \cdot \theta_N \cdot \theta_H - k_{-6} \cdot \theta_{NH} (15)$$

$$v_7 = k_7 \cdot \theta_{NH} \cdot \theta_H - k_{-7} \cdot \theta_{NH2} (16)$$

$$v_8 = k_8 \cdot \theta_{NH2} \cdot \theta_H - k_{-8} \cdot \theta_{NH3} (17)$$

$$v_9 = k_9 \cdot \theta_{NH3} - k_{-9} \cdot (1 - x) (18)$$

Where k_i is the reaction constant with the convention that $i > 0$ refers to forward steps and i negative for backward ones. x is defined as the sum of all the intermediates surface coverage on the electrode ($x = \theta_{NO3} + \theta_{NO2} + ... + \theta_H$). A few sidenotes are worth taking. We are explicitly including reaction sites as separated species, implying no lateral interaction. This follows the Langmuir isotherm construction. Ultimately, the choice of what isotherm to use won't affect the final geometry of the equivalent circuit. For simplification we will also assume that NO_3^-, NO_2^-, OH^- and so on have fast mass transports so that their concentrations are treated as static values (encompassed into constants k_i), so that the final circuit will only account for adsorption and desorption of intermediates. With these considerations in mind the following DC equations are obtained:

$$j_f = -F \cdot (v_2 + 2v_3 + v_4 + 2v_5) = -F \cdot r_o (19)$$

$$\frac{\sigma_{NO3}}{F} \cdot \frac{d\theta_{NO3}}{dt} = v_1 - v_3 = r_1 (20)$$

$$\frac{\sigma_H}{F} \cdot \frac{d\theta_H}{dt} = v_2 - v_6 - v_7 - v_8 = r_2 (21)$$

$$\frac{\sigma_{NO2}}{F} \cdot \frac{d\theta_{NO2}}{dt} = v_3 - v_4 = r_3 (22)$$

$$\frac{\sigma_{NO}}{F} \cdot \frac{d\theta_{NO}}{dt} = v_4 - v_5 = r_4 (23)$$

$$\frac{\sigma_N}{F} \cdot \frac{d\theta_N}{dt} = v_5 - v_6 = r_5 (24)$$

$$\frac{\sigma_{NH}}{F} \cdot \frac{d\theta_{NH}}{dt} = v_6 - v_7 = r_6 (25)$$

$$\frac{\sigma_{NH2}}{F} \cdot \frac{d\theta_{NH2}}{dt} = v_7 - v_8 = r_7 (26)$$

$$\frac{\sigma_{NH3}}{F} \cdot \frac{d\theta_{NH3}}{dt} = v_8 - v_9 = r_8 (27)$$

Where j_f stands for the Faradaic current, F is the Faraday constant (96485 C mol^{-1}) and σ_x is the electrode total surface charge once completely filled with the species x. In order to find the final expression for the impedance Z, one can linearize each one of the DC equations and then apply the Fourier Transform ($\mathcal{F}$) to switch to the frequency domain, which is equivalent as treating the quantities Δj_f and so on as rotating vectors. These steps can be quickly done by making use of the following relations:

- If f(x, y, …) then $\Delta f = \frac{\partial f}{\partial x} \Delta x + \frac{\partial f}{\partial y} \Delta y + ...$ for small Δf,
- $\Delta x = \tilde{x} \, exp(j\omega t)$,
- $\frac{d\Delta x}{dt} = j\omega \tilde{x} \, exp(j\omega t)$.

Applying these procedures on equations 19 – 27 they become the following AC linearized expressions:

$$\tilde{j_f} = -F\left[\frac{\partial r_o}{\partial \eta}\cdot\tilde{\eta} + \frac{\partial r_o}{\partial \theta_{NO3}}\cdot\tilde{\theta}_{NO3} + \frac{\partial r_o}{\partial \theta_{NO2}}\cdot\tilde{\theta}_{NO2} + \dots + \frac{\partial r_o}{\partial \theta_H}\cdot\tilde{\theta}_H\right](28)$$

$$j\omega\frac{\sigma_{NO3}}{F}\cdot\tilde{\theta}_{NO3} = \left[\frac{\partial r_1}{\partial \eta}\cdot\tilde{\eta} + \frac{\partial r_1}{\partial \theta_{NO3}}\cdot\tilde{\theta}_{NO3} + \frac{\partial r_1}{\partial \theta_{NO2}}\cdot\tilde{\theta}_{NO2} + \dots + \frac{\partial r_1}{\partial \theta_H}\cdot\tilde{\theta}_H\right](29)$$

$$j\omega\frac{\sigma_H}{F}\cdot\tilde{\theta}_H = \left[\frac{\partial r_2}{\partial \eta}\cdot\tilde{\eta} + \frac{\partial r_2}{\partial \theta_{NO3}}\cdot\tilde{\theta}_{NO3} + \frac{\partial r_2}{\partial \theta_{NO2}}\cdot\tilde{\theta}_{NO2} + \dots + \frac{\partial r_2}{\partial \theta_H}\cdot\tilde{\theta}_H\right](30)$$

$$j\omega\frac{\sigma_{NO2}}{F}\cdot\tilde{\theta}_{NO2} = \left[\frac{\partial r_3}{\partial \eta}\cdot\tilde{\eta} + \frac{\partial r_3}{\partial \theta_{NO3}}\cdot\tilde{\theta}_{NO3} + \frac{\partial r_3}{\partial \theta_{NO2}}\cdot\tilde{\theta}_{NO2} + \dots + \frac{\partial r_3}{\partial \theta_H}\cdot\tilde{\theta}_H\right](31)$$

$$j\omega\frac{\sigma_{NO}}{F}\cdot\tilde{\theta}_{NO} = \left[\frac{\partial r_4}{\partial \eta}\cdot\tilde{\eta} + \frac{\partial r_4}{\partial \theta_{NO3}}\cdot\tilde{\theta}_{NO3} + \frac{\partial r_4}{\partial \theta_{NO2}}\cdot\tilde{\theta}_{NO2} + \dots + \frac{\partial r_4}{\partial \theta_H}\cdot\tilde{\theta}_H\right](32)$$

$$j\omega\frac{\sigma_N}{F}\cdot\tilde{\theta}_N = \left[\frac{\partial r_5}{\partial \eta}\cdot\tilde{\eta} + \frac{\partial r_5}{\partial \theta_{NO3}}\cdot\tilde{\theta}_{NO3} + \frac{\partial r_5}{\partial \theta_{NO2}}\cdot\tilde{\theta}_{NO2} + \dots + \frac{\partial r_5}{\partial \theta_H}\cdot\tilde{\theta}_H\right](33)$$

$$j\omega\frac{\sigma_{NH}}{F}\cdot\tilde{\theta}_{NH} = \left[\frac{\partial r_6}{\partial \eta}\cdot\tilde{\eta} + \frac{\partial r_6}{\partial \theta_{NO3}}\cdot\tilde{\theta}_{NO3} + \frac{\partial r_6}{\partial \theta_{NO2}}\cdot\tilde{\theta}_{NO2} + \dots + \frac{\partial r_6}{\partial \theta_H}\cdot\tilde{\theta}_H\right](34)$$

$$j\omega\frac{\sigma_{NH2}}{F}\cdot\tilde{\theta}_{NH2} = \left[\frac{\partial r_7}{\partial \eta}\cdot\tilde{\eta} + \frac{\partial r_7}{\partial \theta_{NO3}}\cdot\tilde{\theta}_{NO3} + \frac{\partial r_7}{\partial \theta_{NO2}}\cdot\tilde{\theta}_{NO2} + \dots + \frac{\partial r_7}{\partial \theta_H}\cdot\tilde{\theta}_H\right](35)$$

$$j\omega\frac{\sigma_{NH3}}{F}\cdot\tilde{\theta}_{NH3} = \left[\frac{\partial r_8}{\partial \eta}\cdot\tilde{\eta} + \frac{\partial r_8}{\partial \theta_{NO3}}\cdot\tilde{\theta}_{NO3} + \frac{\partial r_8}{\partial \theta_{NO2}}\cdot\tilde{\theta}_{NO2} + \dots + \frac{\partial r_8}{\partial \theta_H}\cdot\tilde{\theta}_H\right](36)$$

Clearly the final thing that is missing is to isolate the Faradaic impedance from this system of equations. This can be done by applying Kramer's rule (equation 37) to the system and solve for two 9x9 determinants.

$$Z_f = \frac{\tilde{\eta}}{\tilde{j_f}} = \frac{det(A)}{det(T)}(37)$$

$$A = \begin{bmatrix} \frac{1}{F} & \frac{\partial r_o}{\partial \theta_{NO3}} & \cdots & \frac{\partial r_o}{\partial \theta_{NH3}} & \frac{\partial r_o}{\partial \theta_H} \\ 0 & \frac{\partial r_1}{\partial \theta_{NO3}} - j\omega\frac{\sigma_{NO3}}{F} & \dots & \frac{\partial r_1}{\partial \theta_{NH3}} & \frac{\partial r_1}{\partial \theta_H} \\ \vdots & \vdots & \ddots & \vdots & \vdots \\ 0 & \frac{\partial r_7}{\partial \theta_{NO3}} & \dots & \frac{\partial r_7}{\partial \theta_{NH3}} - j\omega\frac{\sigma_{NH3}}{F} & \frac{\partial r_7}{\partial \theta_H} \\ 0 & \frac{\partial r_8}{\partial \theta_{NO3}} & \cdots & \frac{\partial r_8}{\partial \theta_{NH3}} & \frac{\partial r_8}{\partial \theta_H} - j\omega\frac{\sigma_H}{F} \end{bmatrix}(38)$$

$$T = \begin{bmatrix} -\frac{\partial r_o}{\partial \eta} & \frac{\partial r_o}{\partial \theta_{NO3}} & \cdots & \frac{\partial r_o}{\partial \theta_{NH3}} & \frac{\partial r_o}{\partial \theta_H} \\ -\frac{\partial r_1}{\partial \eta} & \frac{\partial r_1}{\partial \theta_{NO3}} - j\omega\frac{\sigma_{NO3}}{F} & \cdots & \frac{\partial r_1}{\partial \theta_{NH3}} & \frac{\partial r_1}{\partial \theta_H} \\ \vdots & \vdots & \ddots & \vdots & \vdots \\ -\frac{\partial r_7}{\partial \eta} & \frac{\partial r_7}{\partial \theta_{NO3}} & \cdots & \frac{\partial r_7}{\partial \theta_{NH3}} - j\omega\frac{\sigma_{NH3}}{F} & \frac{\partial r_7}{\partial \theta_H} \\ -\frac{\partial r_8}{\partial \eta} & \frac{\partial r_8}{\partial \theta_{NO3}} & \cdots & \frac{\partial r_8}{\partial \theta_{NH3}} & \frac{\partial r_8}{\partial \theta_H} - j\omega\frac{\sigma_H}{F} \end{bmatrix} \quad (39)$$

Since the NO_3^- to NO_2^- reduction is the rate determining step of the mechanism, all steps that occur after it won't be resolved in the EIS spectrum. Even though this mechanism proposal reaction predicts up to 8 adsorbates, only the NO_3^- and H may be detected in the resulting spectrum. This allows for the final simplification:

$$Z_f = \frac{\begin{pmatrix} \frac{1}{F} & \frac{\partial r_o}{\partial \theta_{NO3}} & \frac{\partial r_o}{\partial \theta_H} \\ 0 & \frac{\partial r_1}{\partial \theta_{NO3}} - j\omega\frac{\sigma_{NO3}}{F} & \frac{\partial r_1}{\partial \theta_H} \\ 0 & \frac{\partial r_2}{\partial \theta_H} & \frac{\partial r_2}{\partial \theta_H} - j\omega\frac{\sigma_H}{F} \end{pmatrix}}{\begin{pmatrix} -\frac{\partial r_o}{\partial \eta} & \frac{\partial r_o}{\partial \theta_{NO3}} & \frac{\partial r_o}{\partial \theta_H} \\ -\frac{\partial r_1}{\partial \eta} & \frac{\partial r_1}{\partial \theta_{NO3}} - j\omega\frac{\sigma_{NO3}}{F} & \frac{\partial r_1}{\partial \theta_H} \\ -\frac{\partial r_2}{\partial \eta} & \frac{\partial r_2}{\partial \theta_H} & \frac{\partial r_2}{\partial \theta_H} - j\omega\frac{\sigma_H}{F} \end{pmatrix}} \quad (40)$$

Which in turn can be further rearranged into:

$$Z_f = R_{CT} + \frac{\dfrac{R_{P1} + \dfrac{R_{P2}}{1 + j\omega C_{P2} R_{P2}}}{1 + j\omega C_{P1}\left(R_{P1} + \dfrac{R_{P2}}{1 + j\omega C_{P2} R_{P2}}\right)}}{1 + j\omega C_{P1}\left(\dfrac{R_{P1} + \dfrac{R_{P2}}{1 + j\omega C_{P2} R_{P3}}}{1 + j\omega C_{P1}\left(\dfrac{R_{P1} + \dfrac{R_{P2}}{1 + j\omega C_{P2} R_{P2}}}{1 + j\omega C_{P1}\left(R_{P1} + \dfrac{R_{P2}}{1 + j\omega C_{P2} R_{P2}}\right)}\right)}\right)} \quad (41)$$

Equation 41 is precisely the Faradaic impedance expression corresponding to the equivalent circuit proposed in the main text. The total impedance is obtained by taking the parallel combination of said Z_f with the double-layer capacitance C_{DL}, and then

placing this combination in series to the solution resistance R_S. This derivation does not explicitly consider the flux terms ν. Alternatively, one could reformulate Z_f using conventional kinetic models, such as Tafel or Marcus formalism, as well as incorporate different isotherm models, *e.g.*, Freundlich, BET, Temkin or Redlich–Peterson.[16] While these choices influence the numerical values of rate constants, they do not alter the circuit topology or the overall Nyquist/Bode profiles. These considerations should be accounted for when analyzing EIS data in light of the system kinetics, rather than relying solely on a circuit-based interpretation. The key implication is that the circuit topology is governed by the connectivity between elements, not by the specific kinetic dependance of each element. Given the rate-determining step simplification, the final impedance expression (written in terms of electrical circuit elements) becomes insensitive to the site where NO hydrogenation occurs, as this step happens after the NO_3^- reduction to NO_2^-. Our derivation, which stopped at the equivalent circuit result, would yield the same answer if we instead chose to use the NH_2OH route formation. The circuit fitting results across all Faradaic potentials are displayed down below.

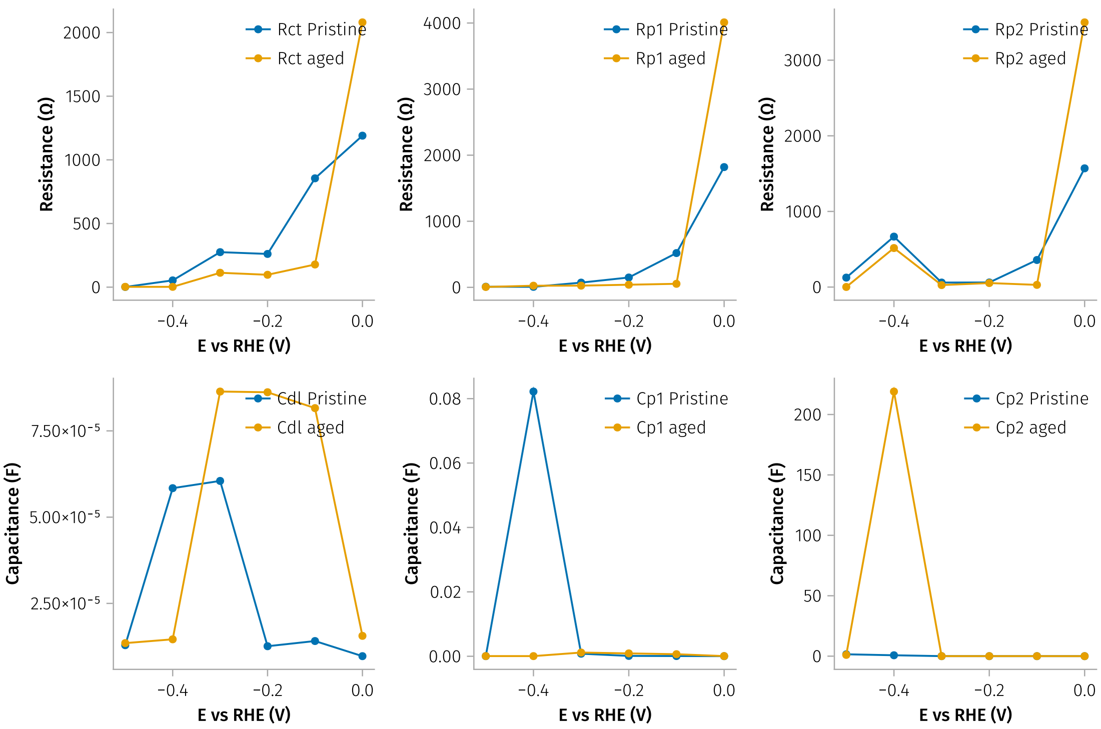

Figure S10. Circuit fit results of EIS data taken across Faradaic potentials for both pristine and aged Co_3O_4–np/GC.

Table S2. Pristine Co_3O_4-np/GC EIS circuit fitting parameters across Faradaic potentials.

Potential (V_{RHE})	Rs (*Ω*)	Cdl (F)	Rct (*Ω*)	Cp1 (F)	Rp1 (*Ω*)	Cp2 (F)	Rp2 (*Ω*)
0.0	2.07E+01	9.72E-06	1.19E+03	3.91E-05	1.82E+03	2.24E-05	1.57E+03
−0.1	1.25E+01	1.41E-05	8.55E+02	6.91E-05	5.19E+02	2.78E-03	3.58E+02
−0.2	7.82	1.26E-05	2.61E+02	1.12E-04	1.49E+02	5.39E-03	6.09E+01
−0.3	7.53	6.05E-05	2.75E+02	8.01E-04	7.15E+01	1.79E-02	5.97E+01
−0.4	6.14	5.84E-05	5.33E+01	8.22E-02	7.57E+00	7.33E-01	6.67E+02
−0.5	6.05	1.29E-05	1.52E+00	4.86E-05	9.28E+00	1.54E+00	1.25E+02

Table S3. Aged Co_3O_4-np/GC EIS circuit fitting parameters across Faradaic potentials.

Potential (V_{RHE})	Rs (*Ω*)	Cdl (F)	Rct (*Ω*)	Cp1 (F)	Rp1 (*Ω*)	Cp2 (F)	Rp2 (*Ω*)
0.0	3.99E+01	1.56E-05	2.08E+03	5.96E-05	4.01E+03	4.69E-04	3.50E+03
−0.1	6.24	8.16E-05	1.78E+02	6.41E-04	5.36E+01	2.24E-02	2.90E+01
−0.2	5.93	8.62E-05	9.72E+01	8.93E-04	3.98E+01	2.14E-02	5.31E+01
−0.3	6.79	8.64E-05	1.13E+02	1.16E-03	2.59E+01	2.41E-02	2.64E+01
−0.4	5.67	1.46E-05	2.42E+00	5.28E-05	2.48E+01	2.19E+02	5.16E+02
−0.5	6.45	1.35E-05	2.57E+00	5.93E-05	5.79E+00	1.05E+00	1.35E+00

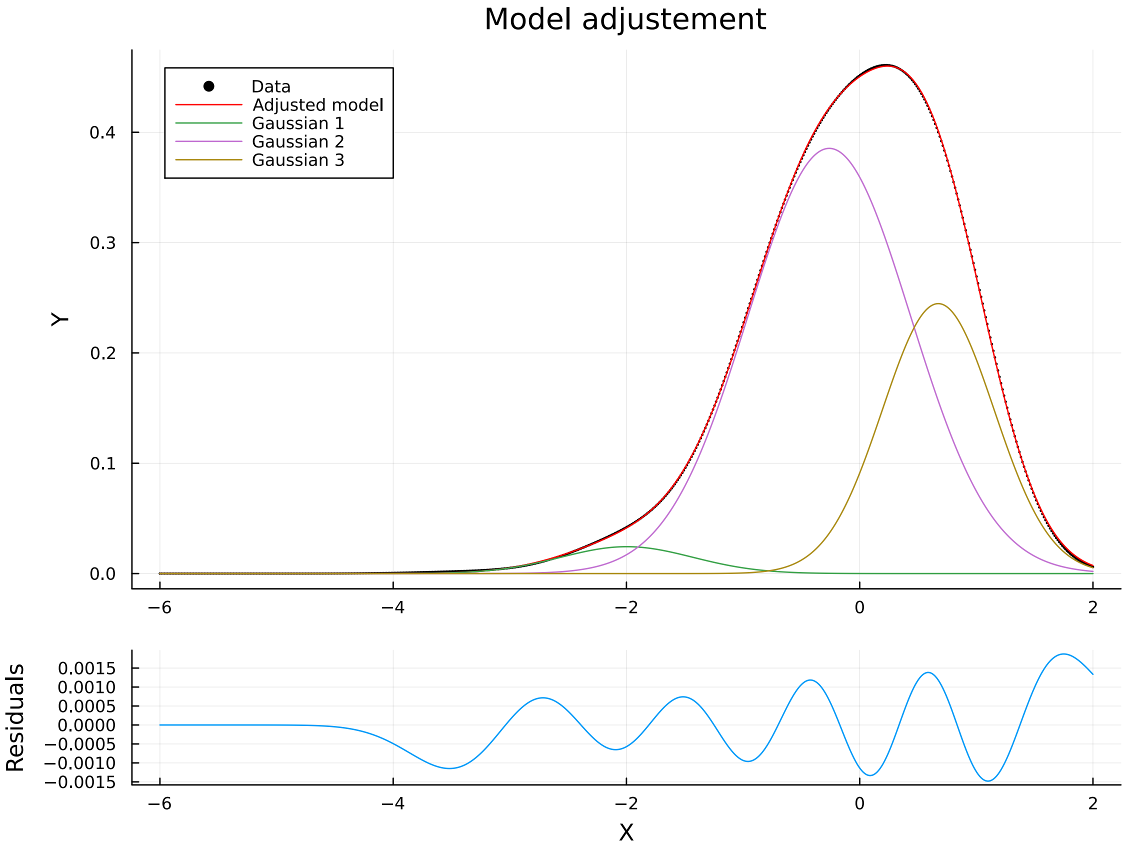

Figure S11. Deconvoluted DRT spectrum of aged Co_3O_4–np/GC at 0 V vs RHE. Deconvolution performed with the Julia package Spectra.[17]

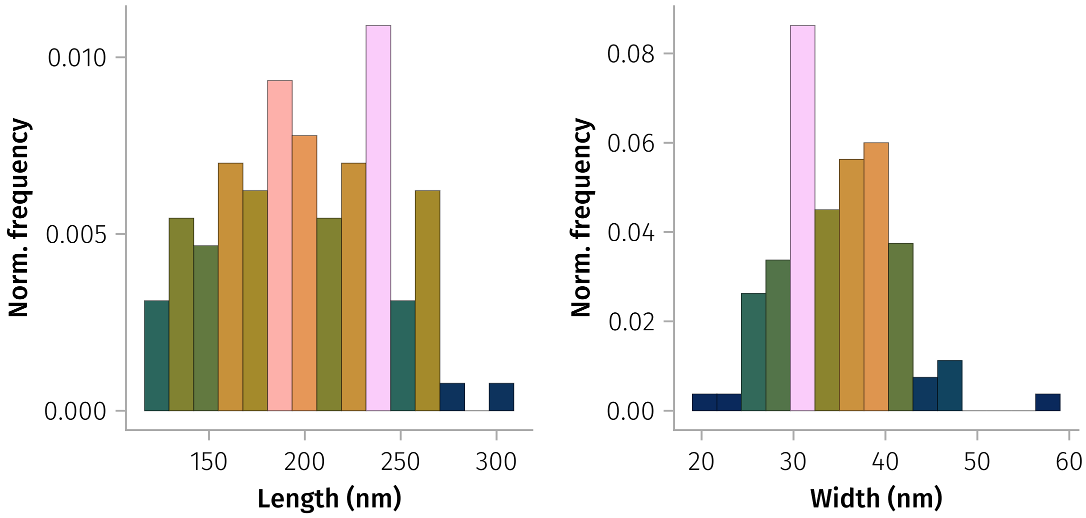

Figure S12. Length (199 ± 43 nm) and width (35 ± 6 nm) histograms of aged Co_3O_4–np/Au measured from SEM micrographs.

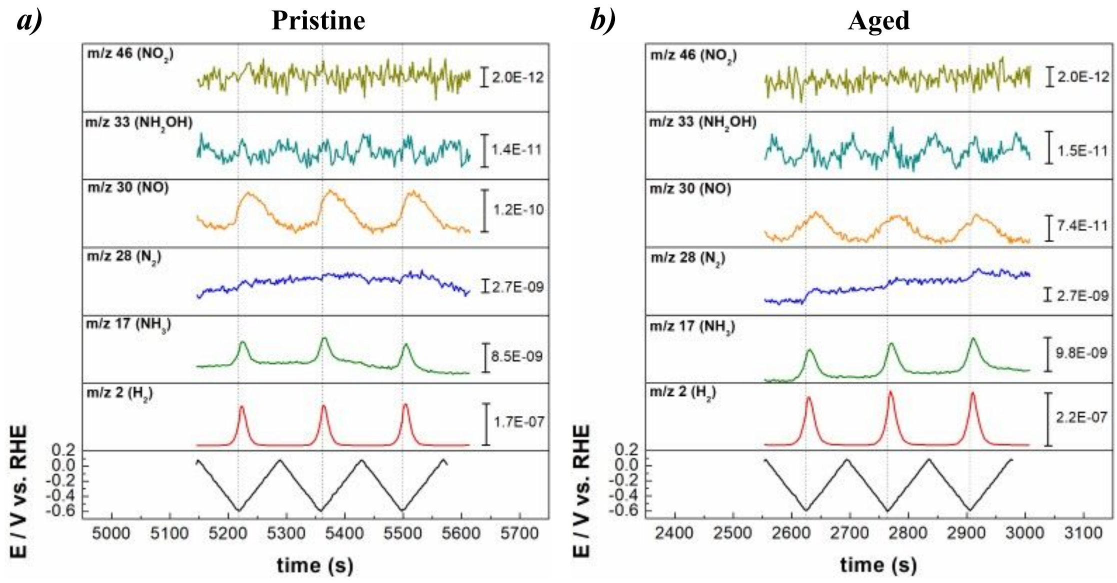

Figure S13. Pristine and aged Co_3O_4-np ionic fragments (H_2^+, NH_2OH^+, NO^+, N_2^+, NH_3^+) measured by EC-MS following CV protocol.

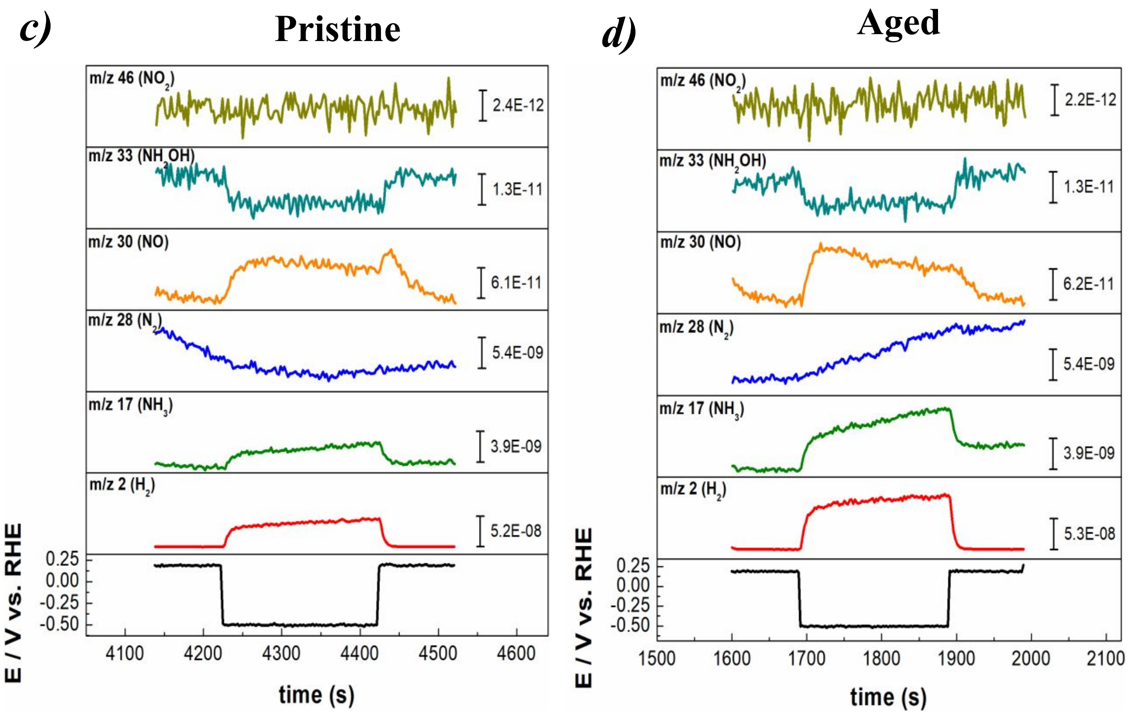

Figure S14. Pristine and aged Co_3O_4-np ionic fragments (H_2^+, NH_2OH, NO, N_2, NH_3, H_2) measured by EC-MS following CA protocol.

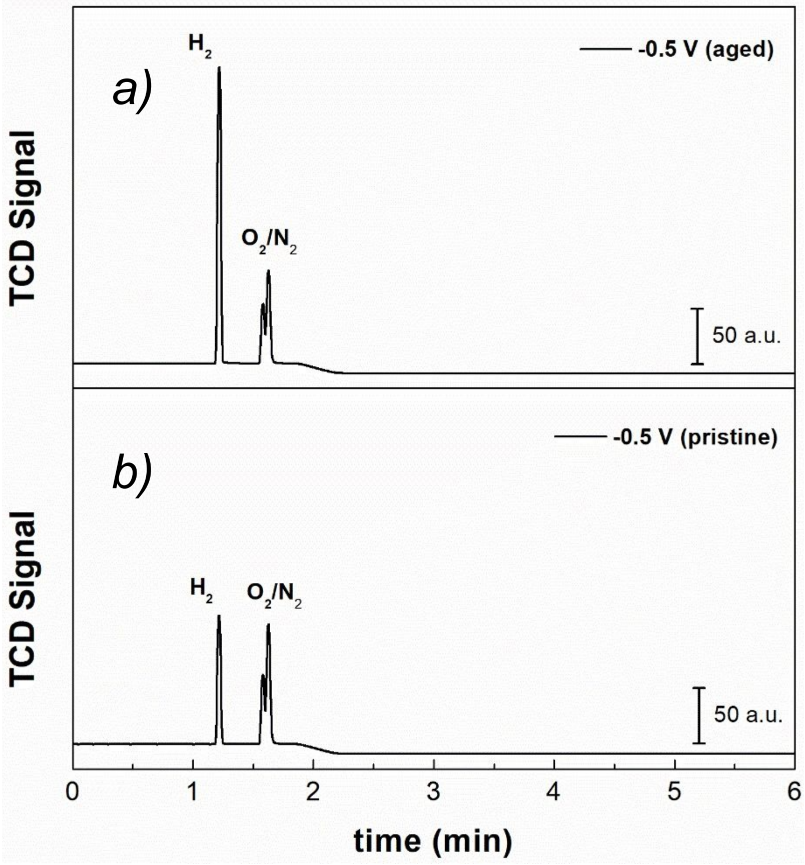

Figure S15. H_2, N_2 and O_2 chromatogram signals of Co_3O_4-np post-electrolysis headspace, before and after aging protocol, registered by a TCD detector. The N_2 and O_2 signals observed in the TCD are attributed to trace amounts of ambient air introduced into the needle dead volume during gas sampling and injection. The syringe's full gas-tightness is preserved.

Supplementary note 4. Electronic supporting information

This section presents supplementary, in-depth information on the computational investigation of spinel Co_3O_4 as a catalytic support for the nitrate reduction reaction (NO_3RR). It provides a systematic account of the energetic data obtained from first-principles calculations, together with the algebraic formalisms and structural models employed to derive the key parameters required to characterize the energetic, geometric, and electronic properties of the system. Particular attention is devoted to elucidating the impact of distinct cobalt–oxygen stoichiometries within the lattice, with an emphasis on how these compositional variations modulate the reactivity of specific Co_3O_4 surface terminations. In addition, the document develops a theoretical framework for quantifying the thermodynamic contributions to the NO_3RR, thereby facilitating a more rigorous interpretation of the observed catalytic performance trends across different substrate environments.

S-1 Additional computational detail

All first-principles calculations in this work were carried out using the Vienna Ab initio Simulation Package (VASP). The characteristics of the employed Projector Augmented Wave (PAW) datasets, used as POTCAR files, are compiled in Table S4.[18,19]

Table S4. Specific technical parameters of the PAW-PBE projectors employed in this work, including the recommended plane-wave cutoff energy, ENMAX (eV), the number of valence electrons ($Zval$), and the corresponding valence electronic configuration.

Element	PAW projector	ENMAX	Z_{val}	Electronic Configuration
O	O_GW 19Mar2012	434.431	6	$[He]2s^22p^4$
Co	O_GW 19Mar2012	323.400	9	$[Ar]4s^23d^7$
N	N_GW_new 19Mar2012	420.902	5	$1s^22s^22p^3$
H	H_GW 21Apr2008	300.000	1	$1s^1$

S-2 Physical chemical properties

S-2.1 Geometric parameters

The molecular systems H_2, NO_2, $trans$-HONO, H_2O, and HNO_3 are described in terms of their interatomic distances, $d\ i{-}j$, defined between atoms i and j, and their molecular bond angles, α^{mol}. For molecules comprising more than three atoms, the molecular bond angle α^{mol} is more specifically denoted as $\tilde{i}$, where i designates the central atom of the angle, as depicted in Figure S16.

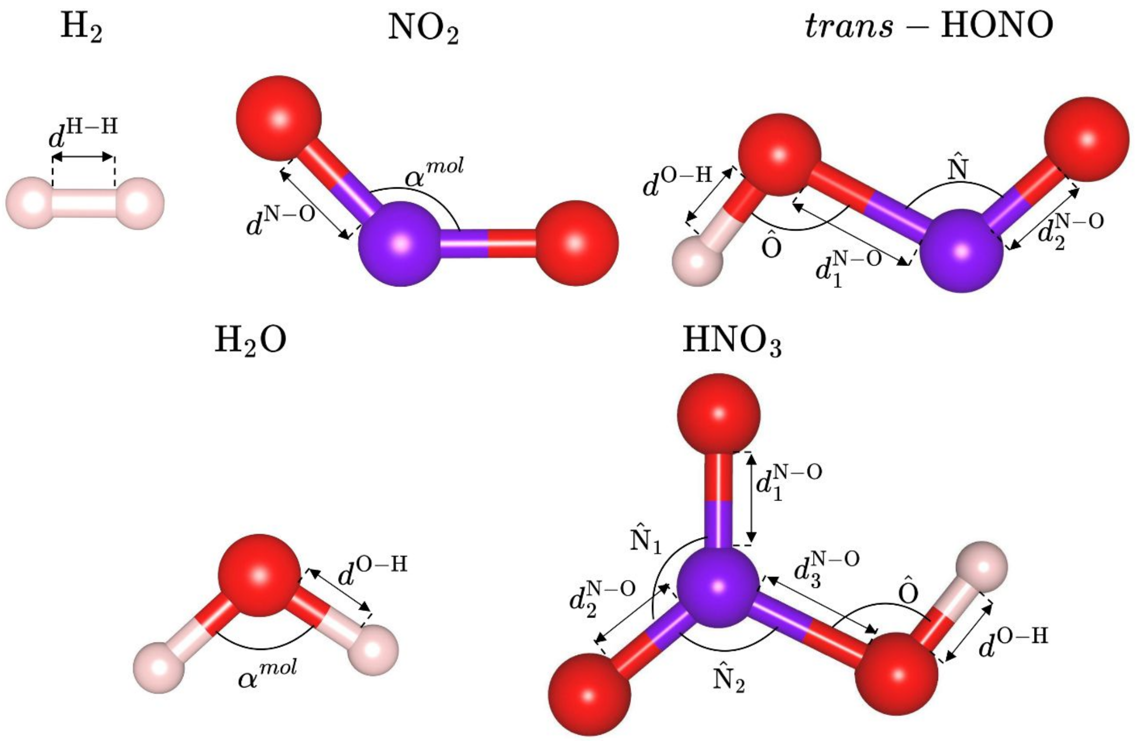

Figure S16. Geometric parameters of the molecular species involved in the NO_3RR process. Light pink, purple, and red spheres denote hydrogen, nitrogen, and oxygen atoms, respectively, thereby highlighting the atomic composition and bonding environment.

The adsorption of molecular species on Co_3O_4(111) surfaces is characterized by analyzing bond angles and interatomic distances between surface sites and the adsorbates, as depicted in Figure S17. Furthermore, the intrinsic geometric parameters of the isolated molecules are examined, and their implications will be discussed in detail in the Results section.

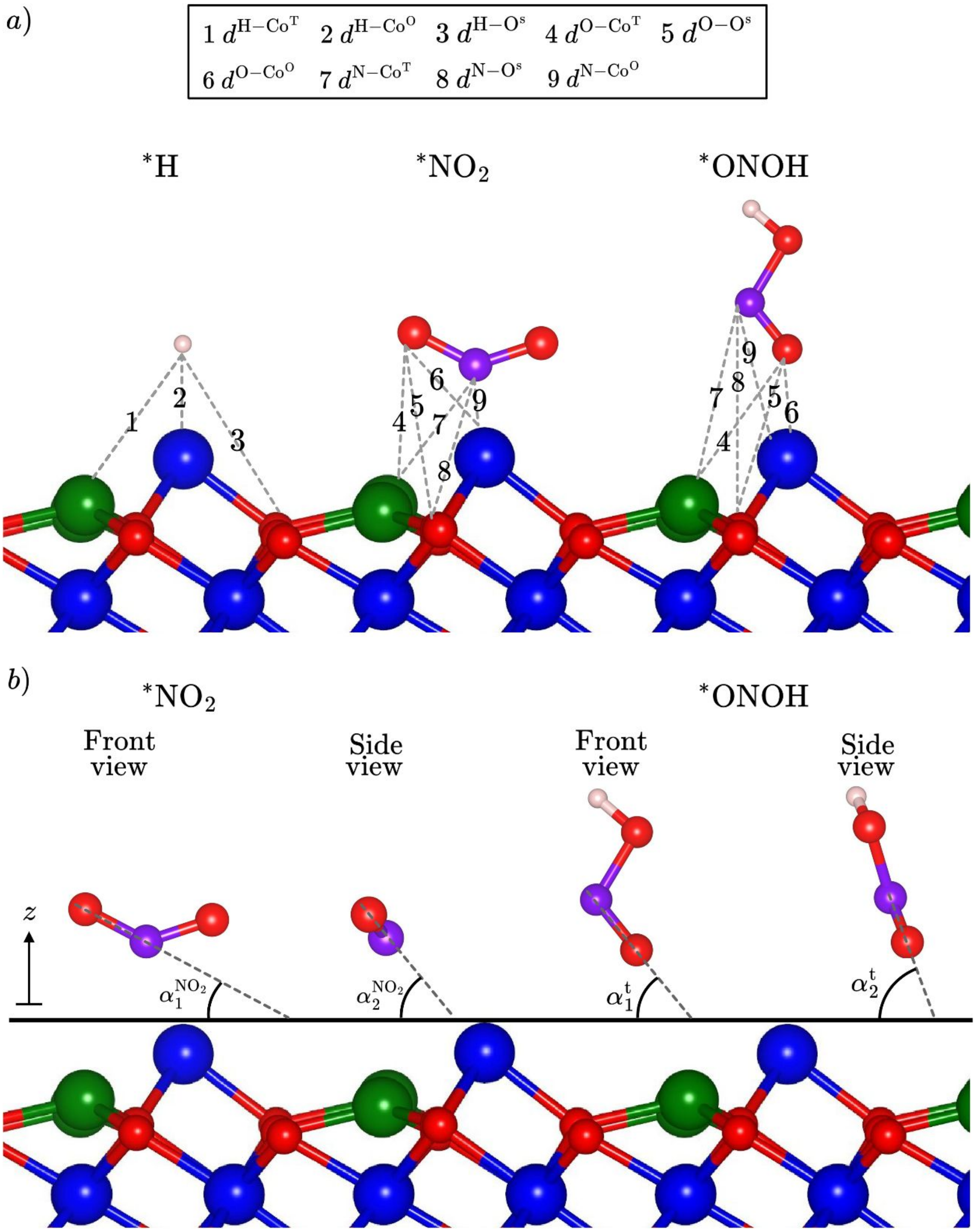

Figure S17. Geometric parameters of molecules adsorbed on surfaces, illustrated by: a) interatomic distances between molecular atoms and surface sites, and b) molecular orientation angles relative to the plane parallel to the z-axis of the supercell (highlighted by the horizontal black line). Green, blue, red, and light-pink spheres represent tetrahedral Co, octahedral Co, oxygen, and hydrogen atoms, respectively.

S-2.2 Molecular binding energy

For each molecular species i, the corresponding total DFT energy E^{i}_{tot} is evaluated. The binding energy is then defined as the difference between the total energy of the molecule and the sum of the total energies of its isolated constituent atoms, all

calculated in the gas phase while maintaining the correct stoichiometric ratios. Accordingly, for the molecular systems examined in this work, we obtain

$$E_b^{H_2} = E_{tot}^{H_2} - 2 \cdot E_{tot}^{H} (42)$$

$$E_b^{NO_2} = E_{tot}^{NO_2} - E_{tot}^{N} - 2 \cdot E_{tot}^{O} (43)$$

$$E_b^{trans-HONO} = E_{tot}^{trans-HONO} - E_{tot}^{N} - E_{tot}^{H} - 2 \cdot E_{tot}^{O} (44)$$

$$E_b^{H_2O} = E_{tot}^{H_2O} - 2 \cdot E_{tot}^{H} - E_{tot}^{O} (45)$$

$$E_b^{HNO_3} = E_{tot}^{HNO_3} - E_{tot}^{N} - E_{tot}^{H} - 3 \cdot E_{tot}^{O} (46)$$

S-2.3 Cohesive energy

The thermodynamic stability of the system can be characterized by the cohesive energy (E_{coh}), which is defined as

$$E_{coh} = \frac{E_{tot}^{Co_3O_4} - 6 \cdot E_{tot}^{Co} - 8E_{tot}^{O}}{14} (47)$$

In this expression, $E_{tot}^{Co_3O_4}$ represents the total energy of bulk spinel Co_3O_4, whereas E_{tot}^{Co} and E_{tot}^{O} denote the total energies of isolated Co and O atoms in the gas phase, respectively. The denominator, 14, corresponds to the total number of atoms in the primitive unit cell of Co_3O_4.

S-2.4 Surface energy

To quantify the energetic cost associated with the formation of Co_3O_4(111) spinel surfaces within slab models, it is necessary to evaluate $E^{surf}{}_{tot}$ and E^{bulk}, corresponding to the total DFT energies of the surface and of the bulk (per atom), respectively. The surface energy, σ_s, is then calculated as

$$\sigma_s = \frac{E_{tot}^{surf} + k \cdot E_{tot}^{Co(hcp)} - N_{bulk} \cdot E_{tot}^{bulk}}{2A} (48)$$

where N_{bulk} denotes the number of atoms in the slab that are equivalent to those in the bulk, and A is the area of a single surface facet.

Because this study includes surfaces with different Co/O stoichiometries, the total DFT energy of primitive hcp cobalt, $E_{tot}^{Co(hcp)}$, which represents the thermodynamically stable phase under ambient conditions,[20] is used as a reference to consistently account for composition and preserve the proportionality of total energy differences. In this framework, the correction factor k takes the values 0 for stoichiometric surfaces, −2 for reduced surfaces, and 2 for oxidized surfaces. These values reflect that the reduced and oxidized slabs contain four fewer or four additional cobalt atoms than the stoichiometric model, respectively, whereas the unit cell hcp Co contains two atoms. This normalization procedure enables a consistent energetic comparison among surfaces with different oxidation states, removing compositional artifacts that might otherwise mask intrinsic thermodynamic behavior. Consequently, the resulting analysis provides a rigorous and

physically grounded basis for evaluating the stability of cobalt oxide terminations under varying redox conditions.

S-2.5 Adsorption energy

The adsorption energy (ΔE_{ad}) can be calculated using the total DFT energies of the pristine substrate (E_{tot}^{subs}), the isolated molecule in the gas phase (E_{tot}^{mol}), and the combined system consisting of the substrate with the adsorbed molecule ($E_{tot}^{mol/subs}$), as expressed in Eq. 49.

$$\Delta E_{ad} = E_{tot}^{mol/subs} - E_{tot}^{mol} - E_{tot}^{subs} (49)$$

S-2.6 Bader charge

Elucidating the relationship between the intrinsic electronic structure and the measured electrochemical performance is essential for rationalizing catalytic trends. Because the thermodynamics of the reaction are fundamentally governed by the capacity of the active site to donate or accept electrons, we employed a topology-based Bader charge analysis to quantify the local charge redistribution induced upon adsorption.[21] This formalism partitions the continuous total electron density into discrete atomic basins, which are delineated by zero-flux surfaces in the gradient field of the charge density. The effective partial charge (Q_{eff}^{i}) associated with a given atom i is defined as

$$Q_{eff}^{i} = Z_{val} - Q_{Bader}^{i} (50)$$

Here, Z_{val} denotes the number of valence electrons, whereas Q_{Bader}^{i} corresponds to the integrated electron population within the Bader volume assigned to atom i. Accordingly, a negative value of Q_{eff}^{i} indicates a net accumulation of electron density, while a positive value reflects net charge depletion. Although these computed partial charges do not rigorously correspond to formal oxidation states, they provide a robust and quantitative descriptor for tracking local charge-transfer processes between the catalytic surface and the reaction intermediates.[22]

S-2.7 Gibbs free energy

The Gibbs free energy (G) is evaluated by the following equation,

$$G = E_{tot} + ZPE + \int C_p dT - (51)$$

The correction terms, zero-point energy (ZPE), the enthalpic correction ($\int Cp\ dT$), and the entropic contribution ($-TS$), were determined from vibrational frequency analysis performed within the harmonic oscillator approximation at 298.15 K, using the thermochemistry utilities implemented in the Atomic Simulation Environment (ASE).[23] These contributions are expressed as:

$$ZPE = \sum_{i} \frac{1}{2} \epsilon_i (52)$$

$$\int C_p\, dT = \sum_i \frac{\epsilon_i}{exp\left(\frac{\epsilon_i}{k_B T}\right) - 1} (53)$$

$$S = k_B \sum_i \left[\frac{\epsilon_i}{k_B T(exp\left(\frac{\epsilon_i}{k_B T}\right) - 1)} - ln\left(1 - exp\left(-\frac{\epsilon_i}{k_B T}\right)\right) \right] (54)$$

In these expressions, ϵ_i denotes the energy of the i-th vibrational mode, while k_B and T represent the Boltzmann constant and the absolute temperature, respectively.

On the basis of these thermodynamic quantities, a key relative measure can be defined. The Gibbs free energy of adsorption (ΔG_{ad}) quantifies the difference in free energy between the adsorbed state ($G_{tot}^{mol/subs}$) and the sum of the free energies of the isolated molecule (G_{tot}^{mol}) and the clean substrate (G_{tot}^{subs}). This quantity serves as a rigorous descriptor for evaluating the thermodynamic favorability of adsorption processes:

$$\Delta G_{ad} = G_{tot}^{mol/subs} - G_{tot}^{subs} - G_{tot}^{mol} (55)$$

Within the approximation that the vibrational spectrum of the substrate is only weakly perturbed by molecular adsorption, $G_{tot}^{mol/subs}$, evaluated by constraining the positions of the substrate atoms during vibrational analysis. Under this assumption, G_{tot}^{subs} can be approximated by its electronic contribution E_{tot}^{subs}, which leads to a simplified expression for the free energy of adsorption:

$$\Delta G_{ad} = G_{tot}^{mol/subs} - E_{tot}^{subs} - G_{tot}^{mol} (56)$$

S-3 THEORETICAL APPROACH

S-3.1 Total energy calculations

Spin-polarized density functional theory (DFT) calculations were performed using the Vienna Ab initio Simulation Package (VASP), version 5.4.4,[18,19] employing the projector augmented-wave (PAW) method[19,24] to describe the interaction between the core and valence electrons. The exchange–correlation energy was treated within the semilocal Perdew–Burke–Ernzerhof (PBE) generalized gradient approximation.[25] However, conventional DFT-PBE is known to inadequately describe the strongly localized d-electron states of Co. To obtain a more accurate and physically consistent description of these states, we adopted the rotationally invariant PBE+U scheme proposed by Dudarev,[26] using an effective Hubbard parameter $U_{\text{eff}} = 3.50$ eV for Co, in line with previous studies.[27,28] In addition, long-range van der Waals interactions were taken into account via the empirical Grimme D3 dispersion correction,[29] which is crucial for accurately predicting structural properties and obtaining reliably corrected binding energies.[30–32]

The equilibrium lattice constant of bulk spinel Co_3O_4, which crystallizes in the $D7_2$ structure prototype and belongs to the $Fd\bar{3}m$ space group, was determined by means

of full structural relaxations, involving the concurrent minimization of both atomic forces and the stress tensor.[33] To guarantee reliable convergence of the total energies, a plane-wave kinetic-energy cutoff of 868.862 eV was adopted, corresponding to twice the largest recommended cutoff among the constituent elements (oxygen) specified in the POTCAR files, thereby improving the accuracy and numerical robustness of the calculations. The initial magnetic configuration was set according to an antiferromagnetic alignment of the tetrahedrally coordinated Co^{2+} ions, while the octahedrally coordinated Co^{3+} sites were treated as diamagnetic.[34] Brillouin-zone sampling was performed using Monkhorst–Pack[35] k-point meshes of 4×4×4 for the primitive cell of Co_3O_4. The resulting equilibrium lattice parameter obtained in this work is 8.09 Å, in excellent agreement with the experimental value of 8.0835 Å[36] and closely matching previous DFT+U results of 8.09 Å,[37] corresponding to a relative deviation less than 0.9 %, i.e., an excellent concordance.

All calculations involving both pristine slabs and the corresponding adsorbate–slab systems were performed using a plane-wave kinetic energy cutoff of 488.734 eV. This value exceeds the largest ENMAX parameter among all elements considered in this study by 12.5 %, ensuring a systematically converged and robust base set across all simulations. Geometry optimizations were initially conducted at the Γ-point, and subsequent single-point energy calculations were performed on a (3×3×1) k-point mesh to obtain accurate total energies. The slab models were constructed with a vacuum spacing of 15 Å to suppress spurious interactions between periodic images, and a (1×1) surface unit cell was employed.

The vibrational frequencies for all adsorbed species were determined via finite-difference calculations based on the numerical evaluation of second-order energy derivatives. Gas-phase molecules were modeled in an orthorhombic simulation cell of dimensions 20.00 Å×20.25 Å×20.50 Å using a Γ-point sampling scheme (1×1×1), which effectively suppresses spurious dispersion interactions arising from periodic images. The convergence criterion was set to 10^{-6} eV for the minimization of total-energy and 0.01 eV $Å^{-1}$ for the maximum residual force on each atom, thus ensuring the robustness and precision of the calculated vibrational properties.

S-3.2 Computational hydrogen electrode model

The approach employed for DFT simulations, which has proven highly successful, is the Computational Hydrogen Electrode (CHE) model.[38] This framework enables electronic and protonic energies to be directly related to the actual chemical potential in electrochemical cell simulations. The reference reaction is the following,

$$H^+ + e^- \rightleftarrows \frac{1}{2} H_2 (57)$$

which establishes equivalence in both chemical potential and energy. Consequently, the energy of gaseous H_2 is used as a reference for protons and electrons, providing a robust basis for calculating theoretical potentials in electrochemical reactions. Reactions in which an electron and a proton are transferred simultaneously are termed proton-coupled electron transfer (PCET). Accordingly, the general reaction $*A + H^+ + e^- \rightarrow *AH$ can

be reformulated within the CHE framework as $*A+\frac{1}{2}H_2\rightarrow *AH$, where the asterisk denotes the active site on the substrate.

S-3.3 NO_3RR mechanism

The nitrate reduction reaction, NO_3RR, involves the electrochemical reduction of nitrate (NO_3^-) in the cathode during electrolysis. The simplified mechanism, which highlights the branching point between the formation of NO_2^- and the continuation of the PCET pathways to the various products of NO_3RR, is illustrated in Figure S18-a. In an aqueous solution, the fundamental reactions are expressed as

$$*NO_2\rightarrow NO_{2(aq)}^- + \ *$$

and the alternative pathway

$$*NO_2+H^+ + e^-\rightarrow * \ (59)$$

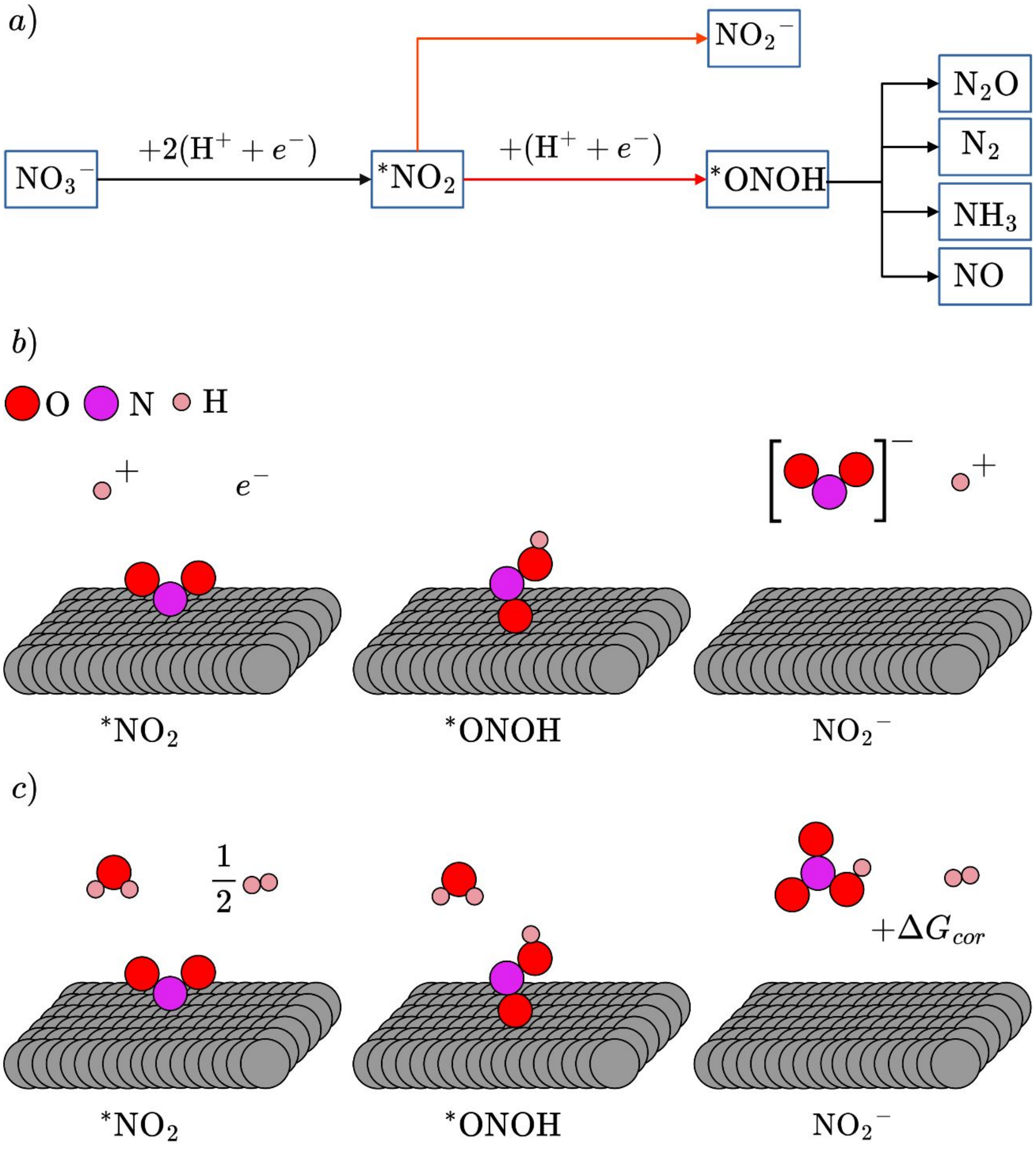

Figure S18. a) Simplified mechanism illustrating the pathway from the nitrate to the possible products, with red arrows indicating the specific routes considered in the simulation of nitrite formation. b) Snapshots of the theoretical components in a closed system, used for the DFT calculations within the CHE framework. c) The feasible substitution of components that are actually included in the computational evaluation. Dark grey spheres represent surface atoms.

To bypass the direct treatment of charged species $NO_2^-{}_{(aq)}$, one can use the following sequence of steps:

$$HNO_{3(g)} \rightarrow HNO_{3(l)}, \qquad \Delta G_{c1} (60)$$

$$HNO_{3(l)} + \frac{1}{2} H_{2(g)} \rightarrow H_2O_{(l)} + NO_{2(aq)}^-, \qquad \Delta G_{c2} (61)$$

$$H_2O_{(l)} \rightarrow H_2O_{(g)}, \qquad \Delta G_{c3} (62)$$

The reactions exhibit empirically determined ΔG values of ΔG_{c1} = −0.075 eV, ΔG_{c2} = −1.955 eV, and ΔG_{c3} = 0.088 eV, each determined under canonical thermodynamic conditions. Specifically, the Gibbs free energies were assessed at T = 298.15 K and p = 101.325 Pa.[39]

Thus, considering the eliminations in these reactions and the equilibrium established in Eq. 57, the adsorption free energy of $*NO_2$ is given by

$$\Delta G_{ad}^{*NO_2} = G_{tot}^{*NO_2} + G_{tot}^{H_2O} - G_{tot}^{HNO_3} - \frac{1}{2} G_{tot}^{H_2} - E^{*}{}_{tot} - \Delta G_{cor} (63)$$

where ΔG_{cor} = −1.941 eV accounts for the sum free energy corrections of equations 60, 61 and 62. Figure S18-b illustrates the framework intended for simulation, while Figure S18-c depicts the actual species that correspond to these frames, which will be explicitly evaluated with DFT.

S-3.4 Nernstian correction

The applied potential, U, is incorporated into the Gibbs free energy, together with energetic contributions arising from the activity correction of the aqueous proton, a_{H^+}. These contributions are critical, as the availability of protons in the medium directly modulates the reaction tendency. Consequently, the Gibbs free energy is expressed as a function of both the applied potential and proton activity, $\Delta G(U,\ a_{H^+})$.

For reactions involving one single PCET, specifically reductions, the Gibbs free energy can be expressed as:

$$\Delta G(U{,}a_{H^+}) = \Delta G - eU + RT\ ln a_{H^+} (64)$$

Here, e represents the elementary charge, and ΔG corresponds to the Gibbs free energy computed at pH=0 according to Equation 51, which now serves as the reference without the standard-state notation. The relationship between the proton activity, a_{H^+}, and the pH is given by a_{H^+} = 10^{-pH}. Considering the thermal energy per particle, the Gibbs free energy can thus be expressed as a function of both the applied potential and the solution pH:

$$\Delta G(U{,}pH) = \Delta G - neU + nk_BT\ ln 10 \cdot (65)$$

This formulation elegantly captures the combined influence of electrochemical potential and proton availability, providing a clear thermodynamic framework for assessing the driving force of proton-coupled electron transfer processes under varying pH conditions. The logarithmic dependence on pH emphasizes the critical role of proton concentration in modulating reaction energetics, reflecting a nuanced interplay between solution chemistry and applied potential.

In the context of the reaction considered here, we examine the PCET process:

$$* NO_2 + H^+ + e^- \rightarrow * (66)$$

whose Gibbs free energy varies as a function of both the applied potential, U, and the solution pH. In contrast, the process

$$* NO_2 + e^- \rightarrow NO_2^- (67)$$

corresponds solely to desorption and, as such, does not adhere to the thermodynamic corrections applied to the free energy of PCET steps.

Consequently, Equation 65 can be reformulated to determine the range of pH and applied potential, U, at which the PCET step becomes thermodynamically favored over desorption:

$$U = \frac{k_B T}{e} ln10 \cdot pH + \frac{1}{e}\left(\Delta G_{NO_2^-} - \Delta G_{*HONO}\right)(68)$$

This expression provides a quantitative framework for evaluating the interplay between electrochemical driving force and proton availability, allowing a rigorous assessment of the conditions under which the PCET pathway dominates the reaction landscape. Such analysis is pivotal for rationalizing selectivity in electrochemical transformations and guiding the design of efficient catalytic systems.

S-3.5 Hydrogen evolution reaction

The Hydrogen Evolution Reaction (HER) is examined as the principal competing cathodic pathway relative to the NO_3RR, and its overall reaction is

$$H^+ + e^- \rightarrow \frac{1}{2} H_2 (69)$$

This transformation is partitioned into two proton–coupled electron transfer steps, each associated with its respective free energy contribution,

$$H^+ + e^- + \ * \ \rightarrow * H, \qquad \Delta G_1 (70)$$

$$* H \rightarrow \frac{1}{2} H_2, \qquad \Delta G_2 (71)$$

Consequently, three thermodynamic states arise, as illustrated in Fig. S19-a. In the computational framework used to emulate the free-energy landscape depicted in Fig. S19-b, the chemical potential formalism introduced in Eq. 57 is required. Within this formulation, the intermediates and reference entities employed in the simulations correspond precisely to those represented in Fig. S19-c.

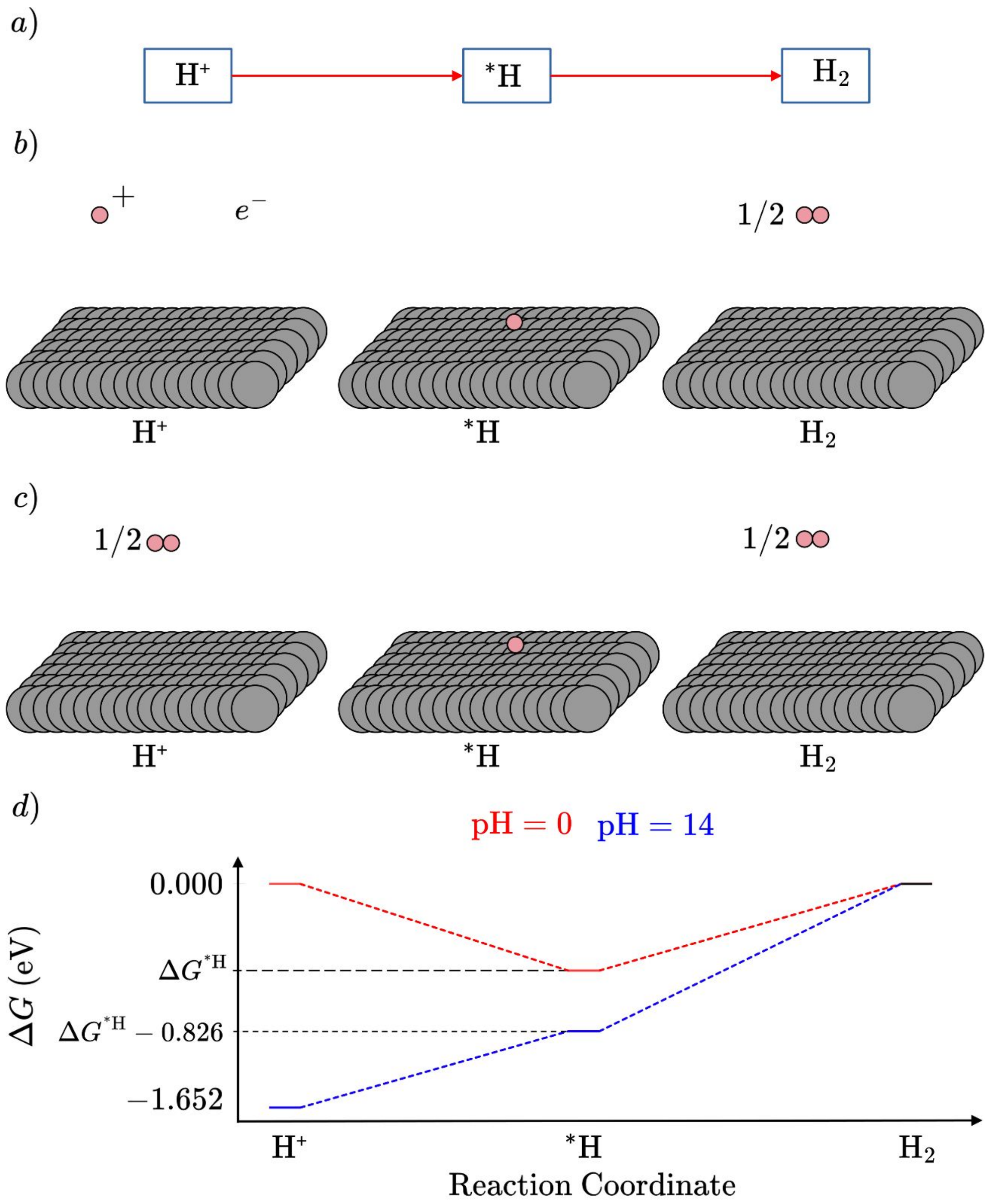

Figure S19. a) Simplified mechanism illustrating the HER pathway. b) Representative snapshots of the theoretical components in a closed system, employed in the DFT calculations within the CHE framework. c) Feasible substitutions of the components actually incorporated into the computational evaluation. Dark grey spheres represent surface atoms, and light pink spheres correspond to hydrogen. d) Generic process used to exemplify the Nernstian correction applied to the HER process.

The potential-limiting step of HER is characterized by the free-energy uphill ΔG^{PDS}, which is defined as the larger of ΔG_1 and ΔG_2. Since $G^{\mathrm{H+}}$ and G^{H2} adopt their standard values and are taken as reference ($\Delta G^{\mathrm{H+}} = \Delta G^{\mathrm{H2}} = 0$), the expression for ΔG^{PDS} simplifies to

$$\Delta G^{PDS}(pH = 0) = max\{\Delta G_1,\Delta G_2\} = max\{\Delta G^{*H}, -\Delta G^{*H}\} = |\Delta G^{*H}| \quad (72)$$

Equation 72 corresponds to standard conditions at pH = 0. Therefore, a Nernst-type correction must be applied to account for the pH = 14 environment relevant to the NO_3RR electrochemical cell. Using Eq. 65 and adapting it to the specified pH, the term k_B T ln(10) pH yields 0.826 eV for a single PCET step and 1.652 eV for two such steps. Figure S19-d illustrates how the free-energy profile evolves under these corrections while keeping ΔG^{H2} as the reference.

Accordingly, the expression for ΔG^{PDS} no longer simplifies to the absolute magnitude of $\Delta G^{*\mathrm{H}}$. Instead, it becomes

$$\Delta G^{PDS}(pH = 14) = |\Delta G^{*H}| + (73)$$

S-4 GAS-PHASE MOLECULES

The calculations of the molecular species in the gas phase were carried out using a simulation box of 20 × 20.25 × 20.5 Å using also 12.5 % larger than the recommended value of cut-off energy for oxygen (see Table S4). The corresponding binding energies and optimized geometric parameters are reported in Table S5, ensuring sufficient spatial separation to avoid spurious periodic interactions.

Table S5.. Bond lengths d^{H-H}, d^{O-H} and d^{O-O}, Molecule angle α^{mol}, binding energy E_b of the Molecule in the gas phase, and its percentage deviation ΔE_b from the reference values E_b^{ref} expressed in parenthesis.

Species	E_b (eV)	ΔE_b (%)	d^{H-H} (Å)	d^{N-O} (Å)	d^{O-H} (Å)	α^{mol} (º)
H_2	-4.53 (-4.55[25])	0.55	0.75	-	-	-
NO_2	-11.66 (-11.44[40])	1.92	-	1.21	-	1.34
trans-HONO	-14.98	-	-	1.18, 1.47 (d_1^{N-O}, d_2^{N-O})	0.98	111,101 (N, O)
H_2O	-10.13 (-10.14[25])	0.06	-	-	0.97	104
HNO_3	-18.94	-	-	1.22, 1.21, 1.44 (d_1^{N-O}, d_2^{N-O}, , d_3^{N-O})	0.98	131, 114, 102 (N_1,N_2,O)

The Gibbs free energies of the gas-phase molecules are reported in Table S6, evaluated according to Eq. 51. These values provide the reference thermodynamic baseline for subsequent adsorption energy calculations.

Table S6. Calculation results of vibrational frequencies, showing energy values for ZPE, $\int C_p dT$, $-TS$, and Gibbs energy G. All thermodynamic conditions are considered under standard settings of T =298.15 K and P =1 bar.

**Molecule**	**ZPE (eV)**	$\int C_p\, dT$ **(eV)**	**– TS (eV)**	**G (eV)**
H_2	0.265	0.09	– 0.402	– 6.804
NO_2	0.236	0.115	– 0.754	– 18.989
trans-HONO	0.528	0.115	– 0.771	– 23.150
H_2O	0.563	0.103	– 0.584	– 14.184
HNO_3	0.698	0.123	– 0.826	– 28.898

S-5 BULK PROPERTIES

Figure S20 illustrates the crystalline structure of the spinel Co_3O_4, corresponding to the Strukturbericht designation $D7_2$ and the space group $FD\overline{3}m$, depicted in both the conventional and primitive unit cells.[33] All calculations performed on the bulk phase employ the primitive unit cell. The structure of Co_3O_4 exhibits two distinct cobalt coordination environments: tetrahedral Co_2^+ and octahedral Co_3^+. The Co_2^+ ions are magnetically organized in an antiparallel arrangement (antiferromagnetic), whereas the octahedrally coordinated cobalt ions do not contribute significantly to the magnetism and display diamagnetic behavior.[34] Based on Crystal Field Theory, the Co_2^+ ion with electronic configuration [Ar]$3d^7$ in a tetrahedral ligand field exhibits a high-spin arrangement, with fully occupied e orbitals and partially filled t_2 orbitals containing unpaired electrons. In contrast, the Co_3^+ ion with configuration [Ar]$3d^6$ in an octahedral field has fully filled t_{2g} orbitals and empty e_g orbitals, leading to a diamagnetic state without unpaired electrons.

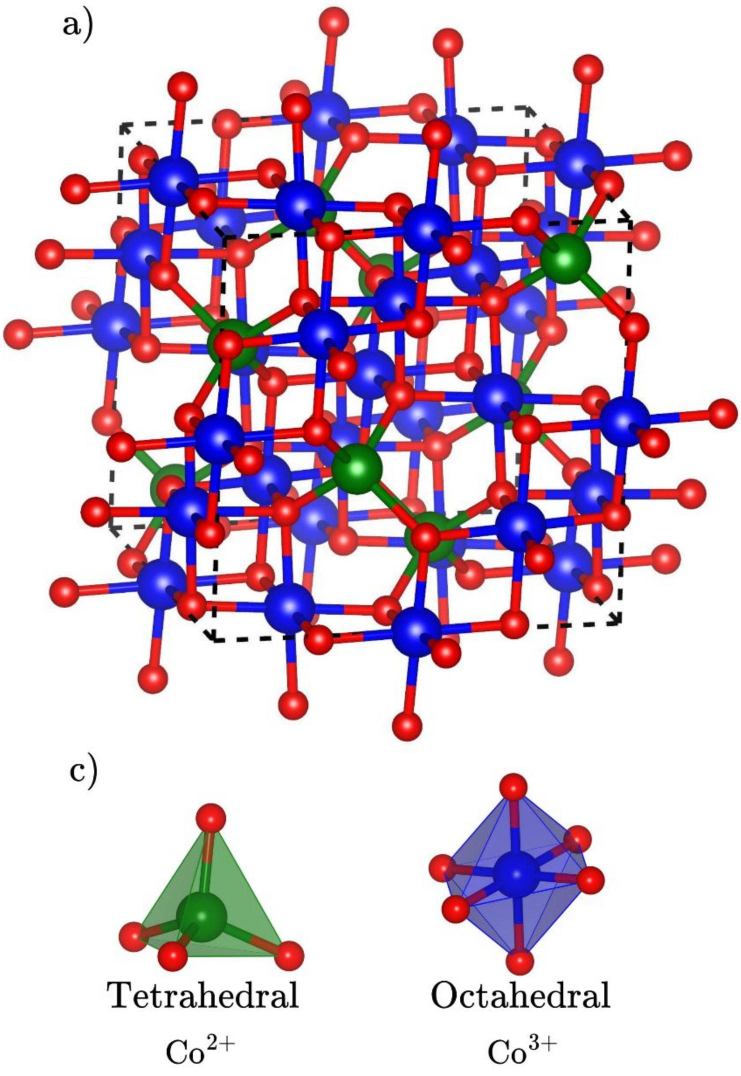

Figure S20. Crystal structure of the Co_3O_4 spinel: a) conventional unit cell, b) primitive cell, and c) distinct cobalt coordination environments. Cobalt atoms in green occupy tetrahedral sites, whereas cobalt atoms in blue occupy octahedral sites. Oxygen atoms are shown in red, and dashed lines indicate the unit cell boundaries.

The stress-tensor test for spinel Co_3O_4 was performed using a sampling of the Brillouin zone by means of k-points, expressed as a function of the length (R_k). The cutoff energy employed in the stress-tensor evaluation was 868.862 eV, corresponding to 2×*ENMAX* of oxygen, which is the highest *ENMAX* among the constituent elements, as indicated in Table S4. The outcomes of these tests are summarized in Table S7, where both Δa_0 and ΔE_{tot} are reported relative to the most converged k-point mesh.

Table S7. Convergence of the lattice parameter (a_0) and relative energy (ΔE_{tot}) for the primitive bulk spinel Co_3O_4 unit cell, obtained from stress tensor relaxation calculations as a function of the k-point mesh ($n \times n \times n$) and the total number of k-points in the Brillouin zone (N_k).

**k-points**	n	N_k	a_0 (Å)	Δa_0 (%)	ΔE_{tot} (meV)
20	4	13	8.093	0.082	0.055
40	9	85	8.092	0.094	0.023
60	13	231	8.093	0.082	0.117

80	17	489	8.099	0.000	0.000

The experimental lattice parameter of spinel Co_3O_4, as reported in the literature, is 8.0835 Å.[36] In comparison, DFT+U calculations predict a lattice parameter of 8.09 Å.[37] The deviation between these values and the lattice parameter obtained in the present work (8.09 Å) is less than 0.9 %, underscoring the excellent agreement between theoretical and experimental results, as well as the reliability of the adopted computational framework.

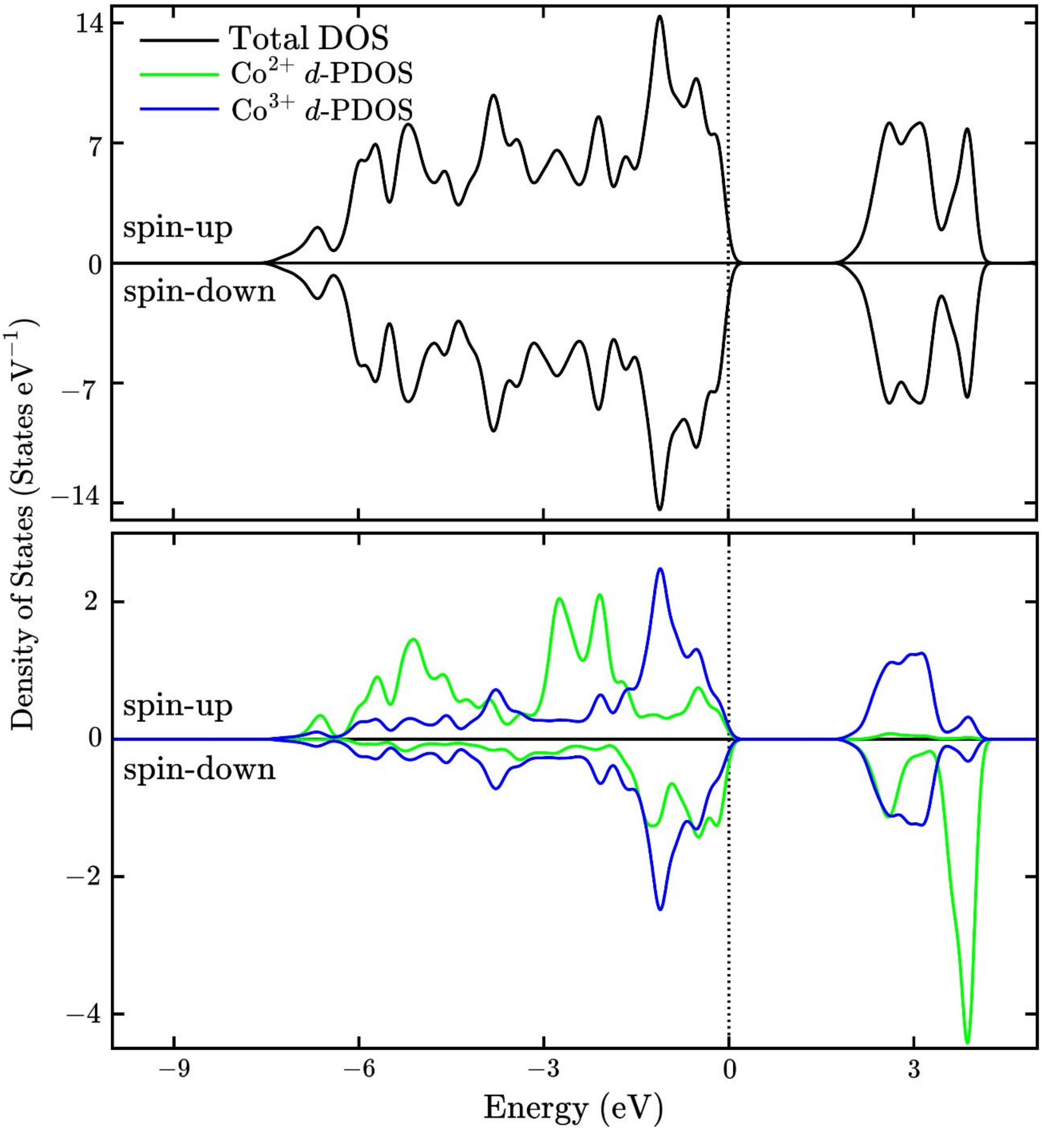

Figure S21. Total/partial Density of State (Total/P-DOS) for spinel Co_3O_4. Black dashed vertical lines represent the energy reference, which is the valence band maximum (VBM) for semiconductors.

S-6 CLEAN SURFACES

Depending on the pressure and temperature conditions, the spinel Co_3O_4 may exhibit variations in the Co to O ratio. The (111) surface admits three distinct

terminations: stoichiometric (S), reduced (R), and oxidized (O). Among these, the configurations $4Co^{T}_{3c}$ - $2Co^{O}_{3c}$, $4Co^{T}_{3c}$ - $4Co^{O}_{3c}$, and $16O_{3c}$ - $4Co^{T}_{3c}$ serve as the most representative models of the three classifications, respectively. These notations refer to the arrangement of cobalt atoms in tetrahedral coordination (Co^{T}), cobalt atoms in octahedral coordination (CoO), and oxygen atoms at the surface exposed to vacuum. The subscript $3c$ denotes atoms in threefold coordination at the vacuum interface.[41]

All calculations involving the clean surfaces were carried out with a plane-wave cut-off set to 1.125 percent of the recommended value for oxygen, employing slab models consisting of 5 atomic layers and separated by a vacuum region of 15 Å. Figure S22 illustrates the surface geometries considered in this study, providing a structural framework for the subsequent analysis.

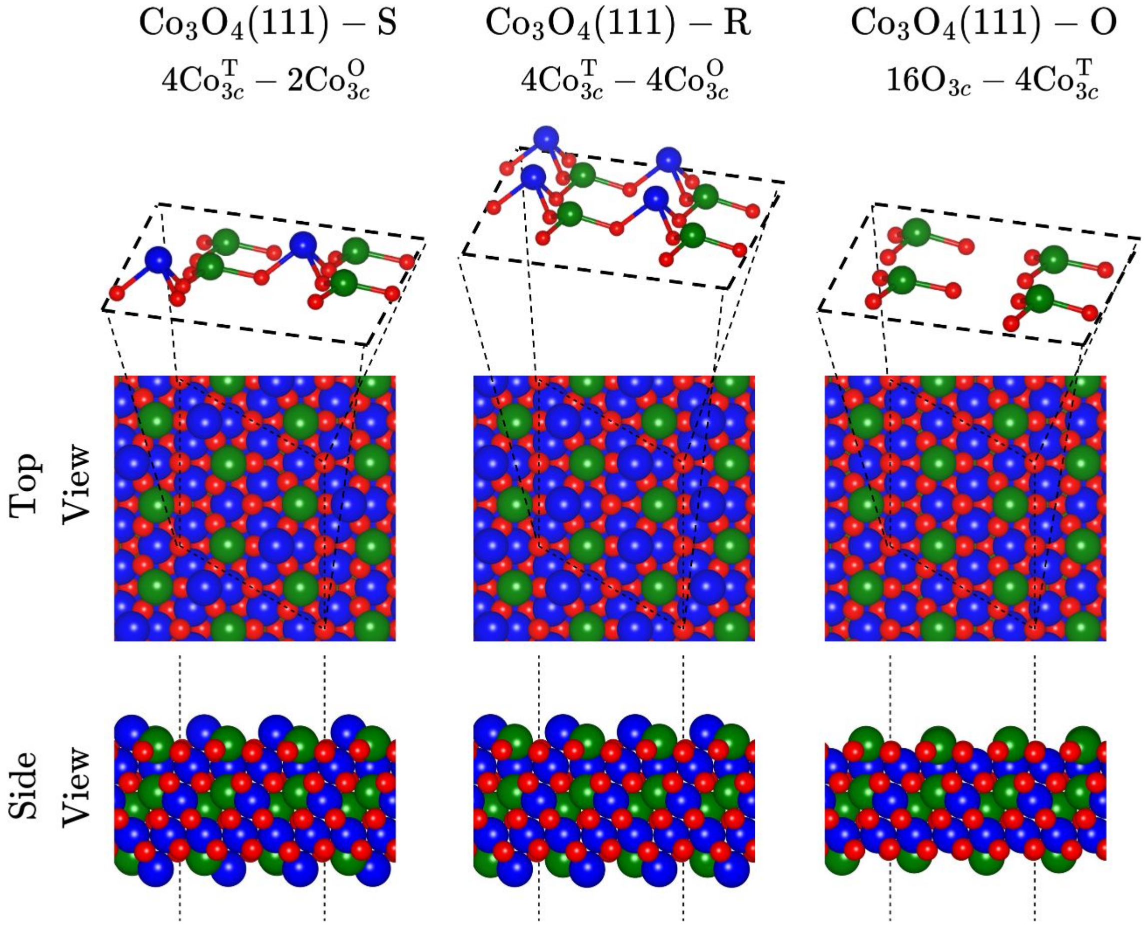

Figure S22. Top and side views of the $Co_3O_4(111)$ (1 × 1) surface, emphasizing the exposed terminations that determine the relative proportions of cobalt species. Green, blue, and red spheres represent Co^{T}, Co^{O}, and O atoms, respectively. Dashed lines indicate the boundaries of the supercell.

Table S8. Surface properties, including the surface energy (σ_S), and magnetic moment expressed in units of the Bohr magneton (μ/μ_β), were evaluated for the atoms located at the outermost layer, i.e., those directly exposed to vacuum. The calculated values for these surface atoms are compared with reference data available in the literature.[41]

Substrate	Co/O ratio	σ_S (eV)	μ/μ_B (Co^T)	μ/μ_B (Co^O)	μ/μ_B (O)
$Co_3O_4(111)$ – S ($4Co^T_{3c}$ – $2Co^O_{3c}$)	3/4	13.99	2.6 or 2	1.69 (1.69(	0.0 (0.0)
$Co_3O_4(111)$ - R ($4Co^T_{3c}$ - $4Co^O_{3c}$)	3.25/4	20.21	2.7 (2.7)	2.00 (1.69)	0.0 (0.0)
$Co_3O_4(111)$ - O ($16O_{3c}$ - $4Co^T_{3c}$)	2.75/4	26.17	2.7 (2.6)	-	0.0 (0.0)

S-7 ADSORPTION OF MOLECULES ON SURFACES

This section presents all tabulated results related to the adsorption processes required to simulate NO_3RR and HER within the defined scope of this study. The top (T) positions were explored as the initial adsorption sites for the NO_2^- and trans-HONO molecules, representing the most probable binding configurations. For a more comprehensive screening, multiple configurations were evaluated, varying both the atomic positions and molecular orientations relative to the surface, as illustrated in Figure S23.

For simplicity, the figure also provides the numerical indices of each configuration along with the adsorption site labels, ensuring a more compact and clear representation for subsequent data discussion. In the case of hydrogen adsorption, all top sites were examined; however, because hydrogen is a monoatomic adsorbate, no configurational variation was required.

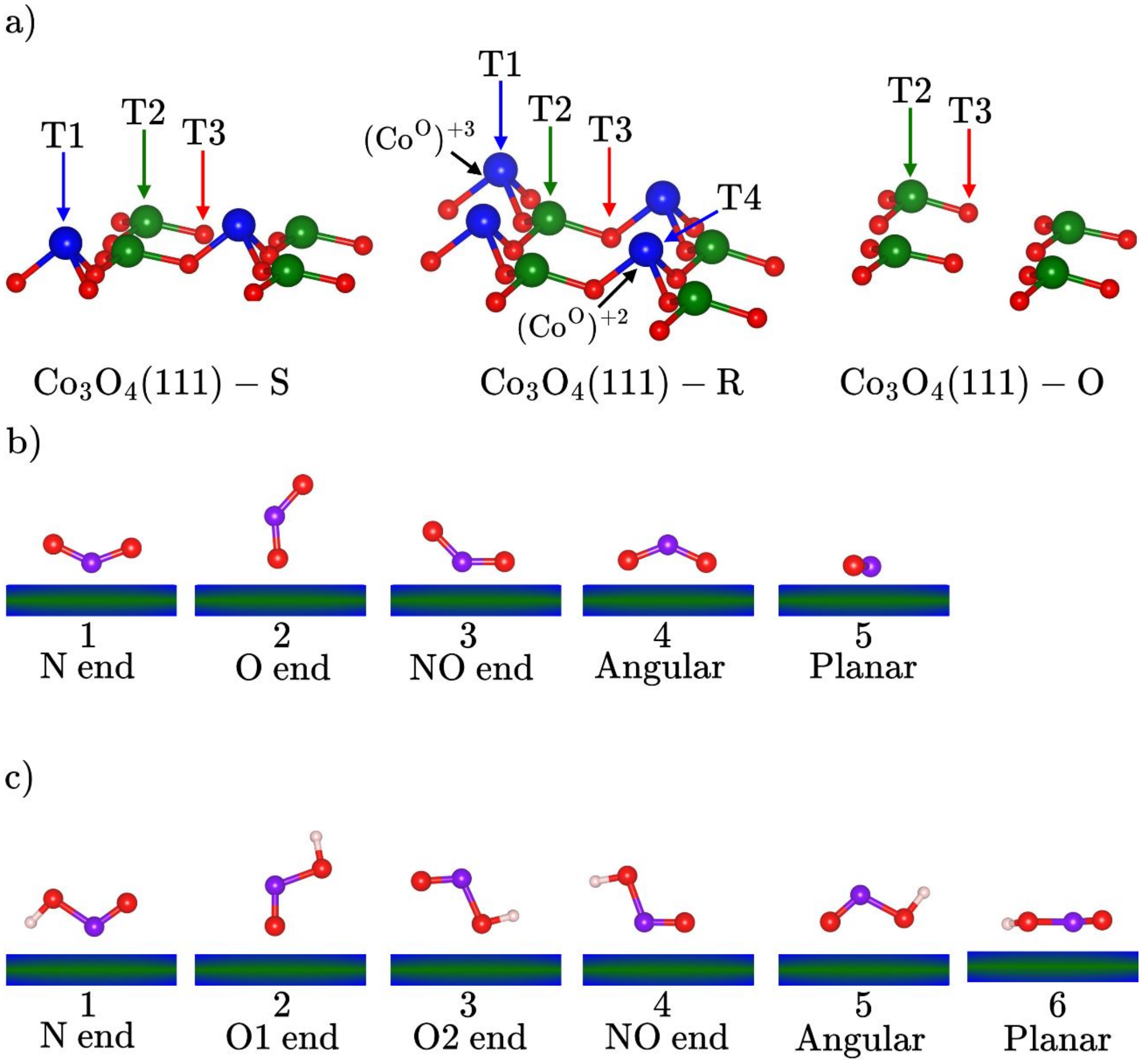

Figure S23. (a) Adsorption sites on the three $Co_3O_4(111)$ surfaces S, R and O, considered for adsorption, together with the corresponding initial configurations of (b) NO2 and (c) trans-HONO on the surface. Green, blue, red, purple and light pink spheres represent Co^T, Co^O, O, N and H atoms, respectively.

This systematic approach enables a coherent comparison between adsorption behaviors across species and surfaces, offering a consistent structural basis for the interpretation of energetic and electronic trends.

S-7.1 Molecules on $Co_3O_4(111)$-S

The results are derived from the screening of adsorbates associated with the NO_3RR and HER pathways on the $Co_3O_4(111)$-S surface.

Table S9. Geometric characteristics and adsorption energy, ΔE_{ad}, for H on $Co_3O_4(111)$-S, considering the initial adsorption sites (site i) and their corresponding final positions (site f) after full geometric optimization.

Site i	Site f	ΔE_{ad} (eV)	d^{H-Co^T} (Å)	d^{H-Co^O} (Å)	d^{H-O^s} (Å)
T1	T1	0.73	4.08	1.54	3.16
T2	T2	2.44	1.51	3.23	2.58
T3	T3	–0.96	3.76	5.79	0.98

Table S10. Geometric parameters and adsorption energies, ΔE_{ad}, for $^*NO_2$ on the $Co_3O_4(111)$-S surface are presented, taking into account the initial adsorption sites and their corresponding optimized configurations (site i-f). The structural arrangements after relaxation are indicated by labels 1–5 in parentheses, in accordance with Figure S23-b, following full geometrical optimization.

Site i-f	ΔE_{ad} (eV)	d^{O-Co^T} (Å)	d^{O-Co^O} (Å)	d^{O-O^s} (Å)	d^{N-Co^T} (Å)	d^{N-Co^O} (Å)	d^{N-O^s} (Å)	$\alpha_1^{NO_2}$ (⁰)	$\alpha_2^{NO_2}$ (⁰)
T1(1) -T1(1)	−1.24	4.81	2.67	3.75	4.51	1.88	3.41	26	93
T1(3) -T1(3)	–0.37	4.35	2.00	3.39	4.02	1.76	3.09	38	106
T1(4) -T1(4)	−1.51	3.87	1.96	3.06	4.89	2.50	3.84	25	85
T1(5) -T1(5)	–0.14	4.02	1.86	2.89	4.26	1.91	3.08	5	170
T2(1) -T2(3)	−1.78	4.08	1.90	2.79	4.21	1.99	2.75	52	90
T2(2) -T1(4)	−1.67	3.88	1.97	3.11	4.91	2.49	3.87	45	93
T2(4)-T2(2)	−1.78	3.78	2.01	2.43	5.05	3.09	3.82	73	96
T2(5)-T2(4)	−1.89	4.28	1.90	3.03	4.84	2.91	3.52	41	94
T3(1)-T2(2)	−1.78	4.05	1.90	2.78	4.17	2.00	2.75	52	95
T3(3)-T3(2)	−1.52	4.06	1.90	2.78	4.17	2.00	2.75	52	85
T3(4)-T3(4)	−1.42	4.26	1.90	3.01	4.81	2.91	3.51	19	84
T3(5)-T3(4)	−1.42	4.27	1.90	3.01	4.82	2.90	3.51	41	95

Table S11. Geometric parameters and adsorption energies, ΔE_{ad}, for trans-HONO on the $Co_3O_4(111)$-S surface are presented, taking into account the initial adsorption sites and their corresponding optimized configurations (site i-f). The structural arrangements after relaxation are indicated by labels 1–6 in parentheses, in accordance with Figure S23-c, following full geometrical optimization.

Site i-f	ΔE_{ad} (eV)	d^{O-Co^T} (Å)	d^{O-Co^O} (Å)	d^{O-O^s} (Å)	d^{N-Co^T} (Å)	d^{N-Co^O} (Å)	d^{N-O^s} (Å)	$\alpha_1^{NO_2}$ (⁰)	$\alpha_2^{NO_2}$ (⁰)
T1(1) - T1(1)	0.02	4.91	2.76	3.81	4.40	1.84	3.34	37	94
T1(2) - T1(2)	− 0.16	4.12	1.88	3.14	5.34	2.84	4.27	66	89
T1(4) - T1(1)	− 0.12	4.23	2.63	3.18	3.82	1.78	2.91	48	70

T1(5) - T1(5)	− 0.12	4.14	1.96	3.09	5.18	2.58	4.09	47	76
T2(2) - T2(2)	0.06	4.19	1.99	2.76	5.36	2.97	3.96	63	93
T2(6) - T2(1)	0.03	4.22	2.81	3.21	4.35	1.88	2.93	44	91
T3(5) - T3(5)	0.04	3.32	2.09	2.71	3.74	2.83	2.80	2	16

Table S12. The energetic contributions ZPE, $\int Cp\ dT$, and $-TS$ within the scope of the CHE model for the determination of G in systems comprising adsorbed intermediates in their most thermodynamically stable configurations, taking Co_3O_4(111)-S as the reference surface.

System	ZPE (eV)	$\int C_p dT$ (eV)	$-TS$ (eV)	G (eV)
*H	0.312	0.004	− 0.005	− 680.796
*NO_2	0.273	0.097	− 0.199	− 697.072
*ONOH	0.550	0.122	− 0.263	− 699.667

S-7.2 Molecules on Co_3O_4(111)-R

The results presented herein derive from the screening of adsorbates relevant to the NO_3RR and HER pathways on the Co_3O_4(111)-R surface.

Table S13. Geometric characteristics and adsorption energy, ΔE_{ad}, for *H on Co_3O_4(111)-R, considering the initial adsorption sites (site i) and their corresponding final positions (site f) after full geometric optimization.

Site i	Site f	ΔE_{ad} (eV)	d^{H-Co^T} (Å)	d^{H-Co^O} (Å)	d^{H-O^s} (Å)
T1	T1	− 3.23	4.11	1.60	3.30
T2	T2	− 2.84	1.62	3.54	4.19
T3	T3	− 1.89	2.59	2.69	0.98
T4	T4	− 2.25	4.01	1.59	3.29

Table S14. Geometric parameters and adsorption energies, ΔE_{ad}, for *NO_2 on the Co_3O_4(111)-R surface are presented, taking into account the initial adsorption sites and their corresponding optimized configurations (site i-f). The structural arrangements after relaxation are indicated by labels 1–5 in parentheses, in accordance with Figure S23-b, following full geometrical optimization.

Site i-f	ΔE_{ad} (eV)	d^{O-Co^T} (Å)	d^{O-Co^O} (Å)	d^{O-O^s} (Å)	d^{N-Co^T} (Å)	d^{N-Co^O} (Å)	d^{N-O^s} (Å)	α_1^t (º)	α_2^t (º)
T2(1)-T2(1)	−5.74	4.19	2.91	2.99	4.28	2.06	2.91	49	76

T2(3)-T2(1)	−6.98	3.88	2.81	2.94	3.85	1.87	2.78	20	140
T3(3)-T2(1)	−5.05	4.10	2.11	3.28	3.99	1.92	2.67	21	137

Table S15. Geometric parameters and adsorption energies, ΔE_{ad}, for trans-HONO on the Co_3O_4(111)-R surface are presented, taking into account the initial adsorption sites and their corresponding optimized configurations (site i-f). The structural arrangements after relaxation are indicated by labels 1–6 in parentheses, in accordance with Figure S23-c, following full geometrical optimization.

Site i-f	ΔE_{ad} (eV)	d^{O-Co^T} (Å)	d^{O-Co^O} (Å)	d^{O-O^s} (Å)	d^{N-Co^T} (Å)	d^{N-Co^O} (Å)	d^{N-O^s} (Å)	$\alpha_1^{NO_2}$ (º)	$\alpha_2^{NO_2}$ (º)
T1(4)-T1(1)	−4.63	4.25	2.07	2.93	3.96	2.82	2.83	0	92
T2(2)-T2(2)	−4.40	3.87	2.14	2.56	5.14	3.06	3.84	71	86
T2(4)-T2(1)	−4.85	4.15	2.92	2.94	4.28	2.03	2.85	12	112
T4(1)-T4(1)	−4.33	5.04	2.78	3.85	4.46	1.84	3.35	35	77
T4(4)-T4(1)	−4.08	5.01	2.86	3.78	4.53	1.96	3.41	28	87

Table S16. The energetic contributions ZPE, $\int Cp\, dT$, and $-TS$ within the scope of the CHE model for the determination of G in systems comprising adsorbed intermediates in their most thermodynamically stable configurations, taking Co_3O_4(111)-R as the reference surface.

System	ZPE (eV)	$\int C_p dT$ (eV)	$-TS$ (eV)	G (eV)
*H	0.239	0.002	−0.002	−698.417
*NO_2	0.273	0.097	−0.201	−718.777
*ONOH	0.552	0.120	−0.261	−720.851

S-7.3 Molecules on Co_3O_4(111)-O

The results detailed herein were acquired through the screening of adsorbed species relevant to NO_3RR and HER on Co_3O_4(111)-O.

Table S17. Geometric characteristics and adsorption energy, ΔE_{ad}, for H on Co_3O_4(111)-O, considering the initial adsorption sites (site i) and their corresponding final positions (site f) after full geometric optimization.

Site i	Site f	ΔE_{ad} (eV)	d^{H-Co^T} (Å)	d^{H-O^s} (Å)
T2	T2	1.63	1.47	2.59
T3	T3	−1.33	2.57	0.98

Table S18. Geometric parameters and adsorption energies, ΔE_{ad}, for $*NO_2$ on the $Co_3O_4(111)$-O surface are presented, taking into account the initial adsorption sites and their corresponding optimized configurations (site i-f). The structural arrangements after relaxation are indicated by labels 1–5 in parentheses, in accordance with Figure S23-b, following full geometrical optimization.

Site i-f	ΔE_{ad} (eV)	d^{O-Co^T} (Å)	d^{O-Os} (Å)	d^{N-Co^T} (Å)	d^{N-O^s} (Å)	$\alpha_1^{NO_2}$ (º)	$\alpha_2^{NO_2}$ (º)
T2(2)-T2(2)	−0.20	1.93	3.00	2.81	3.67	36	90
T2(3)-T2(1)	−0.16	2.52	3.29	1.90	3.00	18	90
T2(4)-T2(4)	0.15	2.01	2.79	2.48	3.55	46	90
T2(5)-T2(4)	0.28	1.99	2.73	2.43	3.07	36	70
T3(1)-T2(1)	−0.17	2.62	3.46	1.91	3.14	23	74
T3(2)-T2(2)	−0.19	1.94	3.00	2.82	3.70	37	90
T3(3)-T2(2)	0.13	1.88	2.74	2.72	2.64	5	90
T3(4)-T2(4)	0.12	2.00	3.21	2.45	3.26	35	71
T3(5)-T2(1)	−0.17	2.61	3.36	1.90	2.82	23	74

Table S19. Geometric parameters and adsorption energies, ΔE_{ad}, for trans-HONO on the $Co_3O_4(111)$-O surface are presented, taking into account the initial adsorption sites and their corresponding optimized configurations (site i-f). The structural arrangements after relaxation are indicated by labels 1–6 in parentheses, in accordance with Figure S23-c, following full geometrical optimization.

Site i-f	ΔE_{ad} (eV)	d^{O-Co^T} (Å)	d^{O-Os} (Å)	d^{N-Co^T} (Å)	d^{N-O^S} (Å)	α_1^t (º)	α_2^t (º)
T2(1)-T2(1)	−0.56	2.82	2.54	2.00	2.72	61	90
T2(2)-T2(2)	−0.13	2.02	3.06	2.92	3.79	43	90
T2(3)-T2(3)	−0.25	1.99	2.94	3.40	4.17	5	63
T2(4)-T2(1)	−0.56	2.83	2.57	2.00	2.73	60	90
T2(5)-T2(3)	−0.23	1.98	2.84	3.36	4.07	0	63
T3(1)-T2(1)	−0.95	2.81	2.50	1.98	2.81	64	86
T3(3)-T2(3)	−0.33	2.03	2.77	3.26	3.33	21	34

T3(4)-T2(3)	−0.44	2.08	2.68	3.21	2.99	22	26
T3(5)-T2(2)	−0.10	2.03	2.89	2.94	3.37	34	60

Table S20. The energetic contributions $ZPE, \int Cp\ dT$, and $-TS$ within the scope of the CHE model for the determination of G in systems comprising adsorbed intermediates in their most thermodynamically stable configurations, taking Co_3O_4(111)-O as the reference surface.

System	ZPE (eV)	$\int C_p dT$ (eV)	−TS (eV)	G (eV)
*H	0.313	0.004	−0.005	−656.801
$*NO_2$	0.259	0.111	−0.273	−671.092
*ONOH	0.533	0.084	−0.177	−675.937

S-8 FREE ENERGY LEVELS FOR NO_3RR PROCESSES

Table S21 presents the reaction-state values for HER and NO_3RR within the scope of the CHE model and the reaction bottleneck proposed herein. ΔG denotes the Gibbs free energy relative to a defined reference state:

$$\Delta G = G - G_{ref} (72)$$

For HER, the reference Gibbs free energy is given by

$$G_{ref} = G^{surf} + \frac{1}{2} G^{H_2} (73)$$

and for NO_3RR

$$G_{ref} = G^{*NO_2} + \frac{1}{2} G^{H_2} + G^{H_2O} (74)$$

where G^{surf} is the Gibbs free energy of the clean catalytic surface, G^{H_2} corresponds to the free energy of molecular hydrogen and G^{H_2O} corresponds to the free energy of the gas-phase of water molecules. These thermodynamic frames are selected as the initial stages immediately preceding the first PCET event, wherein, once rendered commensurate in this manner, both reactions can be consistently compared. The values reported herein are provided in the absence of an applied potential and at pH= 0, serving as the standard reference conditions.

Table S21. Performance of HER, and NO_3RR on the slabs used in the description, expressed by ΔG (with an applied potential of U = 0.0 V and pH=0), relative to the initial reaction state preceding the PCET step in both reactions.

Reaction State	Co_3O_4(111)-S ΔG (eV)	Co_3O_4(111)-R ΔG (eV)	Co_3O_4(111)-O ΔG (eV)
		**HER**	
$H^+ + e^-$	0.00	0.00	0.00
$*$ H	−0.63	−1.62	−1.00

$\mathbf{H_2}$	0.00	0.00	0.00
	**NO_3RR**		
$*\mathbf{NO_2}$	0.00	0.00	0.00
*ONHO	0.81	1.33	−1.44
NO_2^-	0.25	5.33	−1.37

S-8.1 Pourbaix Diagram

To extend the analysis beyond the pH-dependent data presented in Table S21, a Pourbaix diagram was constructed that includes the theoretical potential required for the formation of ∗HONO. This potential is determined by applying the Nernst correction (Equation 24), which enables identification of the specific conditions (pH, U) under which the ∗HONO intermediate is thermodynamically most stable and thus predominant.

$$U(pH) = -0.059 \cdot pH + \frac{1}{e} min\{\Delta G_{*NO_2} - \Delta \mathrm{G}_{*\mathrm{ONOH}}, \Delta G_{NO_2^-} - \Delta G_{*ONOH} (75)$$

This equation determines the pH-dependent slopes of the phase boundaries in the Pourbaix diagram. The first term captures the Nernstian potential shift under standard conditions (T = 298.15 K), while the second term identifies the thermodynamic minimum among the reaction Gibbs free energy differences referenced to the *ONHO adsorption state. This minimization procedure ensures that only the most thermodynamically stable surface configurations are represented. A horizontal reference line at U = 0.0 V is also included. This thermodynamic construction is essential for delineating the stability domains shown in Figure S24.

The *ONHO region is, in principle, accessible throughout the diagram; because its formation proceeds via a proton–coupled electron transfer (PCET) mechanism, its Gibbs free energy depends linearly on U. As a result, there always exists a potential at which this adsorbed species becomes thermodynamically preferred, irrespective of the magnitude or sign of the applied bias. In contrast, the desorption of NO_2^- is a purely chemical (non-PCET) step, and its thermodynamic driving force is therefore potential-independent. Consequently, the relevant thermodynamic competition at the phase boundaries occurs either between adsorbed states (*ONHO vs. *NO_2) or between an adsorbed and a solvated species (*ONHO vs. NO_2^-).

To enable a direct comparison of the thermodynamic landscapes associated with the Co_3O_4(111)-S, Co_3O_4(111)-R, and Co_3O_4(111)-O terminations, identical scaling and ranges were imposed on the ordinate axis. This consistent formatting makes the extent of the *ONHO stability domains directly discernible and permits a straightforward correlation with the catalytic performance along the reaction pathway leading to NH_3 formation. The Co^{2+}/Co^{3+} ratio is explicitly indicated as well.

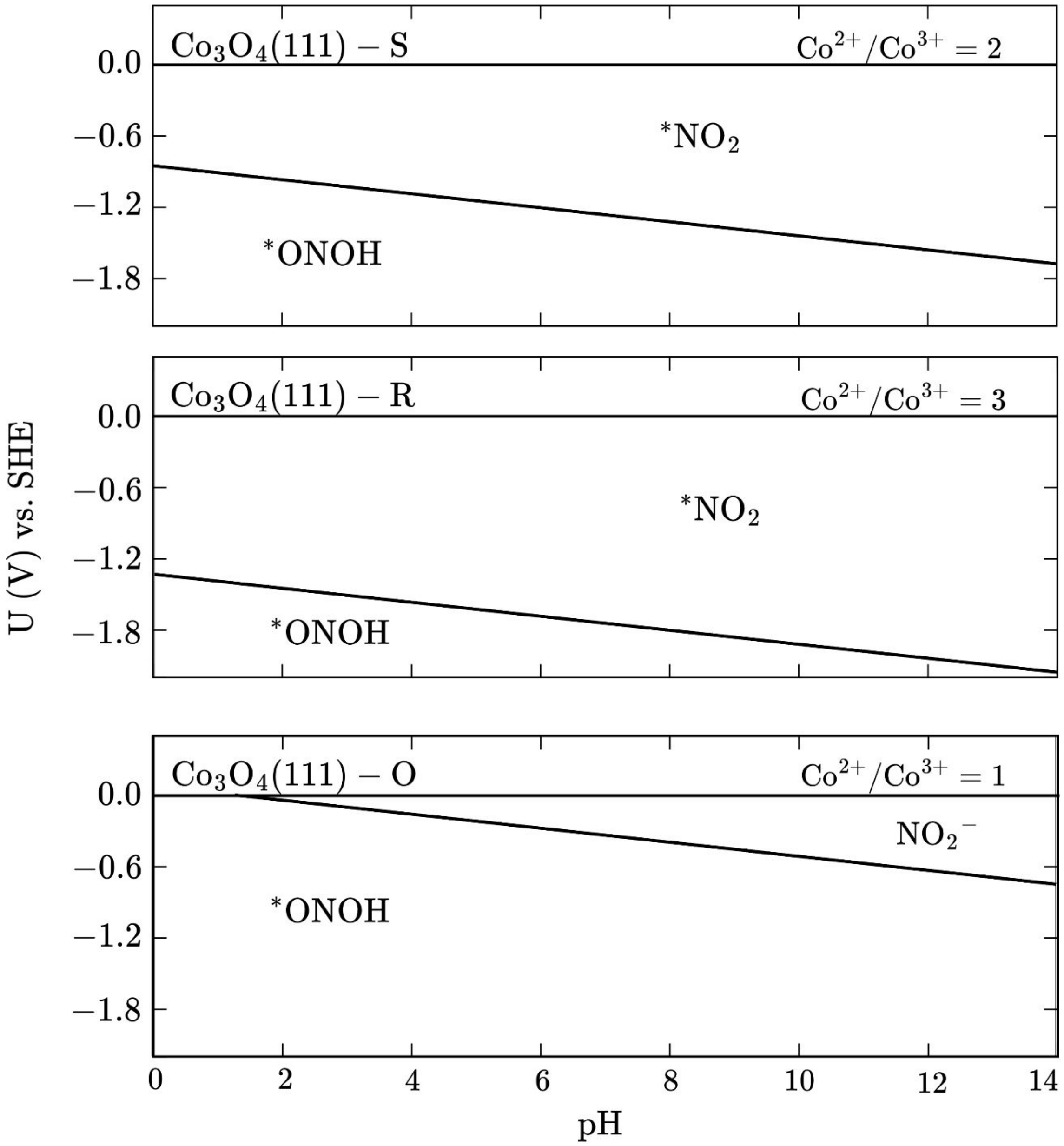

Figure S24. Pourbaix diagram determined by the theoretical potential required for the formation of the *ONOH intermediate. Contrasting the Co_3O_4(111)-S, Co_3O_4(111)-R, and Co_3O_4(111)-O surfaces, delineating the thermodynamic stability regions associated with the potential-determining steps.

The Co_3O_4(111)-S and Co_3O_4(111)-R terminations exhibit closely analogous thermodynamic profiles, characterized by a markedly restricted region of *ONOH stability. This narrow stability window indicates intrinsically suboptimal catalytic performance, which is further exacerbated by an extensive potential–pH domain in which the threshold-defining intermediate *NO_2 is thermodynamically preferred. Collectively, these features imply that these surface terminations furnish an energetic landscape highly prone to poisoning by adsorbed NO_2, while concurrently imposing substantial free-energy barriers for NO_2^- desorption.

In contrast, the Co_3O_4(111)-O termination demonstrates a markedly enhanced capacity to overcome the principal reaction bottleneck. On this surface, the stability domain of *ONOH is significantly expanded, indicating that it constitutes the most

thermodynamically favorable configuration for stabilizing this key intermediate among the terminations considered. Consequently, this facet emerges as the most promising candidate for electrocatalytic ammonia synthesis, offering a pH window of 1.28 units within which *ONOH is the dominant surface species even at open circuit (i.e., at zero applied bias). At more alkaline conditions, however, this termination exhibits a pronounced tendency toward NO_2^- desorption, thereby displaying the highest selectivity for nitrite formation among the investigated surfaces. The application of cathodic potentials can nevertheless divert the reaction flux back toward *ONOH retention, enabling a tunable selectivity that is not attainable on the more reduced counterparts.

A clear correlation is observed between an increasing Co^{2+}/Co^{3+} ratio and the requirement of more negative potentials for the reaction to proceed along the ammonia production pathway. Moreover, at lower cationic ratios (specifically $Co^{2+}/Co^{3+} = 1$), a competitive regime emerges between *ONOH stabilization and nitrite formation. Within the thermodynamic framework established by the DFT calculations, NO_2^- desorption is identified as a plausible competing pathway under these conditions.

References

(1) Adalder, A.; Paul, S.; Barman, N.; Bera, A.; Sarkar, S.; Mukherjee, N.; Thapa, R.; Ghorai, U. K. Controlling the Metal–Ligand Coordination Environment of Manganese Phthalocyanine in 1D–2D Heterostructure for Enhancing Nitrate Reduction to Ammonia. *ACS Catal.* **2023**, *13* (20), 13516–13527. https://doi.org/10.1021/acscatal.3c02747.

(2) Mukherjee, N.; Adalder, A.; Barman, N.; Thapa, R.; Urkude, R.; Ghosh, B.; Ghorai, U. K. Fe(TCNQ)$_2$ Nanorod Arrays: An Efficient Electrocatalyst for Electrochemical Ammonia Synthesis via the Nitrate Reduction Reaction. *J. Mater. Chem. A* **2024**, *12* (6), 3352–3361. https://doi.org/10.1039/D3TA05300H.

(3) Adalder, A.; Mitra, K.; Barman, N.; Thapa, R.; Bhowmick, S.; Ghorai, U. K. Magneto-Electrochemical Ammonia Synthesis: Boosting Nitrite Reduction Activity by the Optimized Magnetic Field Induced Spin Polarized System. *Adv. Energy Mater.* **2024**, *14* (42), 2403295. https://doi.org/10.1002/aenm.202403295.

(4) Iron Phthalocyanine Hollow Architecture Enabled Ammonia Production via Nitrate Reduction to Achieve 100 % Faradaic Efficiency. *Appl. Catal., B: Environmental* **2024**, *343*, 123580. https://doi.org/10.1016/j.apcatb.2023.123580.

(5) Bhowmick, S.; Adalder, A.; Maiti, A.; Kapse, S.; Thapa, R.; Mondal, S.; Ghorai, U. K. Controlling Electrocatalytic Nitrate Reduction Efficiency by Utilizing Dπ–Pπ Interactions in Parallel Stacking Molecular Systems. *Chem. Sci.* **2025**, *16* (11), 4806–4814. https://doi.org/10.1039/D4SC07619B.

(6) Winkler, M. E. G.; Yoshimura, R. G.; Rodrigues, P. S.; Sales, M. P.; Gomes, K. L.; Neckel, I. T.; Figueroa, S. J. A.; Jr, J. B. S.; Ticianelli, E. A.; Singh, N.; Lima, F. H. B.; Cherevko, S.; Nagao, R. Tailoring Reconstruction of Co/Cu Mixed Oxide-Derived Tandem Electrocatalysts via in Situ Electrochemical Dissolution-Redeposition for Enhanced Nitrate-to-Ammonia Conversion. ChemRxiv October 14, 2025. https://doi.org/10.26434/chemrxiv-2025-0nfq8.

(7) Gomes, K. L.; Winkler, M. E. G.; Sales, M. P.; Souza Junior, J. B.; Bonacin, J. A.; Nagao, R. Electrocatalytic Nitrate Reduction to Ammonia Using Co_3O_4 Nanowires Supported on TiO_2/Ti. *ACS Appl. Energy Mater.* **2025**, *8* (21), 15993–16001. https://doi.org/10.1021/acsaem.5c02502.

(8) Lu, S.; Lin, G.; Yan, H.; Li, Y.; Qi, T.; Li, Y.; Liang, S.; Jiang, L. In Situ Facet Transformation Engineering over Co_3O_4 for Highly Efficient Electroreduction of Nitrate to Ammonia. *ACS Catal.* **2024**, *14* (19), 14887–14894. https://doi.org/10.1021/acscatal.4c05292.

(9) Johnson, R. A.; Wichern, D. W. *Applied Multivariate Statistical Analysis*; Pearson: Upper Saddle River, NJ, 2007.

(10) Mc Evoy, K. M.; Genet, M. J.; Dupont-Gillain, C. C. Principal Component Analysis: A Versatile Method for Processing and Investigation of XPS Spectra. *Anal. Chem.* **2008**, *80* (19), 7226–7238. https://doi.org/10.1021/ac8005878.

(11) Figueroa, S. J. A.; Prestipino, C. PrestoPronto: A Code Devoted to Handling Large Data Sets. *J. Phys.: Conf. Ser.* **2016**, *712* (1), 012012. https://doi.org/10.1088/1742-6596/712/1/012012.

(12) Fernandez-Garcia, M.; Marquez Alvarez, C.; Haller, G. L. XANES-TPR Study of Cu-Pd Bimetallic Catalysts: Application of Factor Analysis. *J. Phys. Chem.* **1995**, *99* (33), 12565–12569. https://doi.org/10.1021/j100033a032.

(13) Harrington, D. A. Electrochemical Impedance Spectroscopy; Norwegian University of Science and Technology (NTNU): Trondheim, Norway, 2004. https://www.ntnu.edu/documents/140124/0/EIS+Literature/cd116109-3fbf-4db5-9f99-ff5ab68cf950 (accessed 2026-04-09).

(14) Lasia, A. *Electrochemical Impedance Spectroscopy and Its Applications*; Springer: New York, NY, 2014. https://doi.org/10.1007/978-1-4614-8933-7.

(15) Zhang, H.; Fang, K.; Yang, J.; Chen, H.; Ning, J.; Wang, H.; Hu, Y. Strategies and Applications of Electrocatalytic Nitrate Reduction towards Ammonia. *Coord. Chem. Rev.* **2024**, *506*, 215723. https://doi.org/10.1016/j.ccr.2024.215723.

(16) Ruthven, D. M. *Principles of Adsorption and Adsorption Processes*; Wiley-Interscience: New York, 1984.

(17) Losq, C. L. Charlesll/Spectra.Jl, 2025. https://github.com/charlesll/Spectra.jl (accessed 2025-06-04).

(18) Kresse, G.; Furthmüller, J. Efficient Iterative Schemes for Ab Initio Total-Energy Calculations Using a Plane-Wave Basis Set. *Phys. Rev. B* **1996**, *54* (16), 11169–11186. https://doi.org/10.1103/PhysRevB.54.11169.

(19) Kresse, G.; Joubert, D. From Ultrasoft Pseudopotentials to the Projector Augmented-Wave Method. *Phys. Rev. B* **1999**, *59* (3), 1758–1775. https://doi.org/10.1103/PhysRevB.59.1758.

(20) Lizárraga, R.; Pan, F.; Bergqvist, L.; Holmström, E.; Gercsi, Z.; Vitos, L. First Principles Theory of the Hcp-Fcc Phase Transition in Cobalt. *Sci Rep* **2017**, *7* (1), 3778. https://doi.org/10.1038/s41598-017-03877-5.

(21) Bader, R. F. W. *Atoms in Molecules: A Quantum Theory*; Clarendon Press: Oxford England : New York, 1994.

(22) Henkelman, G.; Arnaldsson, A.; Jónsson, H. A Fast and Robust Algorithm for Bader Decomposition of Charge Density. *Computational Materials Science* **2006**, *36* (3), 354–360. https://doi.org/10.1016/j.commatsci.2005.04.010.

(23) Hjorth Larsen, A.; Jørgen Mortensen, J.; Blomqvist, J.; Castelli, I. E.; Christensen, R.; Dułak, M.; Friis, J.; Groves, M. N.; Hammer, B.; Hargus, C.; Hermes, E. D.; Jennings, P. C.; Bjerre Jensen, P.; Kermode, J.; Kitchin, J. R.; Leonhard Kolsbjerg, E.; Kubal, J.; Kaasbjerg, K.; Lysgaard, S.; Bergmann Maronsson, J.; Maxson, T.; Olsen, T.; Pastewka, L.; Peterson, A.; Rostgaard, C.; Schiøtz, J.; Schütt, O.; Strange, M.; Thygesen, K. S.; Vegge, T.; Vilhelmsen, L.; Walter, M.; Zeng, Z.; Jacobsen, K. W. The Atomic Simulation

Environment—a Python Library for Working with Atoms. *J. Phys.: Condens. Matter* **2017**, *29* (27), 273002. https://doi.org/10.1088/1361-648X/aa680e.

(24) Blöchl, P. E. Projector Augmented-Wave Method. *Phys. Rev. B* **1994**, *50* (24), 17953–17979. https://doi.org/10.1103/PhysRevB.50.17953.

(25) Perdew, J. P.; Burke, K.; Ernzerhof, M. Generalized Gradient Approximation Made Simple. *Phys. Rev. Lett.* **1996**, *77* (18), 3865–3868. https://doi.org/10.1103/PhysRevLett.77.3865.

(26) Dudarev, S. L.; Botton, G. A.; Savrasov, S. Y.; Humphreys, C. J.; Sutton, A. P. Electron-Energy-Loss Spectra and the Structural Stability of Nickel Oxide: An LSDA+U Study. *Phys. Rev. B* **1998**, *57* (3), 1505–1509. https://doi.org/10.1103/PhysRevB.57.1505.

(27) García-Mota, M.; Bajdich, M.; Viswanathan, V.; Vojvodic, A.; Bell, A. T.; Nørskov, J. K. Importance of Correlation in Determining Electrocatalytic Oxygen Evolution Activity on Cobalt Oxides. *J. Phys. Chem. C* **2012**, *116* (39), 21077–21082. https://doi.org/10.1021/jp306303y.

(28) Wang, L.; Maxisch, T.; Ceder, G. Oxidation Energies of Transition Metal Oxides within the GGA+U Framework. *Phys. Rev. B* **2006**, *73* (19), 195107. https://doi.org/10.1103/PhysRevB.73.195107.

(29) Grimme, S.; Antony, J.; Ehrlich, S.; Krieg, H. A Consistent and Accurate Ab Initio Parametrization of Density Functional Dispersion Correction (DFT-D) for the 94 Elements H-Pu. *J. Chem. Phys.* **2010**, *132* (15), 154104. https://doi.org/10.1063/1.3382344.

(30) Bartaquim, E. O.; Bezerra, R. C.; Bittencourt, A. F. B.; Silva, J. L. F. D. Computational Investigation of van Der Waals Corrections in the Adsorption Properties of Molecules on the Cu(111) Surface. *Phys. Chem. Chem. Phys.* **2022**, *24* (34), 20294–20302. https://doi.org/10.1039/D2CP02663E.

(31) Sun, T.; Chen, R.; Ma, W.; Wang, H.; Yan, Q.; Luo, J.; Zhao, S.; Zhang, X.; Li, P. Van Der Waals Quaternary Oxides for Tunable Low-Loss Anisotropic Polaritonics. *Nat. Nanotechnol.* **2024**, *19* (6), 758–765. https://doi.org/10.1038/s41565-024-01628-y.

(32) Peraça, C. S. T.; Mocelim, M.; N. Santos, M.; Da Silva, J. L. F. Computational Investigation of the Size Evolution of $(La_2B_2O_7)_n$ Nanoclusters (B = Ce, Ti, Zr). *ACS Omega* **2025**, *10* (41), 48829–48843. https://doi.org/10.1021/acsomega.5c06927.

(33) Chen, J.; Wu, X.; Selloni, A. Electronic Structure and Bonding Properties of Cobalt Oxide in the Spinel Structure. *Phys. Rev. B* **2011**, *83* (24), 245204. https://doi.org/10.1103/PhysRevB.83.245204.

(34) Lima, A. F. Interpretation of the Optical Absorption Spectrum of Co_3O_4 with Normal Spinel Structure from First Principles Calculations. *J. Phys. Chem. Solids* **2014**, *75* (1), 148–152. https://doi.org/10.1016/j.jpcs.2013.08.015.

(35) Monkhorst, H. J.; Pack, J. D. Special Points for Brillouin-Zone Integrations. *Phys. Rev. B* **1976**, *13* (12), 5188–5192. https://doi.org/10.1103/PhysRevB.13.5188.

(36) Knop, O.; Reid, K. I. G.; Sutarno; Nakagawa, Y. Chalkogenides of the Transition Elements. VI. X-Ray, Neutron, and Magnetic Investigation of the Spinels Co_3O_4, $NiCo_2O_4$, Co_3S_4, and $NiCo_2S_4$. *Can. J. Chem.* **1968**, *46* (22), 3463–3476. https://doi.org/10.1139/v68-576.

(37) Zhandun, V. S.; Nemtsev, A. Ab Initio Study of the Magnetic, Optical and Electronic Properties of Spinel Co3O4 within DFT and GW Approaches. *J. Magn. Magn. Mater.* **2020**, *499*, 166306. https://doi.org/10.1016/j.jmmm.2019.166306.

(38) Nørskov, J. K.; Rossmeisl, J.; Logadottir, A.; Lindqvist, L.; Kitchin, J. R.; Bligaard, T.; Jónsson, H. Origin of the Overpotential for Oxygen Reduction at a Fuel-Cell Cathode. *J. Phys. Chem. B* **2004**, *108* (46), 17886–17892. https://doi.org/10.1021/jp047349j.

(39) *CRC handbook of chemistry and physics, 1995-1996.* https://hero.epa.gov/reference/3005679/ (accessed 2026-04-06).

(40) Han, Y. An Evaluation for Geometries, Formation Enthalpies, and Dissociation Energies of Diatomic and Triatomic (C, H, N, O), NO_3, and HNO_3 Molecules from the PAW DFT Method with PBE and optB88-vdW Functionals. *AIP Adv.* **2022**, *12* (12), 125009. https://doi.org/10.1063/5.0117378.

(41) Zasada, F.; Gryboś, J.; Piskorz, W.; Sojka, Z. Cobalt Spinel (111) Facets of Various Stoichiometry—DFT+U and Ab Initio Thermodynamic Investigations. *J. Phys. Chem. C* **2018**, *122* (5), 2866–2879. https://doi.org/10.1021/acs.jpcc.7b11869.
